# Identification of ZDHHC1 as a Pyroptosis Inducer and Potential Target in the Establishment of Pyroptosis-Related Signature in Localized Prostate Cancer

**DOI:** 10.1155/2022/5925817

**Published:** 2022-12-22

**Authors:** Cheng-Gong Luo, Cheng-Peng Gui, Gao-Wei Huang, Jin-Long Chen, Jia-Ying Li, Peng-Ju Li, Quan-Hui Xu, Ying-Han Wang, Jiang-Quan Zhu, Hui Liang, Zhu Wang, Qiong Deng, Jia-Zheng Cao, Jun-Hang Luo, Jun Lu, Wei Chen

**Affiliations:** ^1^Department of Urology, First Affiliated Hospital of Sun Yat-sen University, 58 Zhongshan Second Road, Guangzhou, Guangdong 510080, China; ^2^Department of Urology, Guizhou Provincial People's Hospital, 83 East Zhongshan Road, Guiyang, Guizhou 550000, China; ^3^Institute of Precision Medicine, The First Affiliated Hospital, Sun Yat-sen University, 58 Zhongshan Second Road, Guangzhou, Guangdong 510080, China; ^4^Department of Urology, Affiliated Longhua People's Hospital, Southern Medical University, Jianshe East Road, Shenzhen, Guangdong 518109, China; ^5^Department of Urology, Jiangmen Central Hospital, 23 Haibang Street, Jiangmen, Guangdong 529030, China

## Abstract

Pyroptosis or cellular inflammatory necrosis is a programmed cell death kind. Accumulating evidence shows that pyroptosis plays a crucial role in the invasion, metastasis, and proliferation of tumor cells, thus affecting the prognosis of tumors and therapeutic effects. Prostate cancer (PCa), a common malignancy among men, is associated with inflammation. Pathophysiological effects of pyroptosis on tumor development and progression, as well as the mediation of PCa, are known, but its effects on the potential prognosis for PCa warrant in-depth investigation. Herein, we built a risk model of six pyroptosis-related genes and verified their predictive abilities for prognostic and therapeutic effects. Higher risk scores indicated a higher probability of biochemical recurrence (BCR), higher immune infiltration, and worsened clinicopathological features. To derive scientific and reliable predictions for BCR in patients having PCa, the findings of the current study were verified in the Gene Expression Omnibus (GEO) cohort following evaluation in The Cancer Genome Atlas (TCGA) dataset. Additionally, after evaluating the six genes in the model, ZDHHC1 was found to be an important component. Its antitumor role was further assessed through in vivo and in vitro experiments, and its promoting effect on pyroptosis was further evaluated and verified. The above results provided a new perspective for further studies on pyroptosis and its clinical utility for PCa.

## 1. Introduction

Prostate cancer (PCa) is a frequent tumor among adult men [1]. In 2020, nearly half of the most common cancers in men were those of prostate, lung, and colorectal tumors, whereby PCa accounted for more than 20% of newly diagnosed cases [2]. The majority of these patients with localized cancers undergo standard treatments, including radiation therapy or radical prostatectomy (RP) [3]. However, in approximately 20-30% of PCa patients, biochemical recurrence (BCR) has been reported [4]. In the absence of secondary treatment, it might take 5-8 years for clinical progression of BCR patients after therapy, and nearly 32-45% of these patients are at risk of death within 15 years [5]. Typically, clinical stage, prostate-specific antigen (PSA) level, treatment modality, and Gleason's score are considered as factors influencing BCR in patients with PCa [6]. However, precisely forecasting the BCR probability among these patients under different conditions is not within the scope of the available literature. Genetic biomarkers can predict PCa recurrence, but their utility in medical practice is negligible, with almost all of them confined to the stage of molecular research. Therefore, developing a novel signature or identifying biomarkers for prognostic prediction with strong specificity and high accuracy is critical for guiding treatment in PCa.

In host cells, *Shigella dysenteriae* activates caspase-1 [7]. Caspase-1 knockout blocked cell death due to *Salmonella* [8]. In 2001, Cookson and Brennan coined the term pyroptosis for proinflammatory programmed cell death [9]. Pyroptosis is inflammasome-induced programmed cell death mediated by gasdermins [10]. Pyroptotic cells show membrane-pore formation and undergo cytoplasmic swelling, leading to a loss of plasma membrane integrity, ultimately resulting in cytoplasmic leakage. Pyroptosis occurrence requires the activation of caspase-1, necessary for the maturation of proinflammatory cytokines, including interleukin 18 (IL-18) and IL-1*β*, through inflammasome-dependent pathways [11]. Gasdermin D (GSDMD) locks into the plasma membrane after being cleaved by activated caspase-1, resulting in pore formation [12]. Growing pieces of evidence confirm that caspase-3 activates gasdermin superfamily proteins, including gasdermin E (GSDME), ultimately resulting in pyroptosis [13, 14]. Numerous studies confirm that pyroptosis is crucial for invasion, metastasis, and proliferation of tumor cells. In non-small-cell lung cancer, p53, a transcription factor, inhibits tumor cell growth through enhanced pyroptosis [15]. A novel pyroptosis-associated gene (PRG) signature for prognostic prediction in gastric cancer is previously reported [16]. However, to date, only a few studies have reported the influence of pyroptosis in PCa, and the utility of PRG signature in the prognostic prediction of PCa remains unclear. Moreover, only a few studies have constructed PRG signatures for prognostic prediction of PCa; the validation of important components of the signatures and prognostic prediction after radical treatment of localized PCa remain unknown. Therefore, evaluating the impact of pyroptosis on the development and tumorigenesis of PCa may underlie implications for the evaluation of recurrence and prognosis in patients, facilitating a better understanding of the metastasis and progression of PCa, along with identification of new therapeutic targets for treatment guidance.

We used PCa datasets from relevant databases to identify the PRGs involved in long-term BCR; according to the six prognostic features of PRGs, a signature was constructed to better identify the risk of BCR after radical prostatectomy (RP). We also conducted preliminary experimental validation of the important components in the model; pyroptosis may have a positive impact on the occurrence and development of PCa, thus guiding the treatment of these patients. The results suggested the key role of pyroptosis in the postoperative management of PCa and the prediction of future trends.

## 2. Materials and Methods

### 2.1. Data Collection

We obtained the complete transcriptome of PCa (PRAD) from The Cancer Genome Atlas (TCGA) in the FPKM-standardized format along with the corresponding clinical characteristics for 496 patients. GSE54460 with complete clinicopathological and corresponding mRNA expression profiles in the Gene Expression Omnibus (GEO) database was the validation set comprising normalized Log2 data of 107 patients. A total of 203 PRGs were extracted from GeneCards (https://www.genecards.org/), the Reactome database (https://reactome.org/), and Molecular Signatures Database (https://www.gsea-msigdb.org/) (Supplementary Table 1).

### 2.2. Gene Set Enrichment Analysis

To validate the association of differentially expressed genes (DEGs) with pyroptosis, first, the genetic symbol was converted to the entrezID with the hs.eg.db package. Gene Ontology (GO) annotation was conducted to assess the enrichment degree of the GO terms for DEGs and KEGG enrichment analysis for evaluating the function in gene sets (clusterProfiler package). *P* value < 0.05 indicated statistical significance. Finally, the bubble diagrams were drawn to visualize these results.

### 2.3. Construction for the Pyroptosis-Associated Prognostic Signature

The risk prognostic model was established using TCGA cohort. DEGs among PRGs between PCa and adjacent normal tissues were assessed using “LIMMA.” False discovery rate (FDR) < 0.05 and Log2|*fold* *change*| > 1 were the threshold values. Univariate Cox regression analysis unveiled that PRGs were linked to BCR in PCa. Upon taking the intersection of the results from the two analyses, an interaction network of proteins was constructed using the STRING database 5, and the expression correlational network was established according to the intersection results. Least absolute shrinkage and selection operator (LASSO) regression was performed, and overfitting in the ultimate prediction model was avoided. Below is the calculation performed:

(1) $$\begin{array}{c} \text{Risk score}=\alpha \text{gene }\left(a\right)\times \text{gene expression }\left(a\right)+\cdots +\alpha \text{gene }\left(n\right)\times \text{gene expression }\left(n\right). \end{array}$$

To distinguish between patients at low and high risk, the median value was set as the threshold.

### 2.4. Constructing and Validating the Prognostic Signature

TCGA and GEO cohorts were used, respectively, for constructing and validating the prognosis model by the same statistical method. Next, the Kaplan-Meier (K-M) analysis with a log-rank test for significance was performed to estimate the differences between the risk groups in BCR-free survival (bRFS). For understanding the specificity and sensitivity of survival predictions, a receiver operating characteristic (ROC) curve was plotted. Finally, the area under the ROC (AUC) curve was computed as an indicator of the prediction accuracy for each group. Independent prognostic factors for BCR of PCa were screened by univariate and multivariate Cox regression analyses. Correlations between risk scores and signature components were shown using heatmaps.

### 2.5. Immune Infiltration

Single-sample GSEA was conducted to estimate immune cell infiltration based on the model [17]. We quantified the levels of enrichment of 13 immune-related pathways and 16 immune cell types in PCa samples. Box plots were drawn to present these results.

### 2.6. Cell Lines, Culture Conditions, and Treatments

From American Type Culture Collection (ATCC), the following cell lines were acquired: RWPE-1 (human normal prostate epithelial cells), PC3 and DU145 (PCa cells), and HEK-293T cells. RWPE-1 cells were grown in keratinocyte serum-free medium (GIBCO) (with 5 ng/mL epidermal growth factor and 50 *μ*g/mL bovine pituitary extract). Both PCa cell lines and HEK293T cells were grown in MEM and DMEM supplemented with 10% FBS (Thermo Fisher Scientific and 1% penicillin/streptomycin (GIBCO)), respectively. The cells were cultured in a humidified atmosphere with 5% CO_2_ at 37°C.

### 2.7. Quantitative RT-PCR (qRT-PCR)

The reactions were performed on the QuantStudio 5 Real-Time PCR instrument (Thermo Fisher, USA) using 2x SYBR Green Pro Taq HS Premix II (AgBio, China). The primer sequences were as follows: ZDHHC1forward-1: CAAGCCCTCCAACAAGACG, reverse-1: CCAAAGCCGATCACAGCAAAG; forward-2: GTGCGGGACAAGAGCTATG, reverse-2: AGTTGCAGTGCAGGTCTTCAA; forward-3: CAACTTGTGCAACGTGGATGT, reverse-3: AAGAGCCGGTAGTTCCGCT; and GAPDH forward: GGAGCGAGATCCCTCCAAAAT, reverse: GGCTGTTGTCATACTTCTCATGG. Normalization of mRNA levels was against GAPDH levels. For calculating the relative gene expression, the 2-*ΔΔ*CT approach was employed. (First, for all test samples and calibration samples, the CT value of the internal reference gene is normalized to the CT value of the target gene: ΔCT (test) = CT (target, test)–CT (ref, test); ΔCT (calibrator) = CT (target, calibrator)–CT (ref, calibrator). Second, normalize the *Δ*CT value of the test sample with the *Δ*CT value of the calibration sample: ΔΔCT = ΔCT (test)–ΔCT (calibrator). Finally, calculate the expression level ratio: 2 − ΔΔCT = the ratio of the amount of expression.)

### 2.8. Western Blot Analysis

Thermo Fisher Scientific's BCA kit was used for protein quantification, which was preceded by the addition of 1% protease inhibitor-containing RIPA lysis buffer for protein extraction. Then, 30 *μ*g protein separated by 10% SDS-PAGE was transferred onto Millipore's PVDF membranes. At room temperature, proteins were blocked in 5% skimmed milk solution for 1 h following which these were incubated with ZDHHC1 (1 : 1000, Proteintech), cleaved caspase-3, cleaved caspase-1, GAPDH (1 : 1000, Cell Signaling Technology), GSDME-N, ASC, NLRP3, GSDMD-N, IL-1*β* (1 : 1000, Abcam), or IL-18 (1 : 200, Abcam) primary antibodies overnight at 4°C, as indicated. Subsequently, horseradish peroxidase- (HRP-) bound goat anti-rabbit antibody or HRP-bound goat anti-mouse secondary antibody (1 : 5000, Proteintech) was added to blots and incubated at room temperature for 1-2 h. Finally, Millipore's Western Chemiluminescent HRP substrate was used to observe the protein bands.

### 2.9. Plasmid Construction and siRNA Interference Assay

Human ZDHHC1 cDNA overexpression construct was synthesized and cloned into the TK-PCDH-copGFP-T2A-Puro vector by TSINGKE (Nanjing, China) and confirmed by sequencing. HEK-293T cells were cotransfected with TK-PCDH-copGFP-T2A-Puro-ZDHHC1 or empty plasmid with RSV-Rev, CMV-VSVG, and pMDLg/pRRE vectors using Lipofectamine 2000 (Invitrogen). As the negative control, an empty plasmid was used. Lentiviruses were harvested 48 h later. PCa cells were infected with lentiviruses with 8 mg/mL polybrene by ViraPower Packaging Mix (Thermo Fisher). Stable cell lines were obtained by treatment with 2 *μ*g/mL puromycin (Sigma-Aldrich) for 2 days. For interference assays, RiboBio (China) synthesized one negative control and two targeting siRNA constructs (Supplementary Table 2). siRNAs were transfected into cells using Lipofectamine 2000 following the kit protocol. Functional assays were conducted 48 h posttransfection. Total RNA and proteins were subsequently extracted.

### 2.10. Construction of Stable Strains Carrying Luciferase

PCa cells were infected with luciferase-carrying viruses overexpressing ZDHHC1 and controls (Gene, Shanghai, China) with 8 mg/mL polybrene by ViraPower Packaging Mix (Thermo Fisher). Likewise, stable cell lines were obtained by treatment with 2 *μ*g/mL puromycin (Sigma-Aldrich) for 2 days.

### 2.11. Cell Migration and Invasion Assays

Cell invasion assay was conducted in Corning's Chambers (8 *μ*m pore size, USA) containing 2% Matrigel, while migration was assessed in a chamber without the 2% Matrigel. To evaluate cell migration and invasion, Transwell assays were performed; briefly, to the upper chamber, a 200 *μ*L serum-free medium (with approximately 5 × 10^4^ cells) was added. Medium (800 *μ*L) containing 10% FBS, a nutritional attractant, was placed in the lower chamber. The cells were fixed with 4% polyformaldehyde and stained with 0.4% crystal violet solution for 20 minutes (Beyotime), following incubation for 18 h (for DU145) and 24 h (for PC3). In the upper chamber, the cells were gently wiped off with cotton swabs, and under an Olympus IX83 inverted microscope (Japan), five random fields were captured and used to calculate the proportions of invaded/migrated cells.

### 2.12. CCK-8 and Colony Formation Assays

The CCK-8 assay was conducted following APExBIO's instructional guide. The cells (2000 cells/well) were incubated in 96-well plates for 1-4 days, as indicated. Each group contained five replicates. Subsequently, Spark 10 M (Tecan) was used for assessing the optical density (OD) at 450 nm. OD values at different time points were used to plot cell growth curves. Cell viability and proliferation were evaluated.

In the colony formation assays, the cells were digested, counted, triturated to obtain a single-cell suspension, and seeded in six-well plates (1000 cells/well). After incubation for 7-14 days, the clones were washed with 1x PBS, fixed with 4% polyformaldehyde, and stained with 0.4% crystal violet solution for 20 minutes. Finally, the clones were imaged and quantified.

### 2.13. Wound Healing Assay

Before the test, at the back of a 6-well plate, a horizontal line was marked. The cells were inoculated in a 2-3 mL culture medium. When the cells reached confluency, 10 *μ*L pipette tips were used for wounding with at least three scratches per well. The cells were washed thrice in 1% PBS for removing floating debris and cultured in a serum-free medium. Finally, wound healing was observed under the IX83 microscope (Olympus, Japan), and images were obtained at 0 h and 24 h after wounding.

### 2.14. Transmission Electron Microscopy

Healthy-looking cells were selected (the cell volume was 5 × 10^6^/mL or 10 cm dish covered with grass). The culture medium was discarded, and 1% PBS was added to the wells to gently scrape the cells. These were collected into 1.5 m LEP (caution was taken not to scrape repeatedly to avoid cell scratching). The collected cells were centrifuged for 5 min (9000 × *g*); the supernatant was discarded. Gently, 2% glutaraldehyde was added to the pellet-containing cells (caution was taken to avoid cell scattering) and incubated for 30 min at room temperature before storing at 4°C until assessment by transmission electron microscopy.

### 2.15. Animal Experiments

BALB/c nude mice (4-6 weeks old) were procured from Charles River Laboratories. The xenotransplantation model was constructed by subcutaneous injection of 5 × 10^6^ DU145 cells stably overexpressing ZDHHC1 or control cells in excellent condition into the left flank of male nude mice. The tumor volume was assessed every seven days using calipers (length × width 2)/2. We dislocated the cervical spine of the mice and euthanized them, 28 days after implantation. Finally, we removed, fixed, weighed, photographed, and preserved the xenografts. All animal experimental protocols adhered to the established guidelines and received approval from the Institutional Animal Care and Use Committee of Sun Yat-sen University.

The Matrigel (1 : 1) mixtures with 1 × 10^6^ DU145 cells with or without lentiviruses stably overexpressing ZDHHC1 were orthotopically injected into the anterior prostate. After four weeks of implantation, tumor formation and sizes were assessed using a noninvasive in vivo imaging system (IVIS). Subsequently, tumor tissues were removed, fixed, and embedded in paraffin after sacrificing these mice.

### 2.16. Statistical Analysis

GraphPad Prism 8.0 and R3.6.1 software were adopted for all statistical analyses. We use the independent sample *t*-test to analyze continuous variables having normal distribution and homogeneous variance; otherwise, the Wilcoxon rank-sum test would be used. Meantime, in correlation analysis, Pearson's correlation coefficient test was conducted. According to multivariate Cox proportional hazards analysis results, we proposed a nomogram to forecast the rate of bRFS at 1, 3, and 5 years. Moreover, we used consistency index (C index), ROC, and calibration curve in order to assess the predicative ability of nomogram. For all quantitative experimental data, the experiment was repetitively conducted thrice, and the results were indicated by mean ± standard deviation. The *P* value < 0.05 presented statistical significance (^∗^*P* value < 0.05; ^∗∗^*P* value < 0.001; ^∗∗∗^*P* value < 0.0001).

## 3. Results

### 3.1. Screening the Differentially Expressed PRGs

**Figure 1:**
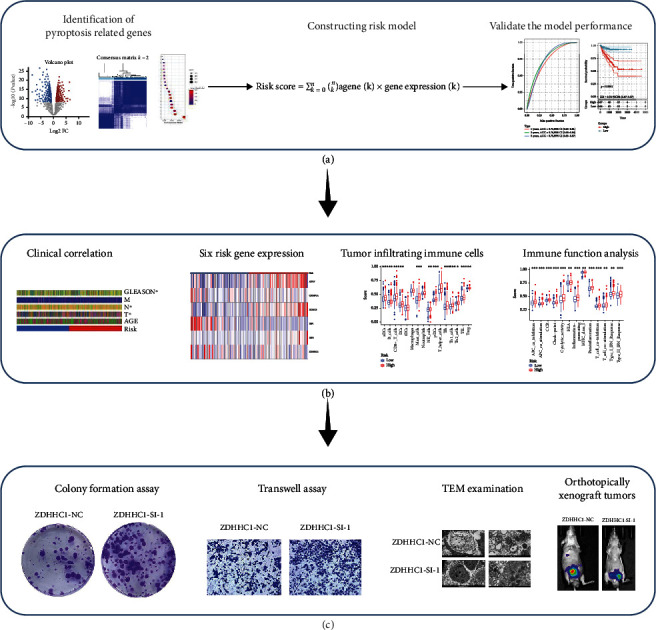
The flowchart of the study. (a) Identifying differential pyroptosis-related risk genes and constructing the risk model. (b) Comparing between the subgroup based on the risk model. (c) Selecting the key gene and performing the molecular experiments.

**Figure 2:**
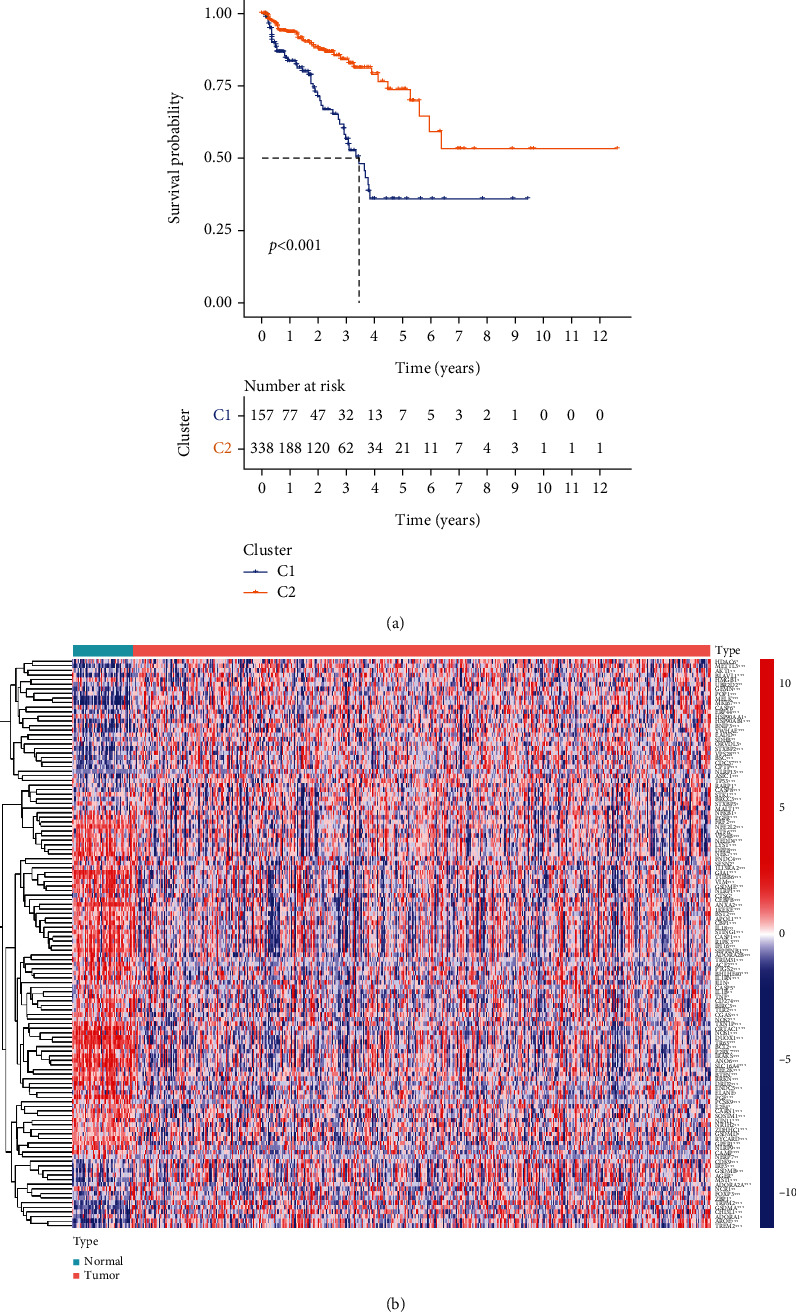
The landscape of potential prognostic PRGs in prostate cancer. (a) The Kaplan-Meier curves of DFS (disease-free survival). (b) Heatmap of differentially expressed PRG.

**Figure 3:**
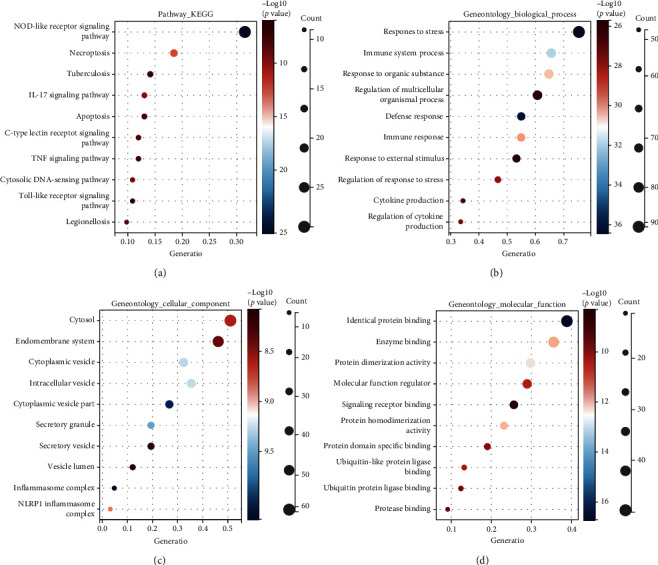
Potential functional enrichment analysis of PRG. (a) KEGG analysis. (b–d) GO analysis.

**Figure 4:**
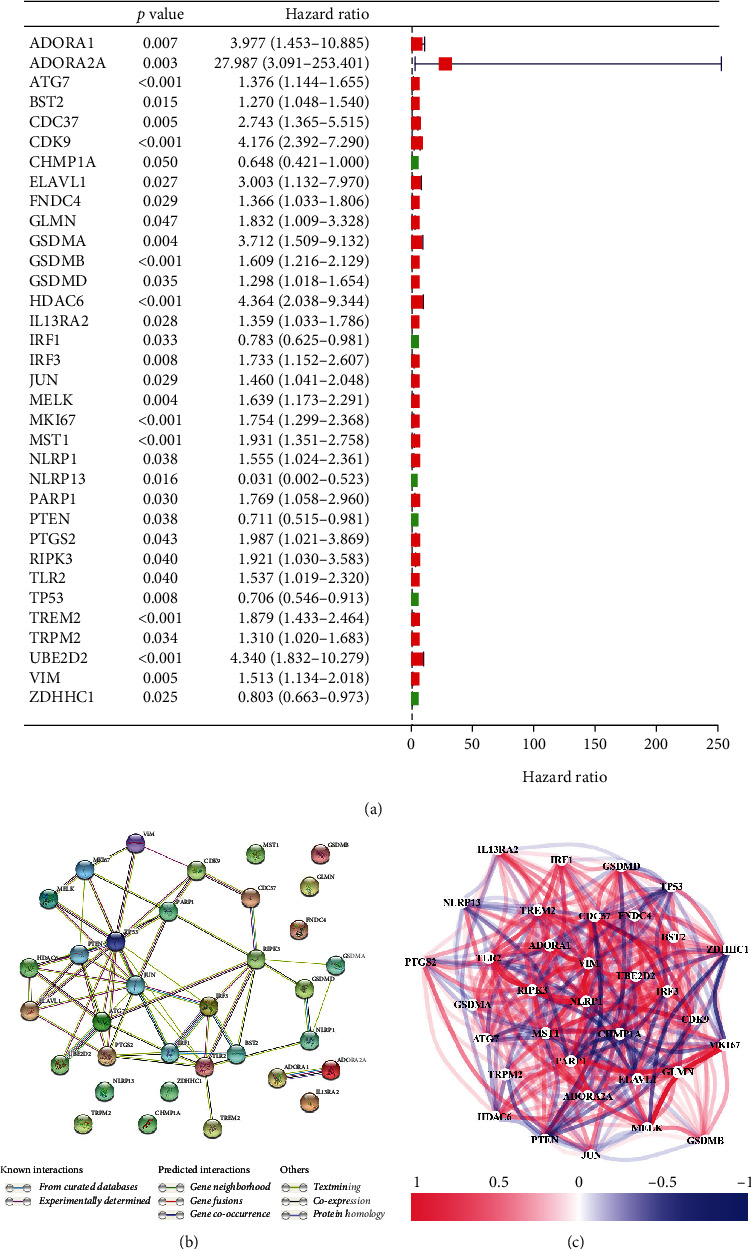
The network of candidate genes and construction of the signature in TCGA cohort. (a) 34 PRGs associated with BCR of PCa patients through univariate Cox regression analysis (red represents risk factors (hazard ratio > 1) and green represents protective factors (hazard ratio < 1). (b) The construction of PPI network by STRING (network nodes are proteins, and connections between proteins indicate predicted functional associations). (c) The gene expression correlation network suggested a strong correlation between these genes.

Figure 1 shows the flowchart of this study. The approximate process is divided into three parts, namely, identifying differential pyroptosis-related risk genes and constructing the risk model, comparing between the subgroup based on the risk model, and selecting the key gene and performing the molecular experiments. To identify the grouping structure of the data, cluster analysis was performed for 203 PRGs which indicated higher similarity among objects in the same group and greater differences among objects from various groups (Figure 2(a) and supplementary Figure 1(a)-1(f)). A total of 124 DEGs were identified, of which 80 were downregulated while 44 were upregulated in tumor tissues relative to normal tissues from TCGA cohort (*P* value < 0.05) (Figure 2(b) and Supplementary Table 3). According to GO annotation and KEGG analysis, DEGs were associated with pyroptosis (Figures 3(a)–3(d)). We found that these PRGs were mainly involved in the NOD-like receptor signaling pathway (specific families of pattern recognition receptors are responsible for detecting various pathogens and generating innate immune responses. It can activate caspase-1 to regulate the maturation of the proinflammatory cytokines IL-1B and IL-18 and drive pyroptosis), response to stress (BP), cytosol (CC), and identical protein binding (MF) in GO analysis. To screen prognostic genes related to bRFS, 34 PRGs (analyzed by univariate Cox regression) were determined as candidates and used subsequently for constructing the prognostic model (Supplementary Table 4). Twenty-eight genes served as risk factors (hazard ratio > 1) while the remaining six genes as protective factors (hazard ratio < 1) (Figure 4(a)). These 34 bRFS-related genes were uploaded to STRING for obtaining the protein-protein interaction (PPI) network. Network nodes are proteins, and each node represents all proteins produced by a protein encoding gene. Connections between proteins represent predicted functional associations, which are specific and meaningful (Figure 4(b)). Moreover, at the transcriptional level, a strong correlation between these genes was observed (Figure 4(c)). To compute the collinearity of these 34 genes, the real bRFS effectors were identified using LASSO Cox regression analysis and ultimately a prognostic panel for 6 PRGs (supplementary Figure 2(a), 2(b)) was obtained as follows: risk score = ATG7 × (0.762585776268797) + CHMP1A × (−0.0506368584322651) + HDAC6 × (0.355120084062333) + IRF1 × (−0.115816956682387) + IRF3 × (0.10545094919479) + ZDHHC1 × (−0.154328796504204).

### 3.2. Construction and Validation for the Prognostic Signature

**Figure 5:**
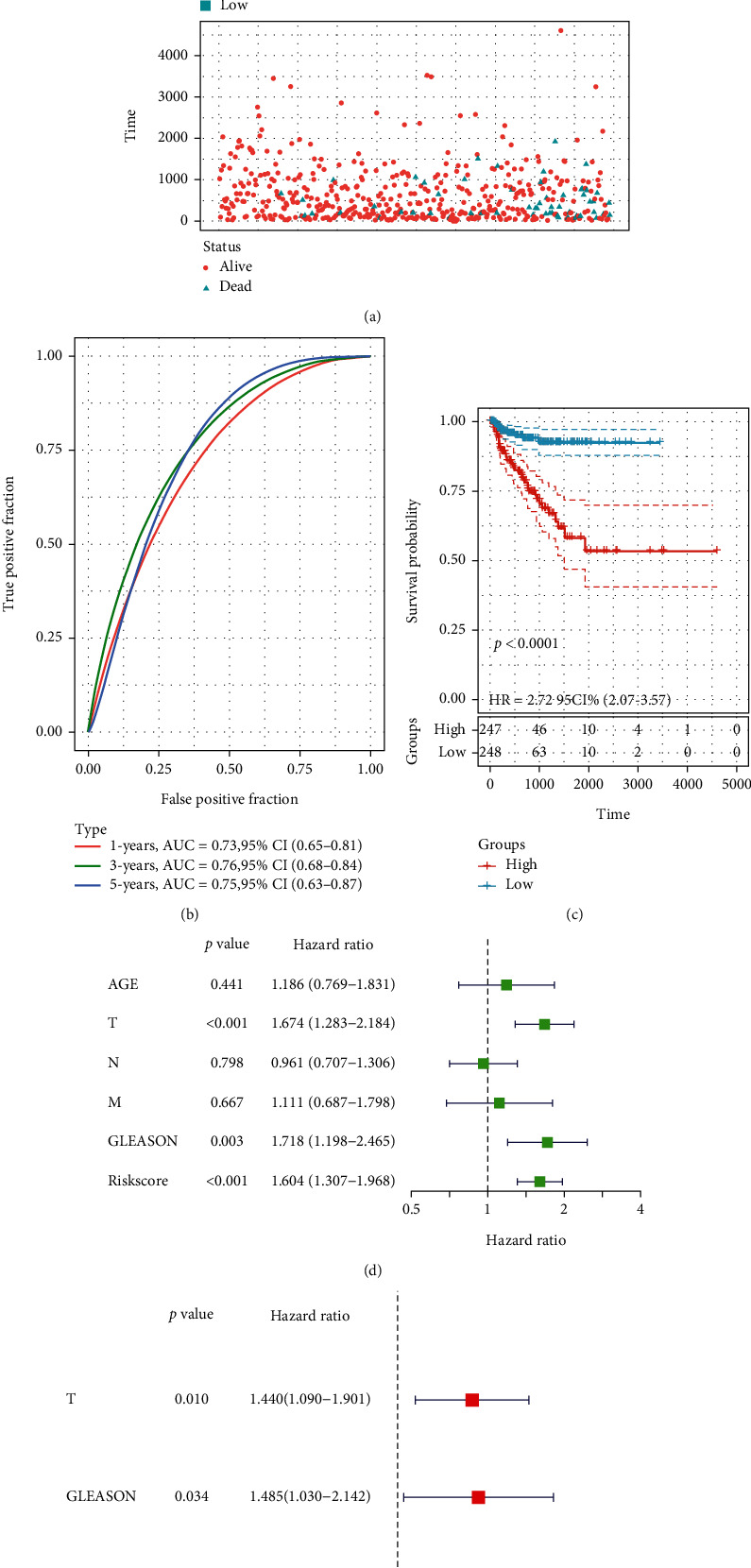
The evaluation of prognostic signature in TCGA cohort. (a) The curve of risk score and BCR status of the patients. (b) ROC curve of model and clinical characteristics predicting 1-, 3-, and 5-year bRFS. (c) The bRFS analysis of the two subgroups stratified based on the median of risk scores calculated by the risk model. (d, e) Univariate and multivariate Cox regression analyses showed that the risk score had prominent prognostic values.

**Figure 6:**
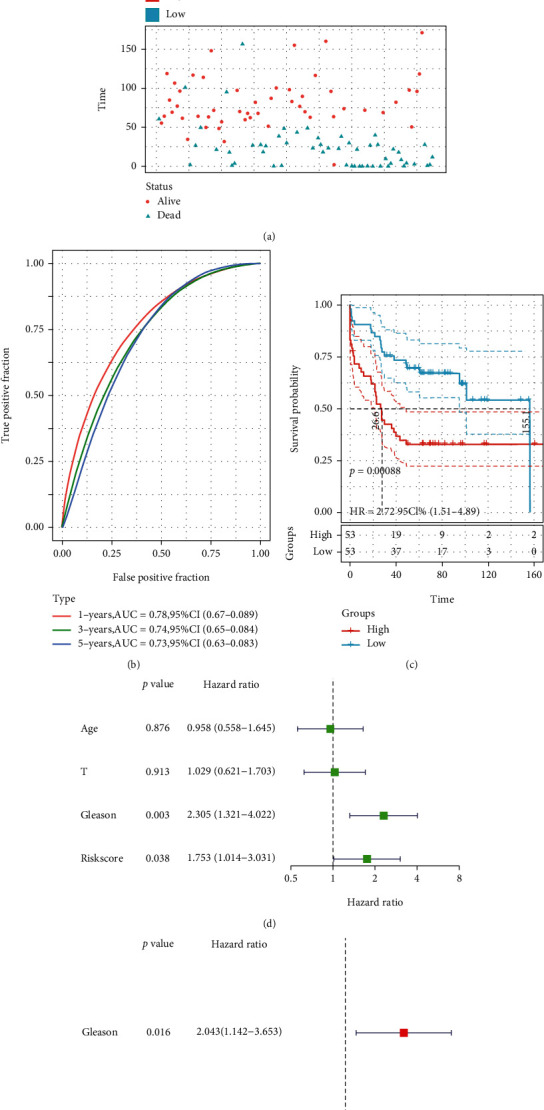
The validation of prognostic signature in GEO cohort. (a) The curve of risk score and BCR status of the patients. (b) ROC curve of model and clinical characteristics predicting 1-, 3-, and 5-year bRFS. (c) The bRFS analysis of the two subgroups stratified based on the median of risk scores calculated by the risk model. (d, e) Univariate and multivariate Cox regression analyses showed that the risk score had prominent prognostic values.

Based on the above computation, patients in the TCGA-PCa cohort were classified into two according to the median risk score, namely, high- or low-risk groups. According to the results, the proportion of patients with BCR in the high-risk group exceeded that in the low-risk group (Figure 5(a)). Patients in the high-risk group had a greater probability of BCR (*P* value < 0.0001) and poorer bRFS (Figure 5(c)). For bRFS prediction, the AUC values for the developed gene signature were used, which were 0.730, 0.760, and 0.750 for 1, 3, and 5 years correspondingly (Figure 5(b)). We checked for other independent predictors among the clinical factors, including Gleason's score (GS), age, and TNM stage by univariate and multivariate Cox regression analyses. When all the clinical features were combined, risk score, GS, and T stage were determined as independent factors associated with bRFS in both univariate and multivariate analyses (Figures 5(d) and 5(e) and Supplementary Table 5) (*P* value < 0.05). The GEO cohort was utilized to validate the predictive robustness of the model. To circumvent information bias-related errors, the same statistical approaches were employed. The results for the validation set were in high agreement with those of TCGA cohort. The BCR rate was higher in the high-risk group than in the low-risk group (Figure 6(a)). Based on survival analysis, poor bRFS in PCa patients may be associated with high-risk scores (Figure 6(c)). The AUC values for 1-, 3-, and 5-year survival were 0.780, 0.740, and 0.730, correspondingly (Figure 6(b)). Finally, risk score and GS remained independent prognostic factors in the GEO cohort (Figures 6(d) and 6(e)).

### 3.3. Correlation of Prognostic Risk Signature with Signature Components

**Figure 7:**
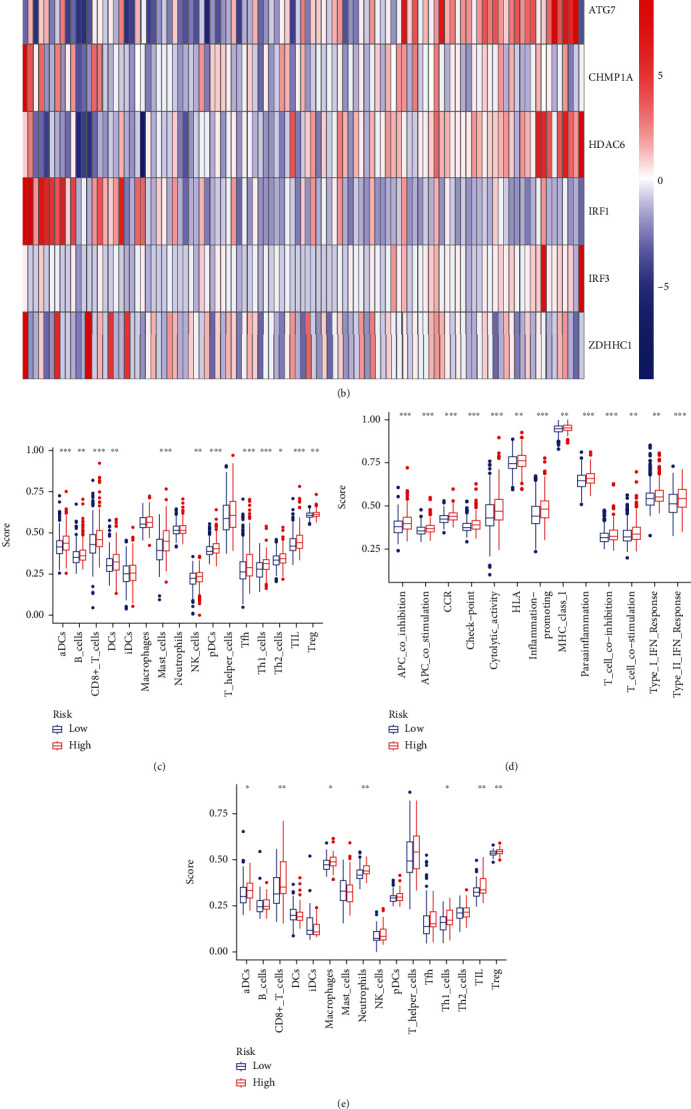
The relationship between the expression of six genes and the risk score in two cohorts; the comparison of immune cells and immune function by single-sample GSEA (ssGSEA) in the two cohorts. (a) The relationship between the expression of six genes and the risk score in TCGA. (b) The relationship between the expression of six genes and the risk score in GEO (^∗^*P* value < 0.05; ^∗∗^*P* value < 0.01; ^∗∗∗^*P* value < 0.001). (c, d) Enrichment results of immune cell score and immune function score in TCGA cohort. (e, f) Enrichment results of immune cell score and immune function score in GEO cohort (^∗^*P* value < 0.05; ^∗∗^*P* value < 0.01; ^∗∗∗^*P* value < 0.001).

A correlation was observed between risk score and signature components. Heatmap showing gene expression exhibited that the risk factors, ATG7, HDAC6, and IRF3, were highly expressed in the high-risk group, while CHMP1A, IRF1, and ZDHHC1, the protective factors, were highly expressed in the low-risk group in both cohorts (Figures 7(a) and 7(b) and Supplementary Table 6).

### 3.4. Immune Infiltration

Previous studies suggest that pyroptosis may be related to immune cells, functions, and microenvironment. Thus, its relationship with 16 immune cell types and activity in 13 immune-related pathways were assessed between the risk groups. The high-risk group had higher scores for most immune cells and functions than the low-risk group, particularly aDCs, Th1_cells, CD8+_T_cells, Treg, TIL, CCR, inflammation promoting, APC_co_stimulation, parainflammation, MHC_class_I, type_II_IFN_response, and T_cell_coinhibition (Figures 7(c)–7(f) and Supplementary Table 7).

### 3.5. ZDHHC1 Inhibits Migration, Invasion, and Proliferation of PCa Cells In Vitro

**Figure 8:**
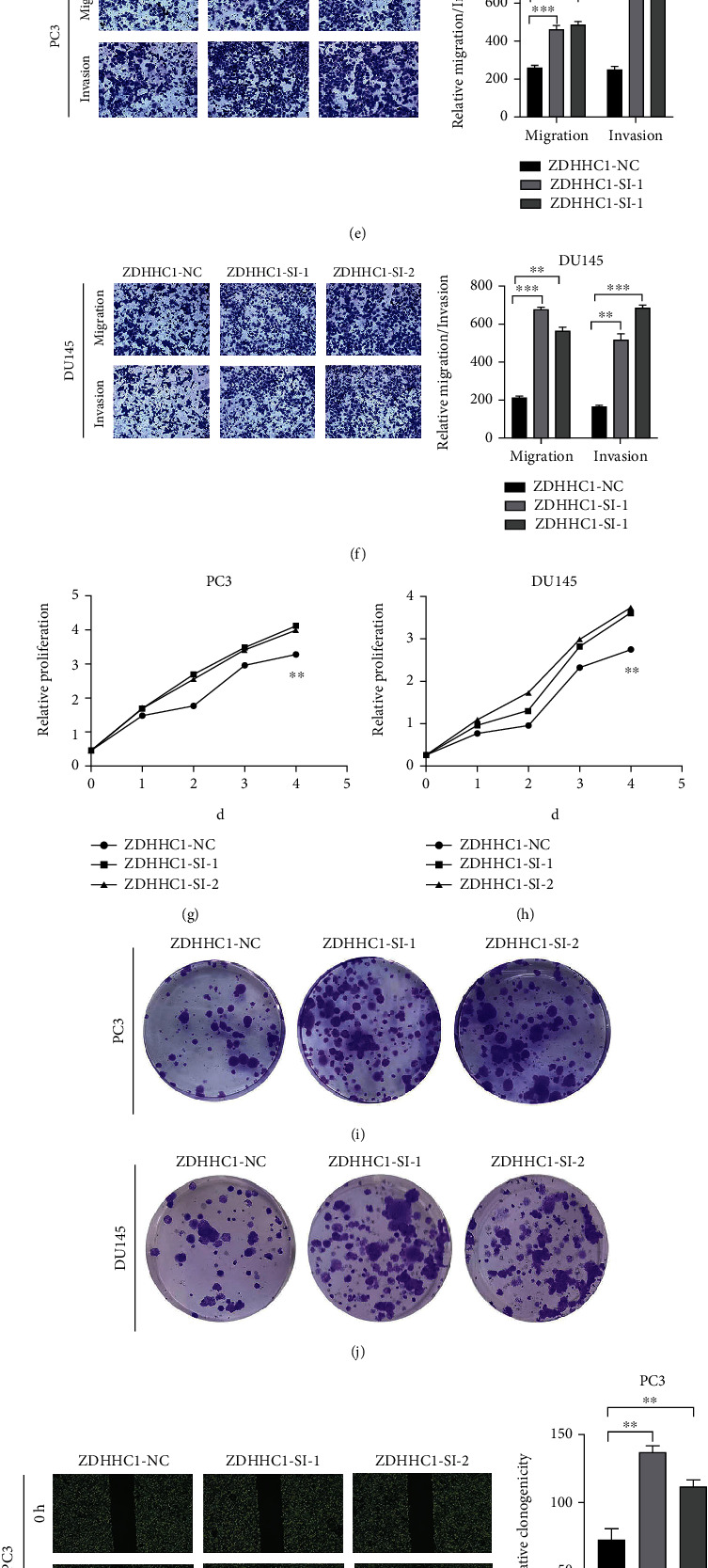
Downregulated ZDHHC1 promotes cell proliferation, migration, and invasion. (a, b) qRT-PCR and western blot analysis indicated that ZDHHC1 was differentially expressed in RWPE-1 and PCa cells. (c, d) qRT-PCR analysis of PC3 and DU145 cells after transfection of ZDHHC1-siRNA or siRNA-NC. (e, f) The influence on cell migration and invasion abilities of transfected cells was assessed by Transwell migration and Matrigel invasion assays. (g–j) Cell proliferation ability of transfected cells was evaluated by CCK8 assay and colony formation assay. (k, l) Cell migration capability of transfected cells was evaluated by wound healing assays.

The coefficient for the ZDHHC1 gene was relatively high in the signature, suggesting its greater influence on prognosis. Based on previous relevant studies, some reports suggest the role of ZDHHC1 in cancer, but for PCa, the evidence remains scarce. Thus, ZDHHC1 was the focus of our subsequent analyses. Moreover, qRT-PCR and western blotting were performed to investigate differences in mRNA and protein expression of ZDHHC1, respectively, between PCa and normal prostate epithelial cell lines (Figures 8(a) and 8(b)). siRNA constructs were employed for silencing ZDHHC1 expression in PC3 and DU145 cells. Knockout efficiency of transfected cell lines was assessed by qRT-PCR (Figures 8(c) and 8(d)). By Transwell migration and Matrigel invasion assays, knocking down of ZDHHC1 was found to significantly promote PCa cell invasion and migration, respectively (Figures 8(e) and 8(f)). Moreover, CCK8 and colony formation assays verified that ZDHHC1 knockdown increased the proliferative ability of PC3 and DU145 cells (Figures 8(g)–8(j)). ZDHHC1 knockdown also promoted PC3 and DU145 cell migration (Figures 8(k) and 8(l)).

**Figure 9:**
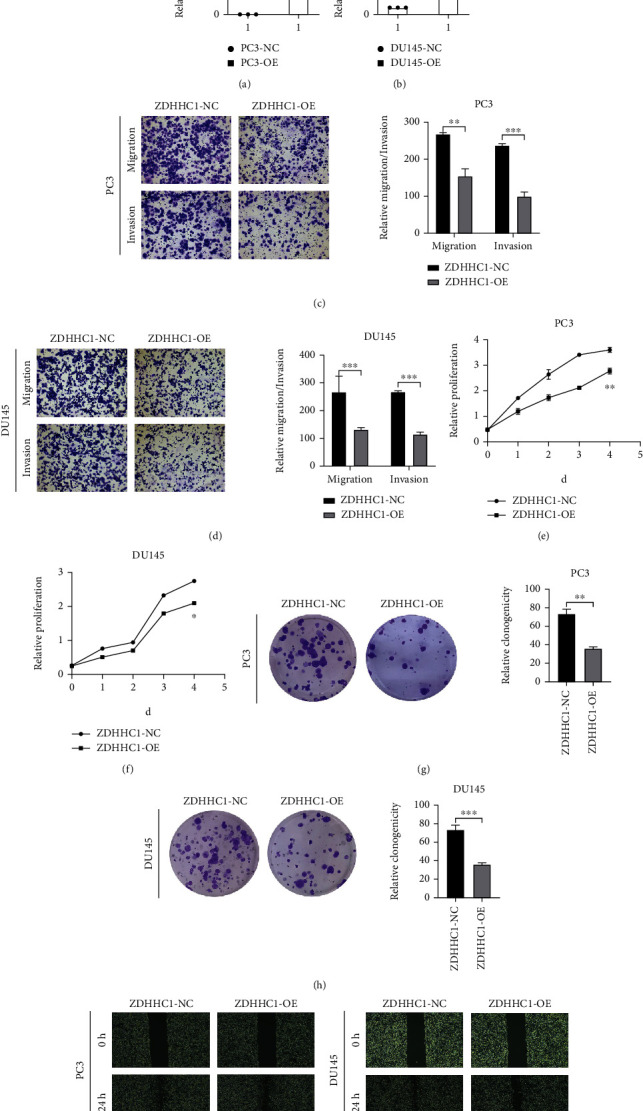
Overexpressed ZDHHC1 suppresses cell proliferation, migration, and invasion. (a, b) QRT-PCR analysis indicated that the efficiency of overexpression of ZDHHC1 in PC3 and DU145 cells. (c, d) The influence on cell migration and invasion abilities of overexpressed cells was assessed by Transwell migration and Matrigel invasion assays. (e–h) Cell proliferation ability of overexpressed cells was evaluated by CCK8 assay and colony formation assay. (i, j) Cell migration capability of overexpressed cells was evaluated by wound healing assays.

By qRT-PCR, stable overexpression of ZDHHC1 in PC3 and DU145 cells was validated (Figures 9(a) and 9(b)). Overexpression of ZDHHC1 inhibited cell migration and invasion (Figures 9(c) and 9(d)) and weakened cell proliferation (Figures 9(e)–9(h)). The migration of PC3 and DU145 cells (Figures 9(i) and 9(j)) was suppressed.

Together, the above in vitro results indicated that ZDHHC1, a tumor suppressor, could suppress PCa cell invasion, migration, and proliferation.

### 3.6. ZDHHC1 Promotes Pyroptosis In Vitro and Inhibits Proliferation In Vivo

**Figure 10:**
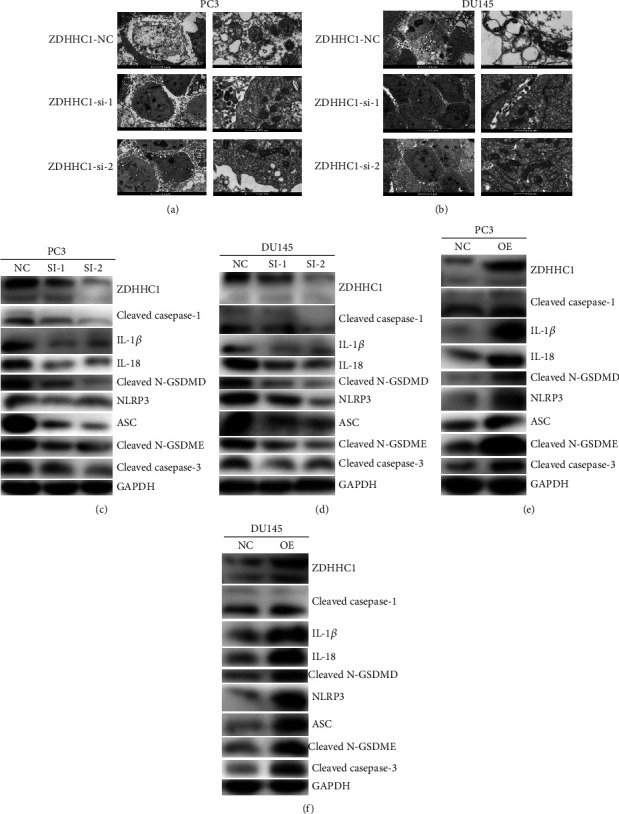
Overexpression of ZDHHC1 promotes pyroptosis. (a, b) Compared with ZDHHC1-si, PC3 and DU145 cells treated with ZDHHC1-NC exhibited cell swelling, decreased plasma membrane integrity, and swollen mitochondrial morphology. (c–f) Changes in the expression of ZDHHC1 affect the expression of important proteins during pyroptosis.

For assessing the effects of ZDHHC1 on PCa pyroptosis regulation, human PC3 and DU145 cells were transfected with control siRNA or target gene siRNA and stable overexpressing ZDHHC1 cell lines were constructed. The typical characteristics of pyroptosis under transmission electron microscopy include reduced membrane integrity, cell swelling and lysis, and mitochondrial swelling. Transmission electron microscopy was performed to evaluate the impact of ZDHHC1 on the plasma membrane and mitochondrial morphology. As shown in Figures 10(a) and 10(b), as compared to ZDHHC1-siRNA-treated PC3 and DU145 cells, ZDHHC1-NC-treated cells showed cell swelling, reduced plasma membrane integrity, and mitochondrial swelling.

ZDHHC1 thus promotes pyroptosis in PCa cell lines. Caspase-1 activation is the core of the classical pyroptosis pathway. In the classical pyroptosis pathway, inflammasome formation activates the caspase-1 precursor. While caspase-1 activation promotes the cleavage of inactive IL-18 and IL-1*β* precursors, resulting in mature IL-18 and IL-1*β*, activated caspase-1 also acts on gastrin D (GSDMD), which is cleaved into reactive carboxyl (C) and amino (N) termini. The N-terminal domain is lipid selective, causing cell lytic death. Simultaneously, small molecules including IL-18 and IL-1*β* are secreted from cell pores. Several immune cell types are recruited, thus triggering an inflammatory response eventually leading to pyroptosis. The nonclassical pyroptosis pathway produces an effect similar to that of caspase-1, leading to membrane perforation which is affected in the presence of NLRP3 and ASC. Caspase-3 has multiple mechanisms of activation. Since a natural caspase-3 cleavage site is present in the C- and N-termini of GSDME, activated caspase-3 can cleave specific sites on GSDME, release the N-terminal active domain, and penetrate the plasma membrane, thereby inducing pyroptosis. Therefore, we sought to examine whether alteration of ZDHHC1 expression affected the above key proteins in the pyroptosis pathway. Our findings suggested a substantial involvement of ZDHHC1 in promoting pyroptosis (Figures 10(c)–10(f)).

**Figure 11:**
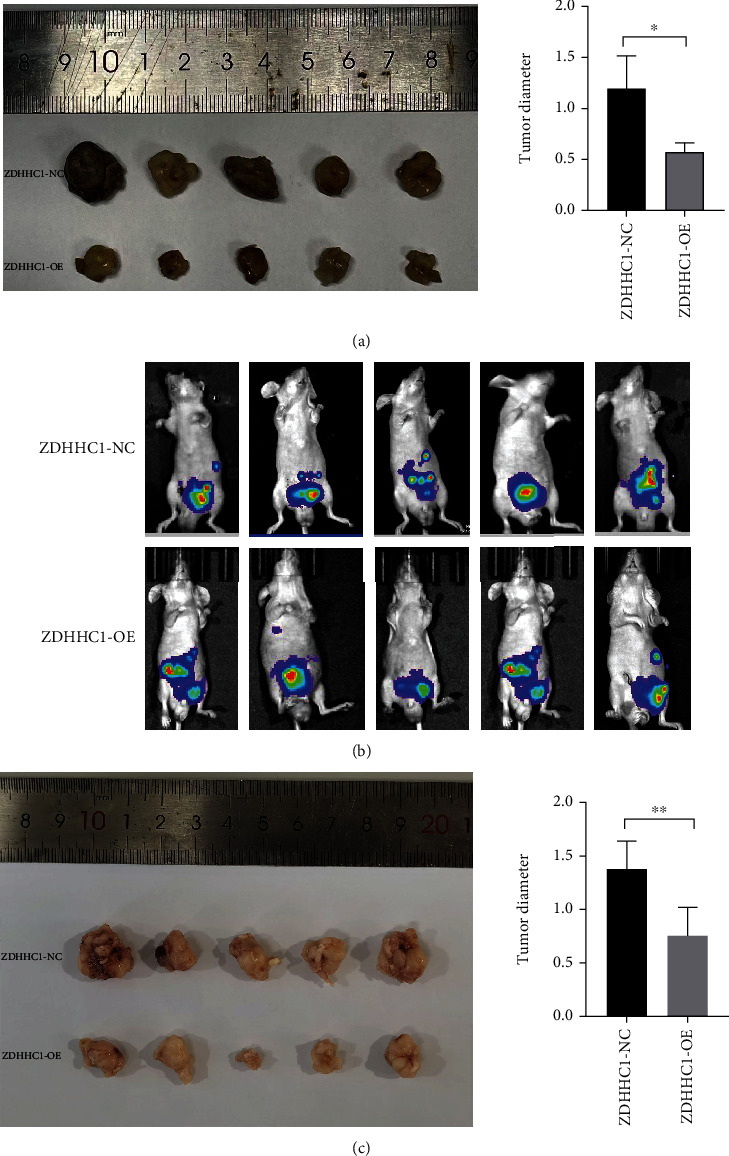
Overexpression of ZDHHC1 suppresses proliferation in vivo. (a) Xenograft tumors in nude mouse models. (b, c) Orthotopically xenograft tumors in nude mouse models.

Finally, in vivo assays were performed to further evaluate the effects of ZDHHC1 on the occurrence and development of PCa in vivo. DU145 cells stably expressing ZDHHC1-NC or ZDHHC1-OE constructs were subcutaneously injected into the BALB/c nude mice. The tumor growth was assessed for four weeks. Weight and volume of tumors reduced remarkably in the ZDHHC1 overexpression group relative to those in the control (Figure 11(a)). We orthotopically xenografted DU145 cells (carrying luciferase) into anterior prostates in the two groups of nude mice, (1) DU145-Luc+ZDHHC1-OE or (2) DU145-Luc+ZDHHC1-NC, to assess the impact of ZDHHC1 on prostate tumorigenesis. Tumor formation was observed by IVIS after four weeks (Figure 11(b)), and a postmortem tumor assessment was performed. The volume and weight of tumors in the ZDHHC1 overexpression group were significantly lower relative to those in the control as shown in Figure 11(c). Thus, ZDHHC1 overexpression suppressed PCa cell tumorigenicity.

## 4. Discussion

PCa, common cancer among men, is the second-leading cause of death among men globally, especially in developed countries [18, 19]. Therapeutic strategies have been developed tremendously over the past decade but remain unsatisfactory for PCa. BCR refers to enhanced PSA concentration > 0.2 *μ*g/L, validated by two consecutive observations. BCR promotes the development of advanced CRPC, resulting in an increased risk of distant metastasis, PCa-specific mortality, and overall mortality, and thus is a determining risk factor [20, 21]. Thus, for frequent monitoring, early intervention, and decision making for adjuvant therapy for those at high risk, classifying patients with post-RP localized PCa into risk groups is favored. PSA is a widely accepted detection parameter for PCa since the 1990s and is a major prognostic factor. More than half of the high-risk PCa patients experience BCR postoperatively [22]. However, due to the poor predictive ability of reported individual biomarkers, identifying new BCR prognostic biomarkers or predictive signatures for localized PCa after RP is crucial.

Pyroptosis, associated with immune and inflammatory responses and mediated by the gasdermin family, is a recently uncovered type of programmed cell death. It not only facilitates a microenvironment for nutrition for cancer cells, thereby accelerating growth, but also inhibits the development and occurrence of tumors [23]. The early stage of pyroptosis involves apoptotic processes, and the downstream pathway of pyroptosis activation by injury or infection has been detailed. Recently, pyroptosis has gained attention, owing to its putative benefits for anticancer therapies. The processes of pyroptosis activation involve the cellular content release causing toxicity to adjacent healthy cells and inducing an inflammatory response, ultimately leading to cell death. Ruan et al. [24] reported that the mechanisms underlying nonapoptotic programmed cell death, like pyroptosis, may be effective for rechallenging apoptosis-resistant cancer cells. Several studies confirm that pyroptosis is tightly associated with the development and occurrence of several disorders, including cancer [25–28]. Specifically, Wang et al. [29] have identified new mechanisms in cancer cell pyroptosis that may lead to the discovery of new drug targets. Owing to the important role of GSDMD/GSDME in the regulation of both pyroptosis and sensitivity to cancer therapy, a new role for pyroptosis may be implicated in the future. However, the link between pyroptosis and PCa is elusive. In cancer, pyroptosis acts as a double-edged sword. The function of PRGs in PCa remains unclear. The most direct way to elucidate the importance of pyroptosis in PCa was by establishing a prognostic model and studying its components.

We aimed to identify a signature based on the genetic markers of pyroptosis for predicting BCR in PCa. We also performed a basic experimental validation of the important components in the model. For large-scale omics research, high-throughput gene sequencing of biological samples is feasible. First, by univariate Cox regression analysis, 34 PRGs correlated with BCR in PCa were screened according to the differences between normal and tumor tissues. The results for PPI and gene expression correlational network uncovered remarkable functional and expressional relations among the genes. A LASSO regression analysis was conducted to eventually construct a six-PRG prognostic signature. bRFS analysis of this signature proved the excellent predictive ability for BCR in PCa. Additionally, the calculated risk scores and GS were independent risk factors for PCa-BCR. Risk scores and poor clinicopathological characteristics, including GS, and T staging correlated positively. Generally, higher risk scores indicated a higher level of immune cell infiltrates with higher activity of immune-associated functions. In particular, the increase in cell types such as CD8+_T_cells and enhanced type_II_IFN_response, inflammation-promoting effects, and other processes all indicated improved immunity, thus confirming that the cancer was in late stages with a worse patient prognosis. Notably, all of these findings were verified in two independent PCa cohorts (TCGA and GEO).

Based on the analysis of the six-gene signature, as an important predictor, ZDHHC1, which has been implicated previously in other cancers but rarely studied in PCa, was found to account for substantially high weight. It may play a significant role in the evaluation of patients' risk scores. Tang et al. [30] report that the protein product of ZDHHC1 is an S-palmitoyltransferase; p53 is a substrate for ZDHHC1-mediated palmitoylation. A novel type of posttranslational modification of p53 is necessary for the nuclear translocation of the tumor suppressor. Kisiel et al. [31] show the association of ZDHHC1 promoter methylation with inflammatory bowel disease-associated neoplasia. Moreover, ZDHHC1, associated with pyroptosis, is a frequently silenced potential tumor suppressor by promoter methylation. It can negatively regulate tumor cell metabolism while stimulating ER and oxidative stress to accelerate cell death through pyroptosis and apoptosis induction. It can thus be exploited for developing novel cancer therapies and prevention strategies [32]. In our experiment, ZDHHC1 was validated to activate caspase-1 in the classical pyroptosis pathway and caspase-3 in the nonclassical pyroptosis pathway. It further caused an increase in GSDMD-N, IL-18, and IL-1*β* in the classical pyroptosis pathway and GSDME-N, NLRP3, and ASC in the nonclassical pyroptosis pathway. These expressions were reduced after inhibition of ZDHHC1 expression. This showed that ZDHHC1 promotes the formation of pyroptosis in vitro experiments through both pathways. The effects of ZDHHC1 on the tumorigenesis and development in PCa cell lines, as well as on the process of pyroptosis, were assessed. ZDHHC1 was also critically related to immune responses. ZDHHC1, also known as ZNF377, is relatively understudied. Reports on its cellular and biological roles are scarce. It has been implicated in promoting immune responses to viral infections [33]. Sowers et al. [34] have identified the key BPA upregulation of ZDHHC1 protein and show its implication for the activation of innate viral immune pathways and type 1 interferon responses. Zhou et al. [33] report the role of ZDHHC1 in mediating MITA/STING-dependent innate immune responses against DNA viruses. In our results, CD8+_T_cell, type_II_IFN_response, and inflammation-promoting processes were associated with the high-risk group, while the enhanced immune cell abundance and processes suggested that these were necessary to maintain homeostasis. This further validated the reliability of the signature for evaluating patients' prognoses.

Apart from ZDHHC1, the remaining five genes in the signature were also related to pyroptosis and cancer. ATG7 plays an important role in tumor resistance. Zhang et al. [35] confirm that ATG7-independent alternative autophagy promotes tumor survival, unlike ATG7-dependent selective autophagy. Delayed Pten-deficient prostate tumor progression in both castrate-resistant and castrate-naïve cancers and autophagy-deficient phenotype is attributed to Atg7 deficiency. Atg7-deficient tumors show ER stress, hinting that autophagy promotes PCa tumorigenesis by regulating protein homeostasis [36]. Atg7 loss results in increased production of IL-1*β* and pyroptosis, consistent with enhanced inflammasome activation [37]. The antitumor function of CHMP1A, especially in the pancreas, has been verified in vivo and in vitro [38]. Cellular studies indicate that CHMP1A is an important regulator of programmed death through a single pathway and leads to the development of kidney disease by altered cellular iron trafficking. HDAC6 is a member of the HDAC family, which has a crucial function in multiple disorders, the most prominent being gastrointestinal cancer. Typically, it is an oncogenic factor in colorectal, pancreatic, and esophageal cancers [39]. Yang et al. [40] verify that HDAC6, having biological functions owing to a specific structure, exerts substantial influence on the progression, metastasis, and carcinogenesis of tumors. HDAC inhibitors may open up new therapeutic avenues for cancers and neurological disorders. HDAC6 is a potential therapeutic target for inflammasome-centric diseases [41]; its role in nicotine-induced macrophage pyroptosis has been previously demonstrated [42]. IRF1 downregulates the RAS-RAC1 pathway by promoting the expression of RASSF5 and inhibits metastasis and proliferation of colorectal cancer cells [43]. IRF1 is also a potential tumor suppressor in non-small-cell lung cancer [44]. Meyer-Schaller et al. [45] suggest that the context-dependent dual function of IRF1 in regulating epithelial-mesenchymal plasticity gives crucial novel insights into the therapeutic potential and functional roles of interferon-regulated factors in breast cancer. Recent findings hint at a potential mechanism of IRF1 targeting macrophage pyroptosis and inflammation in ACS and AS by promoting m6A modifications. Through comprehensive bioinformatics, Wu et al. [46] confirm IRF3 as a putative prognostic biomarker and therapeutic target for ccRCC. IRF3 is a key YAP activator, implying that its pharmacological targeting with a small compound inhibitor can elicit broadened antitumor effects against YAP-driven human cancers [47]. Moreover, IRF3 knockdown inhibits NLRP3 mediated pyroptosis and weakens LPS-induced cardiac injury.

Our study has some limitations. First, the retrospective analysis was performed using public datasets. Further validation is required in prospective cohorts. Second, because of the innate heterogeneity of tumors and technical noise related to cross-platform sequencing, rational regulation of expression data remains challenging for the application of the six-gene signature. Finally, due to the simplicity of the validation set, the accuracy of the model necessitates further verification. Therefore, in future basic and clinical applications, data preprocessing, including scaling and normalization, is required for patients' RNA-seq data. Developing a standardized and commercial genetic test kit with the six genes to automatically calculate the risk score is useful. Collectively, the potential biological mechanism for this feature, especially how ZDHHC1 affects pyroptosis in PCa, along with the pathological mechanism needs to be elucidated.

## 5. Conclusions

In summary, we constructed a six-gene signature related to pyroptosis that could precisely predict BCR in PCa. Higher risk scores suggested a higher probability of BCR and worsened clinicopathological features. The signature, as a new tool to distinguish between PCa patients with differential risks, helps understand the mechanism for cellular pyroptosis in the carcinogenesis and development of PCa. Finally, the effects of ZDHHC1, accounting for a substantial weight in the signature, on tumorigenesis, development, and the process of cell pyroptosis, were assessed. ZDHHC1 exerted inhibitory effects on tumor migration, invasion, and proliferation. The mechanistic basis of these effects warrants further research.

## Data Availability

The datasets analyzed during the current study are available in the TCGA (https://portal.gdc.cancer.gov/) and GEO repository (https://www.ncbi.nlm.nih.gov/geo/). The data and materials can be obtained by contacting the corresponding author.

## Abbreviations

PCa: Prostate cancer
BCR: Biochemical recurrence
TCGA: The Cancer Genome Atlas
GEO: Gene Expression Omnibus
RP: Radical prostatectomy
PSA: Prostate-specific antigen
PRG: Pyroptosis-associated gene
DEG: Differentially expressed gene
FDR: False discovery rate
bRFS: Biochemical recurrence-free survival
ROC: Receiver operating characteristic
AUC: Area under the ROC curve
qRT-PCR: Quantitative RT-PCR
HR: Hazard ratio
OD: Optical density
PPI: Protein-protein interaction
DFS: Disease-free survival.

## Supplementary Materials

Supplementary 1 Supplementary Table 1: the 203 pyroptosis-associated genes.

Supplementary 2 Supplementary Table 2: primers and RNA sequences used in this study.

Supplementary 3 Supplementary Table 3: the 124 differentially expressed genes were identified in tumor tissue relative to normal tissue in the TCGA cohort.

Supplementary 4 Supplementary Table 4: the 34 pyroptosis-associated genes for constructing prognostic models.

Supplementary 5 Supplementary Table 5: risk grouping in the construction of prognostic signatures.

Supplementary 6 Supplementary Table 6: associations between prognostic risk signature and clinical features.

Supplementary 7 Supplementary Table 7: associations between risk groups with 16 immune cell types and activity in 13 immune-related pathways.

Supplementary 8 Supplementary Figure 1: potential prognostic PRGs from TCGA cohort in cluster 1/2 subgroups. (a) Consensus clustering matrix for *k* = 2. (b) Heatmap generated from the consistency matrix. (c) The cumulative density function (CDF) is used to measure the distribution of values in the consistency matrix. (d) As the number of clusters increases, the relative change in the area under the curve of the CDF. (e) In the case of different *k*, the class to which the sample belongs changes. (f) Heatmap of cluster 1/2 subgroups.

Supplementary 9 Supplementary Figure 2: a six-mRNA signature was constructed by LASSO Cox regression. (a, b) Signature was constructed by LASSO Cox regression, and learn that the combination formed by 6 genes out of a random combination of 33 differential genes was optional.
