# Supplementary material for: Identification of ZDHHC1 as a Pyroptosis Inducer and Potential Target in the Establishment of Pyroptosis-Related Signature in Localized Prostate Cancer
Source: Oxid Med Cell Longev. 2022 Dec 22;2022:5925817. doi: 10.1155/2022/5925817 (PMC9800907; doi:10.1155/2022/5925817)
Supplement: Supplementary 1 — Supplementary Table 1: the 203 pyroptosis-associated genes. [file 5925817.f1.docx]

| ABL1(GeneCards) | BECN1(GeneCards) | CASP9(GeneCards) | DPP9(GeneCards) |
| --- | --- | --- | --- |
| ACE2(GeneCards) | BHLHE40(GeneCards) | CD14(GeneCards) | DRD2(GeneCards) |
| ADORA1(GeneCards) | BHLHE41(GeneCards) | CD274(GeneCards) | DUOX1(GeneCards) |
| ADORA2A(GeneCards) | BIRC2(GeneCards) | CDC37(GeneCards) | E2F4(GeneCards) |
| ADORA2B(GeneCards) | BIRC3(GeneCards) | CDK9(GeneCards) | EEF2K(GeneCards) |
| ADORA3(GeneCards) | BNIP3(GeneCards) | CEBPB(GeneCards) | EGFR(GeneCards) |
| AGER(GeneCards) | BRCC3(GeneCards) | CGAS(GeneCards) | ELANE(Reactome) |
| AIM2(GeneCards) | BRD4(GeneCards) | CHI3L1(GeneCards) | ELAVL1(GeneCards) |
| AKT1(GeneCards) | BSG(GeneCards) | CHMP1A(GeneCards) | ERP44(GeneCards) |
| ALK(GeneCards) | BST2(GeneCards) | CHMP4B(GSEA) | FADD(GeneCards) |
| ANO6(GeneCards) | BTK(GeneCards) | CLEC5A(GeneCards) | FGF21(GeneCards) |
| ANXA2(GeneCards) | CAMP(GeneCards) | CPTP(GeneCards) | FNDC4(GeneCards) |
| APIP(GeneCards) | CAPN1(GeneCards) | CRTAC1(GeneCards) | FNDC5(GeneCards) |
| APOE(GeneCards) | CARD8(GeneCards) | CTSG(GeneCards) | FOXO3(GeneCards) |
| APOL1(GeneCards) | CASP1(Reactome) | CTSV(GeneCards) | FOXP3(GeneCards) |
| ASIC1(GeneCards) | CASP3(Reactome) | CXCL8(GeneCards) | GBP1(GeneCards) |
| ATF6(GeneCards) | CASP4(Reactome) | DDX3X(GeneCards) | GBP5(GeneCards) |
| ATG3(GeneCards) | CASP5(Reactome) | DHX9(GeneCards) | GJA1(GeneCards) |
| ATG7(GeneCards) | CASP6(GeneCards) | DPEP1(GeneCards) | GLMN(GeneCards) |
| BCL2(GeneCards) | CASP8(GeneCards) | DPP8(GeneCards) | GPER1(GeneCards) |

| GSDMA(GeneCards) | IL1RN(GeneCards) | MRE11(GeneCards) | NR1H2(GeneCards) |
| --- | --- | --- | --- |
| GSDMB(GeneCards) | IL32(GeneCards) | MST1(GeneCards) | ORMDL3(GeneCards) |
| GSDMC(GeneCards) | IL36B(GeneCards) | MYD88(GeneCards) | P2RX7(GeneCards) |
| GSDMD(Reactome) | IL36G(GeneCards) | NAIP(GeneCards) | PANX1(GeneCards) |
| GSDME(Reactome) | IRAK3(GeneCards) | NCR1(GeneCards) | PARP1(GeneCards) |
| GSTO1(GeneCards) | IRF1(Reactome) | NEDD4(GeneCards) | PCSK9(GeneCards) |
| GZMA(GeneCards) | IRF2(Reactome) | NEK7(GeneCards) | PDCD6IP(GeneCards) |
| GZMB(Reactome) | IRF3(GeneCards) | NFE2L2(GeneCards) | PECAM1(GeneCards) |
| HDAC6(GeneCards) | IRGM(GeneCards) | NFKB1(GeneCards) | PGF(GeneCards) |
| HMGB1(Reactome) | JUN(GeneCards) | NINJ1(GeneCards) | POP1(GeneCards) |
| HNP1(GeneCards) | LRPPRC(GeneCards) | NLRC4(GeneCards) | PRDM1(GeneCards) |
| HSP90AA1(GeneCards) | LY96(GeneCards) | NLRP1(GeneCards) | PRF1(GeneCards) |
| HSP90AB1(GeneCards) | LYST(GeneCards) | NLRP13(GeneCards) | PRKN(GeneCards) |
| HUWE1(GeneCards) | MALT1(GeneCards) | NLRP3(GeneCards) | PRTN3(GeneCards) |
| IFI16(GeneCards) | MDM2(GeneCards) | NLRP6(GeneCards) | PTEN(GeneCards) |
| IKBKE(GeneCards) | MEFV(GeneCards) | NLRP7(GeneCards) | PTGS2(GeneCards) |
| IL13(GeneCards) | MELK(GeneCards) | NLRP9(GeneCards) | PYCARD(GeneCards) |
| IL13RA2(GeneCards) | METTL3(GeneCards) | NLRX1(GeneCards) | PYDC2(GeneCards) |
| IL18(GSEA) | MKI67(GeneCards) | NOS1(GeneCards) | RAB5A(GeneCards) |
| IL1B(GSEA) | MLKL(GeneCards) | NOS2(GeneCards) | RIPK1(GeneCards) |

RIPK3(GeneCards) TLR9(GeneCards) YWHAZ(GeneCards) SCAF11(GeneCards) TNF(GeneCards) ZBP1(GeneCards) SDHB(GeneCards) TNFSF13B(GeneCards) ZDHHC1(GeneCards) SEC22B(GeneCards) TP53(Reactome)

SERPINB1(GeneCards) TP63(Reactome) SESN2(GeneCards) TREM2(GeneCards)

SIGLEC14(GeneCards) TRIM21(GeneCards) SIRT1(GeneCards) TRIM31(GeneCards) SLC16A4(GeneCards) TRPM2(GeneCards) SQSTM1(GeneCards) TUBB6(GeneCards) STAT3(GeneCards) TXNIP(GeneCards) STING1(GeneCards) UBE2D2(GeneCards) STK4(GeneCards) UBE2D3(GeneCards)

STXBP2(GeneCards) UBR2(GeneCards) STXBP3(GeneCards) UTS2(GeneCards) TET2(GeneCards) VDR(GeneCards) TFAM(GeneCards) VIM(GeneCards) TFAP2A(GeneCards) VPS28(GeneCards)

TLR2(GeneCards) VPS4B(GeneCards) TLR8(GeneCards) YWHAE(GeneCards)
