# Supplementary material for: Identification of ZDHHC1 as a Pyroptosis Inducer and Potential Target in the Establishment of Pyroptosis-Related Signature in Localized Prostate Cancer
Source: Oxid Med Cell Longev. 2022 Dec 22;2022:5925817. doi: 10.1155/2022/5925817 (PMC9800907; doi:10.1155/2022/5925817)
Supplement: Supplementary 2 — Supplementary Table 2: primers and RNA sequences used in this study. [file 5925817.f2.docx]

Item Sequence (5′-3′) Primers

GAPDH F GGAGCGAGATCCCTCCAAAAT GAPDH R GGCTGTTGTCATACTTCTCATGG

ZDHHC1-F1 CAAGCCCTCCAACAAGACG ZDHHC1-R1 CCAAAGCCGATCACAGCAAAG ZDHHC1-F2 GTGCGGGACAAGAGCTATG ZDHHC1-R2 AGTTGCAGTGCAGGTCTTCAA ZDHHC1-F3 CAACTTGTGCAACGTGGATGT ZDHHC1-R3 AAGAGCCGGTAGTTCCGCT

siRNAs

si-ZDHHC1-1 GCACGCACATGTCATTGAA

si-ZDHHC1-2 GAGTCATGTCCTCCCAAGA
