# Supplementary material for: Identification of ZDHHC1 as a Pyroptosis Inducer and Potential Target in the Establishment of Pyroptosis-Related Signature in Localized Prostate Cancer
Source: Oxid Med Cell Longev. 2022 Dec 22;2022:5925817. doi: 10.1155/2022/5925817 (PMC9800907; doi:10.1155/2022/5925817)
Supplement: Supplementary 3 — Supplementary Table 3: the 124 differentially expressed genes were identified in tumor tissue relative to normal tissue in the TCGA cohort. [file 5925817.f3.docx]

| Gene | Conmean | Treatmean | Log_2_(fold change) | P value |
| --- | --- | --- | --- | --- |
| ACE2 | 1.787 | 0.180 | -3.315 | 2.2E-10 |
| ADORA1 | 0.144 | 0.173 | 0.266 | 1.3E-02 |
| ADORA2A | 0.031 | 0.048 | 0.626 | 2.9E-04 |
| ADORA2B | 1.478 | 0.720 | -1.037 | 2.9E-07 |
| AGER | 1.366 | 1.787 | 0.388 | 2.1E-02 |
| AKT1 | 17.205 | 19.621 | 0.190 | 1.2E-03 |
| ANO6 | 14.611 | 5.996 | -1.285 | 9.3E-21 |
| ANXA2 | 50.233 | 25.778 | -0.962 | 2.3E-19 |
| APOE | 16.745 | 52.344 | 1.644 | 7.0E-14 |
| APOL1 | 28.277 | 19.379 | -0.545 | 3.0E-07 |
| ASIC1 | 1.491 | 2.145 | 0.524 | 6.0E-06 |
| ATF6 | 10.636 | 8.698 | -0.290 | 8.3E-07 |
| BCL2 | 6.486 | 3.246 | -0.999 | 2.2E-15 |
| BHLHE40 | 63.396 | 42.280 | -0.584 | 3.1E-04 |
| BIRC3 | 4.470 | 3.791 | -0.238 | 9.9E-03 |
| BNIP3 | 17.578 | 22.742 | 0.372 | 5.9E-05 |
| BRCC3 | 8.468 | 9.729 | 0.200 | 3.4E-04 |
| BSG | 137.278 | 177.128 | 0.368 | 4.7E-08 |
| BST2 | 50.613 | 45.828 | -0.143 | 2.3E-06 |
| CAMP | 2.348 | 0.239 | -3.298 | 2.0E-05 |
| CAPN1 | 32.169 | 26.271 | -0.292 | 2.9E-08 |
| CASP1 | 3.213 | 1.936 | -0.731 | 2.5E-09 |
| CASP5 | 0.030 | 0.027 | -0.151 | 3.7E-02 |
| CASP6 | 7.498 | 8.179 | 0.126 | 3.8E-02 |
| CASP8 | 2.225 | 2.796 | 0.329 | 6.8E-05 |
| CD274 | 0.731 | 0.493 | -0.569 | 1.5E-08 |
| CDC37 | 45.362 | 55.323 | 0.286 | 5.3E-09 |
| CDK9 | 17.460 | 19.992 | 0.195 | 3.4E-04 |
| CEBPB | 22.698 | 17.215 | -0.399 | 9.5E-05 |
| CGAS | 1.350 | 1.013 | -0.414 | 4.7E-05 |
| CHI3L1 | 0.900 | 2.273 | 1.336 | 3.6E-07 |
| CPTP | 12.771 | 16.446 | 0.365 | 2.1E-07 |
| CRTAC1 | 10.011 | 0.652 | -3.940 | 1.5E-19 |
| CTSG | 1.244 | 0.820 | -0.601 | 2.9E-02 |
| DPP8 | 5.103 | 3.896 | -0.389 | 1.9E-09 |
| DRD2 | 0.379 | 0.126 | -1.588 | 1.5E-11 |
| DUOX1 | 6.018 | 1.524 | -1.981 | 1.4E-19 |
| E2F4 | 13.879 | 13.160 | -0.077 | 2.2E-02 |
| EEF2K | 13.821 | 10.158 | -0.444 | 6.6E-10 |
| EGFR | 12.637 | 9.000 | -0.490 | 1.3E-07 |
| ELANE | 0.507 | 0.278 | -0.869 | 8.2E-03 |
| ELAVL1 | 11.524 | 13.378 | 0.215 | 7.8E-09 |
| ERP44 | 18.634 | 22.504 | 0.272 | 4.2E-06 |
| FADD | 3.589 | 3.948 | 0.137 | 3.9E-02 |
| FNDC4 | 8.588 | 6.015 | -0.514 | 3.6E-04 |
| FNDC5 | 1.531 | 1.006 | -0.605 | 1.1E-04 |
| FOXP3 | 0.694 | 0.924 | 0.412 | 3.4E-04 |
| GBP1 | 6.752 | 4.263 | -0.663 | 5.2E-08 |
| GJA1 | 60.706 | 25.371 | -1.259 | 6.8E-21 |
| GLMN | 1.918 | 2.455 | 0.356 | 2.2E-07 |

| GPER1 | 4.080 | 2.719 | -0.586 | 2.1E-09 |
| --- | --- | --- | --- | --- |
| GSDMA | 0.048 | 0.146 | 1.592 | 1.4E-12 |
| GSDMB | 0.851 | 1.643 | 0.950 | 1.6E-06 |
| GSDMD | 10.635 | 10.581 | -0.007 | 2.1E-02 |
| GSDME | 1.226 | 0.745 | -0.719 | 1.2E-11 |
| HDAC6 | 4.432 | 4.888 | 0.141 | 1.9E-02 |
| HMGB1 | 26.499 | 25.008 | -0.084 | 4.2E-02 |
| HSP90AA1 | 233.590 | 196.800 | -0.247 | 4.0E-02 |
| HSP90AB1 | 441.593 | 526.097 | 0.253 | 1.8E-07 |
| IFI16 | 8.804 | 5.422 | -0.699 | 1.1E-08 |
| IKBKE | 2.237 | 1.646 | -0.442 | 2.2E-06 |
| IL13RA2 | 1.560 | 1.404 | -0.152 | 7.9E-04 |
| IL18 | 2.434 | 1.551 | -0.650 | 8.4E-05 |
| IL1B | 1.121 | 0.811 | -0.467 | 7.7E-03 |
| IL1RN | 2.151 | 0.850 | -1.339 | 1.4E-07 |
| IRAK3 | 1.632 | 0.764 | -1.095 | 2.8E-13 |
| IRF2 | 15.224 | 12.350 | -0.302 | 1.5E-09 |
| IRF3 | 11.109 | 13.821 | 0.315 | 9.7E-04 |
| JUN | 151.554 | 131.276 | -0.207 | 2.1E-02 |
| LYST | 1.515 | 1.046 | -0.535 | 1.6E-07 |
| MALT1 | 6.317 | 8.459 | 0.421 | 9.3E-03 |
| MELK | 0.344 | 1.055 | 1.618 | 1.8E-16 |
| METTL3 | 5.700 | 8.490 | 0.575 | 2.2E-15 |
| MKI67 | 0.560 | 1.611 | 1.525 | 2.2E-17 |
| MST1 | 0.787 | 1.576 | 1.002 | 6.4E-09 |
| NCR1 | 0.039 | 0.029 | -0.431 | 3.9E-03 |
| NEDD4 | 2.166 | 1.522 | -0.509 | 6.4E-07 |
| NEK7 | 13.571 | 8.612 | -0.656 | 6.0E-12 |
| NFE2L2 | 22.899 | 15.138 | -0.597 | 2.9E-18 |
| NFKB1 | 10.592 | 9.488 | -0.159 | 2.8E-02 |
| NINJ1 | 22.666 | 19.237 | -0.237 | 4.1E-05 |
| NLRP1 | 2.514 | 1.524 | -0.722 | 8.5E-08 |
| NLRP13 | 0.008 | 0.066 | 2.977 | 1.8E-11 |
| NLRP7 | 0.038 | 0.028 | -0.470 | 1.3E-03 |
| NLRP9 | 0.499 | 0.247 | -1.014 | 2.2E-07 |
| NOS1 | 0.785 | 0.271 | -1.536 | 9.4E-13 |
| NOS2 | 0.310 | 0.368 | 0.246 | 3.3E-03 |
| NR1H2 | 27.290 | 24.931 | -0.130 | 1.1E-03 |
| ORMDL3 | 28.809 | 33.240 | 0.206 | 3.8E-02 |
| P2RX7 | 0.959 | 0.659 | -0.542 | 2.9E-05 |
| PARP1 | 21.978 | 24.518 | 0.158 | 4.3E-02 |
| PCSK9 | 0.141 | 0.081 | -0.804 | 1.1E-11 |
| PGF | 2.423 | 0.888 | -1.449 | 1.5E-16 |
| POP1 | 1.139 | 1.481 | 0.379 | 3.4E-05 |
| PRKN | 1.286 | 0.841 | -0.613 | 8.8E-12 |
| PTEN | 10.187 | 7.152 | -0.510 | 1.6E-08 |
| PTGS2 | 51.037 | 7.999 | -2.674 | 1.2E-10 |
| PYCARD | 6.782 | 4.632 | -0.550 | 3.8E-09 |
| RIPK3 | 0.941 | 0.524 | -0.846 | 7.6E-09 |
| SDHB | 21.576 | 23.244 | 0.107 | 1.2E-03 |
| SERPINB1 | 13.954 | 6.416 | -1.121 | 9.1E-15 |

| SESN2 | 3.788 | 3.417 | -0.149 | 2.1E-02 |
| --- | --- | --- | --- | --- |
| SLC16A4 | 2.276 | 1.127 | -1.014 | 2.2E-15 |
| SQSTM1 | 37.224 | 30.841 | -0.271 | 9.7E-10 |
| STING1 | 12.876 | 8.768 | -0.554 | 4.9E-09 |
| STK4 | 4.167 | 3.610 | -0.207 | 1.2E-04 |
| STXBP2 | 9.600 | 14.595 | 0.604 | 3.4E-12 |
| STXBP3 | 7.917 | 7.197 | -0.138 | 1.9E-02 |
| TLR2 | 3.009 | 1.297 | -1.215 | 2.0E-08 |
| TNF | 0.278 | 0.238 | -0.224 | 3.4E-02 |
| TP53 | 14.506 | 17.191 | 0.245 | 3.9E-04 |
| TP63 | 11.836 | 3.473 | -1.769 | 1.6E-21 |
| TREM2 | 2.864 | 4.767 | 0.735 | 1.1E-04 |
| TRIM31 | 1.375 | 0.317 | -2.118 | 8.4E-04 |
| TRPM2 | 0.786 | 2.049 | 1.382 | 3.1E-08 |
| TUBB6 | 18.812 | 10.069 | -0.902 | 2.7E-11 |
| TXNIP | 228.404 | 154.205 | -0.567 | 1.7E-08 |
| UBE2D2 | 20.509 | 21.614 | 0.076 | 9.0E-03 |
| VIM | 85.852 | 62.132 | -0.467 | 1.6E-05 |
| VPS28 | 31.842 | 43.183 | 0.440 | 3.7E-07 |
| VPS4B | 14.512 | 12.292 | -0.240 | 1.8E-04 |
| YWHAE | 124.962 | 148.526 | 0.249 | 6.6E-07 |
| ZBP1 | 0.112 | 0.192 | 0.783 | 2.8E-02 |
| ZDHHC1 | 5.781 | 4.893 | -0.241 | 4.4E-04 |
