# Supplementary material for: Identification of ZDHHC1 as a Pyroptosis Inducer and Potential Target in the Establishment of Pyroptosis-Related Signature in Localized Prostate Cancer
Source: Oxid Med Cell Longev. 2022 Dec 22;2022:5925817. doi: 10.1155/2022/5925817 (PMC9800907; doi:10.1155/2022/5925817)
Supplement: Supplementary 4 — Supplementary Table 4: the 34 pyroptosis-associated genes for constructing prognostic models. [file 5925817.f4.docx]

| Gene | HR | HR.95L | HR.95H | Pvalue |
| --- | --- | --- | --- | --- |
| ADORA1 | 3.977 | 1.453 | 10.885 | 7.19E-03 |
| ADORA2A | 27.987 | 3.091 | 253.401 | 3.04E-03 |
| ATG7 | 1.376 | 1.144 | 1.655 | 7.08E-04 |
| BST2 | 1.270 | 1.048 | 1.540 | 1.48E-02 |
| CDC37 | 2.743 | 1.365 | 5.515 | 4.61E-03 |
| CDK9 | 4.176 | 2.392 | 7.290 | 4.98E-07 |
| CHMP1A | 0.648 | 0.421 | 1.000 | 4.99E-02 |
| ELAVL1 | 3.003 | 1.132 | 7.970 | 2.72E-02 |
| FNDC4 | 1.366 | 1.033 | 1.806 | 2.87E-02 |
| GLMN | 1.832 | 1.009 | 3.328 | 4.67E-02 |
| GSDMA | 3.712 | 1.509 | 9.132 | 4.30E-03 |
| GSDMB | 1.609 | 1.216 | 2.129 | 8.78E-04 |
| GSDMD | 1.298 | 1.018 | 1.654 | 3.53E-02 |
| HDAC6 | 4.364 | 2.038 | 9.344 | 1.49E-04 |
| IL13RA2 | 1.359 | 1.033 | 1.786 | 2.81E-02 |
| IRF1 | 1.460 | 1.041 | 2.048 | 2.85E-02 |
| IRF3 | 1.733 | 1.152 | 2.607 | 8.34E-03 |
| JUN | 0.783 | 0.625 | 0.981 | 3.32E-02 |
| MELK | 1.639 | 1.173 | 2.291 | 3.82E-03 |
| MKI67 | 1.754 | 1.299 | 2.368 | 2.48E-04 |
| MST1 | 1.931 | 1.351 | 2.758 | 3.01E-04 |
| NLRP1 | 1.555 | 1.024 | 2.361 | 3.81E-02 |
| NLRP13 | 0.031 | 0.002 | 0.523 | 1.60E-02 |
| PARP1 | 1.769 | 1.058 | 2.960 | 2.97E-02 |
| PTEN | 0.711 | 0.515 | 0.981 | 3.78E-02 |
| PTGS2 | 0.803 | 0.663 | 0.973 | 2.48E-02 |
| RIPK3 | 1.921 | 1.030 | 3.583 | 4.01E-02 |
| TLR2 | 1.537 | 1.019 | 2.320 | 4.04E-02 |
| TP53 | 0.706 | 0.546 | 0.913 | 7.92E-03 |
| TREM2 | 1.879 | 1.433 | 2.464 | 4.98E-06 |
| TRPM2 | 1.310 | 1.020 | 1.683 | 3.45E-02 |
| UBE2D2 | 4.340 | 1.832 | 10.279 | 8.49E-04 |
| VIM | 1.513 | 1.134 | 2.018 | 4.84E-03 |
| ZDHHC1 | 1.987 | 1.021 | 3.869 | 4.33E-02 |
