# Supplementary material for: Identification of ZDHHC1 as a Pyroptosis Inducer and Potential Target in the Establishment of Pyroptosis-Related Signature in Localized Prostate Cancer
Source: Oxid Med Cell Longev. 2022 Dec 22;2022:5925817. doi: 10.1155/2022/5925817 (PMC9800907; doi:10.1155/2022/5925817)
Supplement: Supplementary 5 — Supplementary Table 5: risk grouping in the construction of prognostic signatures. [file 5925817.f5.docx]

|  | | BCR state |  | | | |
| --- | --- | --- | --- | --- | --- | --- |
| ID | bRFS time (y) | (0:no recurrence; | ATG7 | CHMP1A | HDAC6 | IRF1 |
| 1:recerrence) | | | | | | |
| TCGA-KC-A4BR | 2.800 | 1 | 2.462 | 32.926 | 4.693 | 39.316 |
| TCGA-G9-6498 | 3.677 | 1 | 1.875 | 48.084 | 2.257 | 18.605 |
| TCGA-J9-A8CN | 3.389 | 0 | 2.490 | 41.677 | 3.223 | 23.266 |
| TCGA-VP-A87E | 5.581 | 0 | 2.619 | 38.899 | 3.746 | 30.435 |
| TCGA-ZG-A9LB | 1.329 | 0 | 2.574 | 22.612 | 2.125 | 31.908 |
| TCGA-EJ-A46G | 0.255 | 0 | 1.786 | 36.086 | 3.222 | 8.016 |
| TCGA-J4-AATV | 0.981 | 0 | 2.552 | 41.162 | 4.912 | 29.893 |
| TCGA-G9-6377 | 1.419 | 0 | 2.675 | 43.829 | 3.246 | 17.162 |
| TCGA-CH-5753 | 0.085 | 0 | 1.777 | 14.325 | 5.050 | 28.039 |
| TCGA-J4-A83L | 0.866 | 0 | 2.697 | 41.670 | 5.122 | 22.137 |
| TCGA-CH-5789 | 0.833 | 0 | 2.849 | 31.409 | 5.282 | 25.657 |
| TCGA-VP-AA1N | 1.485 | 0 | 3.165 | 41.679 | 5.109 | 27.374 |
| TCGA-XJ-A83H | 3.507 | 0 | 2.419 | 24.628 | 4.176 | 24.049 |
| TCGA-HI-7169 | 5.340 | 0 | 1.109 | 30.431 | 1.938 | 2.294 |
| TCGA-CH-5763 | 1.000 | 0 | 2.525 | 20.915 | 4.477 | 24.915 |
| TCGA-G9-6339 | 4.477 | 1 | 2.482 | 50.472 | 3.948 | 12.013 |
| TCGA-KK-A8IB | 0.227 | 0 | 2.812 | 43.704 | 3.605 | 20.717 |
| TCGA-J4-A67T | 0.501 | 0 | 2.953 | 43.642 | 4.935 | 20.312 |
| TCGA-CH-5743 | 1.164 | 0 | 2.782 | 33.120 | 5.108 | 24.636 |
| TCGA-G9-6333 | 5.274 | 0 | 2.431 | 36.928 | 3.266 | 15.891 |
| TCGA-QU-A6IL | 0.266 | 0 | 2.371 | 33.461 | 2.272 | 22.680 |
| TCGA-G9-7525 | 1.403 | 0 | 1.347 | 44.503 | 4.152 | 2.233 |
| TCGA-ZG-A9LN | 0.315 | 0 | 2.242 | 30.523 | 3.014 | 20.827 |
| TCGA-KK-A8IJ | 4.271 | 0 | 2.635 | 43.473 | 5.106 | 19.238 |
| TCGA-G9-6338 | 4.518 | 0 | 1.782 | 41.245 | 2.408 | 4.857 |
| TCGA-HI-7170 | 4.877 | 0 | 2.825 | 35.854 | 3.404 | 12.455 |
| TCGA-G9-6363 | 2.721 | 0 | 2.111 | 28.522 | 2.288 | 4.794 |
| TCGA-HC-8266 | 0.096 | 0 | 3.277 | 32.271 | 5.897 | 27.300 |
| TCGA-G9-6353 | 2.323 | 0 | 2.376 | 51.989 | 4.570 | 2.307 |
| TCGA-KC-A7F3 | 1.822 | 0 | 2.447 | 37.200 | 4.903 | 20.189 |
| TCGA-V1-A8MF | 3.436 | 0 | 2.311 | 49.820 | 3.600 | 5.737 |
| TCGA-G9-6348 | 3.134 | 0 | 1.828 | 38.814 | 3.691 | 8.784 |
| TCGA-ZG-A9N3 | 0.668 | 0 | 1.842 | 42.844 | 2.805 | 8.777 |
| TCGA-EJ-5521 | 2.307 | 0 | 2.762 | 51.211 | 3.800 | 4.699 |
| TCGA-EJ-7312 | 1.816 | 0 | 2.639 | 57.593 | 4.067 | 2.225 |
| TCGA-KK-A6E8 | 4.849 | 0 | 2.130 | 42.844 | 3.157 | 2.680 |
| TCGA-VN-A88O | 1.318 | 0 | 2.668 | 48.358 | 5.463 | 20.329 |
| TCGA-G9-6347 | 4.707 | 0 | 3.148 | 68.424 | 1.684 | 5.497 |
| TCGA-G9-6378 | 1.819 | 0 | 1.870 | 41.892 | 4.008 | 3.094 |
| TCGA-2A-AAYO | 2.526 | 0 | 2.425 | 50.053 | 4.535 | 6.184 |
| TCGA-X4-A8KQ | 3.099 | 1 | 1.907 | 19.557 | 3.416 | 21.206 |
| TCGA-V1-A8WN | 1.304 | 0 | 2.475 | 35.254 | 5.499 | 15.105 |
| TCGA-VN-A88P | 2.512 | 0 | 2.212 | 48.135 | 4.289 | 5.296 |
| TCGA-G9-6496 | 2.778 | 0 | 2.085 | 42.540 | 2.652 | 3.319 |
| TCGA-KK-A8ID | 5.405 | 0 | 1.493 | 35.192 | 2.427 | 7.431 |
| TCGA-G9-6499 | 3.038 | 0 | 2.836 | 53.382 | 3.314 | 6.214 |
| TCGA-KC-A4BN | 4.973 | 0 | 2.619 | 43.349 | 6.012 | 14.772 |
| TCGA-KK-A8IG | 6.058 | 0 | 2.391 | 35.593 | 3.166 | 8.337 |

| TCGA-G9-7523 | 0.888 | 0 | 1.904 | 32.326 | 2.569 | 4.359 |
| --- | --- | --- | --- | --- | --- | --- |
| TCGA-G9-7510 | 1.847 | 0 | 3.233 | 46.291 | 3.948 | 8.329 |
| TCGA-G9-6366 | 0.277 | 0 | 2.957 | 54.895 | 3.937 | 3.655 |
| TCGA-G9-7519 | 1.252 | 0 | 2.340 | 45.232 | 4.015 | 4.184 |
| TCGA-KK-A59X | 4.690 | 0 | 2.820 | 52.919 | 4.270 | 4.001 |
| TCGA-EJ-AB20 | 0.359 | 0 | 2.046 | 34.996 | 3.558 | 5.566 |
| TCGA-KK-A8IL | 1.721 | 0 | 2.983 | 44.820 | 5.530 | 22.473 |
| TCGA-M7-A723 | 0.170 | 0 | 2.553 | 39.811 | 2.787 | 6.792 |
| TCGA-ZG-A9NI | 0.362 | 0 | 2.201 | 35.405 | 3.314 | 3.534 |
| TCGA-KK-A6E3 | 5.633 | 0 | 2.509 | 56.539 | 3.555 | 5.260 |
| TCGA-G9-6367 | 2.397 | 0 | 2.870 | 42.001 | 3.968 | 3.199 |
| TCGA-CH-5772 | 1.332 | 0 | 2.127 | 41.498 | 4.504 | 5.939 |
| TCGA-KK-A59Z | 5.148 | 0 | 2.842 | 35.113 | 2.972 | 12.862 |
| TCGA-VN-A88I | 0.737 | 0 | 2.358 | 40.768 | 4.323 | 7.564 |
| TCGA-EJ-7218 | 6.964 | 0 | 2.675 | 50.079 | 4.536 | 4.081 |
| TCGA-CH-5767 | 1.255 | 0 | 2.666 | 32.338 | 3.443 | 8.784 |
| TCGA-CH-5751 | 2.918 | 0 | 2.755 | 26.744 | 3.552 | 15.459 |
| TCGA-V1-A9OQ | 1.016 | 0 | 2.229 | 40.102 | 2.746 | 2.783 |
| TCGA-KK-A6E6 | 9.444 | 0 | 1.620 | 26.013 | 4.677 | 4.940 |
| TCGA-G9-6364 | 2.304 | 0 | 2.801 | 33.282 | 3.634 | 2.831 |
| TCGA-J4-A67Q | 1.710 | 0 | 2.735 | 61.179 | 2.717 | 5.315 |
| TCGA-HC-7210 | 0.408 | 0 | 2.809 | 30.406 | 4.917 | 14.501 |
| TCGA-KK-A8I9 | 1.975 | 1 | 2.562 | 53.027 | 4.801 | 12.884 |
| TCGA-KK-A8I4 | 3.164 | 0 | 2.355 | 34.033 | 4.772 | 11.498 |
| TCGA-G9-6343 | 1.518 | 0 | 2.113 | 33.224 | 3.081 | 1.981 |
| TCGA-XA-A8JR | 0.326 | 0 | 2.330 | 40.407 | 4.310 | 8.164 |
| TCGA-2A-A8VX | 2.830 | 0 | 1.611 | 38.151 | 4.101 | 1.080 |
| TCGA-G9-6336 | 4.573 | 0 | 2.443 | 40.334 | 4.317 | 4.397 |
| TCGA-VP-A87C | 4.630 | 0 | 3.054 | 38.965 | 4.994 | 20.769 |
| TCGA-V1-A8WL | 3.767 | 0 | 1.882 | 44.426 | 5.167 | 6.710 |
| TCGA-HC-7750 | 0.093 | 0 | 2.636 | 40.934 | 3.853 | 3.044 |
| TCGA-ZG-A9LM | 0.332 | 0 | 2.448 | 39.403 | 3.640 | 6.004 |
| TCGA-VN-A88L | 1.079 | 0 | 2.126 | 37.530 | 4.055 | 2.713 |
| TCGA-HC-7747 | 0.156 | 0 | 2.997 | 39.367 | 4.423 | 7.545 |
| TCGA-G9-6369 | 2.332 | 0 | 2.313 | 49.804 | 2.245 | 1.609 |
| TCGA-G9-6362 | 2.252 | 1 | 0.899 | 15.017 | 2.869 | 3.080 |
| TCGA-EJ-7125 | 5.096 | 0 | 2.495 | 42.416 | 4.072 | 5.618 |
| TCGA-ZG-A9L2 | 0.493 | 1 | 1.869 | 32.671 | 4.145 | 4.673 |
| TCGA-G9-6329 | 2.529 | 0 | 2.007 | 22.433 | 3.279 | 6.376 |
| TCGA-G9-6354 | 4.112 | 0 | 2.176 | 25.377 | 3.129 | 3.680 |
| TCGA-HC-7079 | 0.345 | 1 | 4.528 | 30.324 | 5.785 | 37.638 |
| TCGA-G9-7522 | 1.238 | 0 | 2.571 | 37.451 | 4.277 | 6.028 |
| TCGA-KK-A6E4 | 7.542 | 0 | 3.397 | 52.581 | 4.329 | 4.403 |
| TCGA-J4-A83M | 1.197 | 1 | 1.765 | 37.893 | 5.166 | 9.555 |
| TCGA-G9-6356 | 2.740 | 0 | 2.814 | 38.855 | 4.284 | 7.881 |
| TCGA-J9-A8CL | 0.362 | 1 | 2.271 | 42.358 | 3.650 | 5.255 |
| TCGA-XK-AAJT | 3.085 | 0 | 2.414 | 46.163 | 4.226 | 6.255 |
| TCGA-J4-AAU2 | 0.833 | 0 | 2.602 | 51.869 | 4.939 | 5.633 |
| TCGA-G9-6361 | 2.762 | 0 | 2.435 | 32.594 | 3.437 | 2.199 |
| TCGA-ZG-A9L6 | 1.819 | 1 | 2.862 | 45.801 | 4.385 | 5.010 |
| TCGA-G9-6385 | 0.975 | 0 | 2.758 | 47.860 | 5.146 | 3.097 |

| TCGA-ZG-A8QW | 0.137 | 0 | 3.296 | 50.577 | 4.763 | 10.739 |
| --- | --- | --- | --- | --- | --- | --- |
| TCGA-G9-7509 | 3.189 | 0 | 2.344 | 39.560 | 5.036 | 1.772 |
| TCGA-CH-5744 | 0.164 | 0 | 2.678 | 34.844 | 6.122 | 16.857 |
| TCGA-H9-A6BX | 1.562 | 0 | 2.879 | 39.880 | 4.375 | 7.974 |
| TCGA-KK-A7AZ | 2.301 | 0 | 1.856 | 42.185 | 3.309 | 2.546 |
| TCGA-H9-7775 | 0.121 | 0 | 1.527 | 34.641 | 5.109 | 1.763 |
| TCGA-G9-6371 | 2.318 | 0 | 2.346 | 36.876 | 4.138 | 3.591 |
| TCGA-CH-5766 | 0.085 | 0 | 2.769 | 37.621 | 5.325 | 7.881 |
| TCGA-EJ-A65J | 0.249 | 0 | 3.069 | 42.990 | 4.000 | 2.909 |
| TCGA-XJ-A83F | 2.088 | 0 | 2.080 | 41.751 | 4.158 | 2.382 |
| TCGA-KK-A59V | 7.833 | 0 | 2.362 | 42.537 | 4.913 | 20.915 |
| TCGA-FC-A4JI | 0.079 | 0 | 2.258 | 31.151 | 2.608 | 2.972 |
| TCGA-2A-AAYF | 2.663 | 0 | 2.684 | 48.359 | 5.564 | 2.468 |
| TCGA-KC-A7FD | 0.740 | 0 | 2.370 | 40.130 | 4.979 | 7.917 |
| TCGA-KK-A7B2 | 1.896 | 1 | 2.892 | 44.852 | 5.019 | 15.380 |
| TCGA-J4-A6G3 | 1.447 | 1 | 2.931 | 42.136 | 5.701 | 14.981 |
| TCGA-2A-A8VO | 3.649 | 0 | 1.898 | 44.941 | 4.997 | 6.265 |
| TCGA-M7-A725 | 0.263 | 0 | 2.566 | 49.859 | 5.999 | 4.694 |
| TCGA-G9-6365 | 2.203 | 0 | 2.584 | 31.700 | 3.436 | 3.970 |
| TCGA-HC-8264 | 0.132 | 0 | 2.729 | 36.343 | 4.141 | 2.957 |
| TCGA-VP-A878 | 0.268 | 1 | 2.776 | 44.919 | 4.921 | 10.512 |
| TCGA-KC-A7FA | 1.638 | 0 | 2.303 | 35.962 | 5.920 | 11.020 |
| TCGA-ZG-A8QX | 0.411 | 0 | 2.767 | 35.985 | 3.719 | 5.290 |
| TCGA-VP-A872 | 8.912 | 0 | 2.102 | 52.191 | 5.906 | 7.634 |
| TCGA-V1-A8MK | 1.392 | 0 | 2.414 | 42.394 | 5.033 | 4.944 |
| TCGA-KK-A5A1 | 6.477 | 0 | 1.857 | 24.822 | 1.787 | 3.289 |
| TCGA-EJ-A8FP | 0.321 | 1 | 2.495 | 41.073 | 4.781 | 3.890 |
| TCGA-EJ-7792 | 1.441 | 0 | 2.269 | 34.225 | 4.402 | 4.039 |
| TCGA-V1-A8ML | 1.227 | 0 | 2.177 | 45.272 | 4.909 | 3.601 |
| TCGA-4L-AA1F | 0.959 | 0 | 2.729 | 40.236 | 4.296 | 5.411 |
| TCGA-KK-A6E5 | 4.104 | 0 | 2.704 | 53.635 | 4.738 | 4.195 |
| TCGA-EJ-A6RC | 1.899 | 0 | 2.850 | 47.114 | 4.743 | 3.795 |
| TCGA-V1-A8MM | 2.712 | 1 | 2.041 | 51.301 | 4.717 | 6.054 |
| TCGA-HC-8265 | 0.192 | 0 | 2.811 | 3.659 | 1.953 | 13.456 |
| TCGA-HC-7081 | 0.197 | 0 | 2.450 | 30.609 | 4.971 | 4.143 |
| TCGA-V1-A9Z8 | 0.512 | 0 | 2.785 | 46.705 | 3.707 | 4.732 |
| TCGA-EJ-7315 | 0.792 | 0 | 3.203 | 44.626 | 4.162 | 5.147 |
| TCGA-CH-5737 | 0.249 | 0 | 2.987 | 39.928 | 4.771 | 4.434 |
| TCGA-ZG-A8QZ | 0.844 | 0 | 2.184 | 33.841 | 4.778 | 8.990 |
| TCGA-CH-5750 | 1.085 | 0 | 3.004 | 28.988 | 4.937 | 12.701 |
| TCGA-G9-6370 | 2.208 | 0 | 2.962 | 42.736 | 4.944 | 4.610 |
| TCGA-VN-A88R | 1.258 | 1 | 2.948 | 51.328 | 4.844 | 2.289 |
| TCGA-HC-A8CY | 0.795 | 0 | 2.241 | 45.140 | 4.555 | 6.861 |
| TCGA-EJ-A65B | 1.468 | 0 | 3.372 | 62.680 | 3.445 | 3.288 |
| TCGA-VN-A88Q | 2.742 | 0 | 1.414 | 18.931 | 4.992 | 7.132 |
| TCGA-KK-A7AW | 0.559 | 0 | 3.638 | 52.719 | 2.543 | 7.296 |
| TCGA-QU-A6IM | 3.416 | 0 | 3.716 | 77.376 | 3.063 | 13.244 |
| TCGA-EJ-5510 | 2.022 | 0 | 3.022 | 32.168 | 4.245 | 6.096 |
| TCGA-2A-AAYU | 1.685 | 0 | 2.662 | 46.509 | 5.758 | 2.457 |
| TCGA-V1-A9OX | 0.701 | 0 | 2.388 | 50.643 | 4.919 | 4.813 |
| TCGA-EJ-5512 | 2.142 | 0 | 2.758 | 35.587 | 4.422 | 4.302 |

| TCGA-HC-7748 | 0.170 | 0 | 1.960 | 25.220 | 5.677 | 8.476 |
| --- | --- | --- | --- | --- | --- | --- |
| TCGA-EJ-5497 | 1.110 | 0 | 2.053 | 26.773 | 4.309 | 3.078 |
| TCGA-EJ-AB27 | 0.397 | 0 | 2.629 | 47.850 | 5.299 | 5.546 |
| TCGA-TK-A8OK | 0.074 | 0 | 2.061 | 23.052 | 3.602 | 5.308 |
| TCGA-EJ-5515 | 2.907 | 0 | 2.680 | 32.715 | 3.987 | 3.120 |
| TCGA-J9-A8CP | 1.058 | 0 | 2.516 | 25.508 | 3.571 | 8.098 |
| TCGA-J9-A52C | 0.488 | 0 | 2.951 | 39.077 | 4.071 | 15.707 |
| TCGA-EJ-5498 | 1.471 | 0 | 2.429 | 21.938 | 3.990 | 6.045 |
| TCGA-TP-A8TT | 0.992 | 0 | 2.581 | 36.085 | 6.055 | 12.450 |
| TCGA-VN-A88M | 0.501 | 0 | 1.794 | 34.354 | 4.388 | 3.391 |
| TCGA-ZG-A9ND | 0.715 | 0 | 3.723 | 53.971 | 4.727 | 11.318 |
| TCGA-XK-AAK1 | 1.682 | 0 | 3.310 | 51.925 | 4.100 | 3.558 |
| TCGA-G9-6351 | 3.329 | 0 | 2.130 | 35.694 | 4.741 | 2.546 |
| TCGA-YL-A8SK | 3.164 | 0 | 2.503 | 42.435 | 6.052 | 14.227 |
| TCGA-HC-7231 | 0.197 | 0 | 2.768 | 37.484 | 5.409 | 4.908 |
| TCGA-EJ-A65G | 0.762 | 0 | 2.713 | 41.528 | 3.990 | 1.552 |
| TCGA-HC-7078 | 0.444 | 0 | 2.565 | 45.186 | 4.890 | 3.776 |
| TCGA-J4-A83J | 0.849 | 0 | 3.161 | 24.807 | 5.418 | 21.254 |
| TCGA-FC-7708 | 0.088 | 0 | 2.727 | 33.954 | 5.486 | 9.720 |
| TCGA-KK-A59Y | 3.723 | 0 | 2.335 | 29.142 | 5.273 | 3.556 |
| TCGA-KK-A7AQ | 3.334 | 1 | 2.562 | 69.677 | 3.906 | 3.286 |
| TCGA-J4-A67M | 1.337 | 0 | 3.189 | 56.789 | 3.435 | 5.474 |
| TCGA-EJ-7786 | 0.216 | 0 | 2.595 | 35.764 | 5.107 | 2.367 |
| TCGA-EJ-5499 | 1.460 | 0 | 3.045 | 26.012 | 3.583 | 7.693 |
| TCGA-KC-A7FE | 0.964 | 0 | 2.902 | 47.244 | 5.196 | 4.160 |
| TCGA-EJ-7791 | 0.595 | 0 | 2.923 | 37.070 | 4.937 | 5.041 |
| TCGA-CH-5738 | 0.581 | 0 | 3.059 | 24.651 | 4.786 | 11.993 |
| TCGA-HC-A6HY | 0.367 | 0 | 2.571 | 57.045 | 5.542 | 3.823 |
| TCGA-EJ-5503 | 1.452 | 0 | 2.853 | 48.364 | 6.642 | 5.060 |
| TCGA-KK-A7B3 | 1.745 | 1 | 2.230 | 55.086 | 5.885 | 5.116 |
| TCGA-WW-A8ZI | 0.482 | 0 | 2.446 | 47.583 | 5.183 | 4.984 |
| TCGA-J4-8200 | 0.537 | 0 | 2.696 | 28.283 | 3.267 | 5.237 |
| TCGA-HC-A6AS | 0.121 | 0 | 3.652 | 58.640 | 3.721 | 8.666 |
| TCGA-G9-6373 | 1.384 | 0 | 1.928 | 20.299 | 3.606 | 4.417 |
| TCGA-G9-6494 | 3.951 | 0 | 3.003 | 41.654 | 4.594 | 1.700 |
| TCGA-CH-5792 | 0.249 | 0 | 3.746 | 43.731 | 5.190 | 9.501 |
| TCGA-EJ-5494 | 1.036 | 0 | 3.091 | 40.898 | 5.879 | 7.266 |
| TCGA-EJ-7784 | 0.458 | 0 | 2.764 | 33.442 | 4.842 | 4.480 |
| TCGA-XJ-A83G | 3.170 | 0 | 1.985 | 38.486 | 4.447 | 2.727 |
| TCGA-G9-6332 | 6.378 | 1 | 2.372 | 26.462 | 3.852 | 1.688 |
| TCGA-ZG-A9LU | 1.504 | 0 | 2.837 | 41.931 | 4.632 | 12.425 |
| TCGA-J4-A67O | 1.345 | 0 | 2.448 | 54.267 | 2.829 | 4.540 |
| TCGA-2A-A8VV | 1.838 | 0 | 2.293 | 33.913 | 4.540 | 1.871 |
| TCGA-J4-AATZ | 0.216 | 1 | 2.684 | 47.323 | 6.134 | 3.933 |
| TCGA-HC-7818 | 0.123 | 0 | 2.429 | 29.681 | 4.800 | 7.481 |
| TCGA-HC-A6AQ | 0.290 | 0 | 2.839 | 56.367 | 4.140 | 2.794 |
| TCGA-EJ-5517 | 2.329 | 0 | 2.833 | 31.579 | 3.450 | 2.670 |
| TCGA-XJ-A9DQ | 0.249 | 0 | 1.704 | 42.751 | 5.409 | 2.142 |
| TCGA-EJ-7328 | 0.466 | 0 | 2.582 | 28.500 | 4.944 | 8.369 |
| TCGA-J4-A67K | 1.814 | 0 | 2.765 | 43.513 | 3.156 | 6.907 |
| TCGA-J4-A83N | 1.690 | 0 | 2.281 | 36.985 | 4.849 | 5.340 |

| TCGA-ZG-A9L9 | 0.140 | 1 | 2.417 | 30.179 | 3.817 | 8.769 |
| --- | --- | --- | --- | --- | --- | --- |
| TCGA-ZG-A9L1 | 3.142 | 0 | 1.978 | 19.969 | 4.810 | 4.689 |
| TCGA-EJ-7330 | 0.523 | 0 | 3.367 | 31.282 | 4.194 | 4.599 |
| TCGA-V1-A8MU | 3.833 | 1 | 2.485 | 39.294 | 5.326 | 7.596 |
| TCGA-KC-A7F5 | 0.249 | 0 | 2.211 | 58.464 | 4.939 | 2.779 |
| TCGA-KK-A7AV | 2.252 | 0 | 2.703 | 41.478 | 5.926 | 11.774 |
| TCGA-2A-A8VL | 1.701 | 0 | 2.388 | 40.632 | 5.632 | 3.705 |
| TCGA-EJ-A8FN | 0.556 | 0 | 2.536 | 39.981 | 4.915 | 3.652 |
| TCGA-VN-A943 | 0.362 | 0 | 3.253 | 56.935 | 5.887 | 4.037 |
| TCGA-2A-A8W1 | 0.307 | 0 | 1.976 | 32.808 | 4.874 | 2.682 |
| TCGA-HC-7817 | 0.088 | 0 | 2.954 | 32.575 | 4.197 | 6.732 |
| TCGA-G9-6384 | 1.068 | 0 | 2.804 | 21.473 | 5.086 | 11.396 |
| TCGA-YL-A9WJ | 0.208 | 0 | 3.089 | 39.868 | 5.080 | 10.194 |
| TCGA-J9-A52B | 0.233 | 1 | 1.767 | 32.446 | 7.367 | 6.385 |
| TCGA-QU-A6IP | 7.178 | 0 | 2.656 | 44.211 | 4.688 | 3.665 |
| TCGA-KK-A8IF | 1.775 | 1 | 2.322 | 23.701 | 3.489 | 2.198 |
| TCGA-EJ-7793 | 0.312 | 0 | 2.688 | 35.960 | 5.157 | 1.559 |
| TCGA-EJ-7782 | 0.959 | 0 | 2.548 | 38.972 | 6.098 | 3.776 |
| TCGA-YL-A8SI | 3.899 | 1 | 2.855 | 42.640 | 4.420 | 4.502 |
| TCGA-HC-A8D1 | 0.460 | 0 | 2.581 | 28.039 | 4.977 | 7.748 |
| TCGA-FC-A66V | 0.334 | 0 | 3.156 | 66.424 | 4.968 | 4.660 |
| TCGA-HC-7749 | 0.164 | 0 | 3.120 | 27.907 | 4.565 | 4.511 |
| TCGA-EJ-7314 | 0.808 | 0 | 2.976 | 38.153 | 5.186 | 7.794 |
| TCGA-HC-7232 | 0.244 | 1 | 2.528 | 24.961 | 4.497 | 2.492 |
| TCGA-HC-7820 | 0.279 | 0 | 2.906 | 42.240 | 5.597 | 3.700 |
| TCGA-J4-A67L | 1.419 | 0 | 3.185 | 65.208 | 5.329 | 8.915 |
| TCGA-YL-A8HO | 2.926 | 1 | 3.244 | 46.640 | 3.953 | 4.597 |
| TCGA-CH-5745 | 0.249 | 0 | 2.890 | 36.481 | 4.360 | 8.008 |
| TCGA-KK-A8I7 | 2.981 | 1 | 2.613 | 31.033 | 4.595 | 5.567 |
| TCGA-H9-A6BY | 0.307 | 0 | 2.337 | 42.849 | 3.491 | 1.556 |
| TCGA-EJ-5502 | 0.671 | 0 | 2.917 | 35.652 | 5.358 | 7.646 |
| TCGA-EJ-7317 | 0.781 | 0 | 3.301 | 46.625 | 5.073 | 1.785 |
| TCGA-ZG-A9LS | 0.578 | 0 | 3.344 | 34.202 | 3.820 | 9.910 |
| TCGA-HC-8213 | 0.003 | 0 | 2.626 | 26.070 | 3.410 | 2.039 |
| TCGA-KK-A7AY | 3.079 | 1 | 2.323 | 22.575 | 3.475 | 4.369 |
| TCGA-KK-A7B0 | 1.660 | 1 | 4.369 | 64.780 | 3.217 | 7.777 |
| TCGA-CH-5771 | 1.085 | 0 | 3.446 | 29.431 | 4.526 | 7.139 |
| TCGA-EJ-A7NG | 0.723 | 0 | 2.655 | 43.045 | 5.454 | 4.470 |
| TCGA-EJ-A6RA | 0.967 | 0 | 2.972 | 46.073 | 5.060 | 3.568 |
| TCGA-HC-8256 | 0.260 | 0 | 2.749 | 41.671 | 5.248 | 2.625 |
| TCGA-EJ-5516 | 2.145 | 0 | 2.620 | 14.285 | 3.695 | 6.086 |
| TCGA-ZG-A9LY | 0.786 | 0 | 2.732 | 26.609 | 3.793 | 5.331 |
| TCGA-HC-7752 | 0.268 | 0 | 1.435 | 34.481 | 4.605 | 1.165 |
| TCGA-CH-5762 | 3.668 | 0 | 2.862 | 20.963 | 3.759 | 8.667 |
| TCGA-HC-A6AL | 0.184 | 0 | 2.836 | 55.285 | 3.926 | 3.299 |
| TCGA-V1-A9O7 | 2.523 | 1 | 2.745 | 23.448 | 4.569 | 11.682 |
| TCGA-YL-A8SB | 3.792 | 1 | 2.449 | 39.960 | 5.725 | 2.832 |
| TCGA-FC-A5OB | 0.748 | 0 | 2.972 | 43.047 | 3.853 | 0.836 |
| TCGA-EJ-5504 | 0.211 | 1 | 3.018 | 31.764 | 3.812 | 3.816 |
| TCGA-ZG-A9MC | 1.173 | 0 | 3.522 | 29.999 | 4.477 | 19.533 |
| TCGA-EJ-7321 | 0.638 | 0 | 2.476 | 24.588 | 4.677 | 6.506 |

| TCGA-EJ-A8FU | 0.225 | 0 | 2.576 | 36.005 | 5.544 | 5.978 |
| --- | --- | --- | --- | --- | --- | --- |
| TCGA-CH-5769 | 0.170 | 0 | 2.738 | 35.050 | 5.015 | 6.277 |
| TCGA-ZG-A9L4 | 3.036 | 0 | 2.749 | 26.759 | 4.192 | 5.640 |
| TCGA-EJ-A8FS | 0.592 | 1 | 2.691 | 41.083 | 5.324 | 2.095 |
| TCGA-YL-A9WK | 2.764 | 1 | 2.202 | 23.918 | 3.812 | 2.220 |
| TCGA-HC-8257 | 0.079 | 0 | 2.199 | 36.088 | 6.509 | 4.992 |
| TCGA-VP-A875 | 2.205 | 0 | 2.150 | 35.523 | 5.072 | 3.943 |
| TCGA-EJ-7331 | 0.518 | 0 | 2.876 | 28.684 | 4.662 | 4.879 |
| TCGA-QU-A6IN | 9.553 | 0 | 2.782 | 51.621 | 3.720 | 3.061 |
| TCGA-CH-5764 | 0.085 | 0 | 2.552 | 17.591 | 4.611 | 8.112 |
| TCGA-J9-A52E | 0.244 | 1 | 3.369 | 37.551 | 3.588 | 4.487 |
| TCGA-Y6-A8TL | 2.123 | 0 | 2.849 | 45.171 | 4.802 | 4.910 |
| TCGA-HC-7745 | 0.173 | 0 | 2.905 | 32.178 | 4.772 | 7.066 |
| TCGA-EJ-5509 | 3.019 | 0 | 3.821 | 41.753 | 4.487 | 4.213 |
| TCGA-YL-A9WH | 1.170 | 0 | 3.467 | 52.722 | 5.423 | 10.559 |
| TCGA-X4-A8KS | 2.318 | 0 | 1.663 | 34.399 | 3.942 | 3.747 |
| TCGA-G9-6379 | 3.816 | 0 | 2.773 | 61.084 | 3.437 | 10.108 |
| TCGA-EJ-A7NK | 0.808 | 0 | 2.379 | 44.490 | 6.119 | 7.213 |
| TCGA-CH-5740 | 0.085 | 0 | 1.738 | 21.136 | 6.186 | 5.338 |
| TCGA-EJ-A46D | 0.353 | 0 | 2.533 | 37.476 | 5.989 | 2.711 |
| TCGA-ZG-A9M4 | 0.611 | 0 | 1.871 | 25.953 | 7.824 | 10.691 |
| TCGA-EJ-8474 | 0.860 | 0 | 2.441 | 27.771 | 3.960 | 3.132 |
| TCGA-EJ-5527 | 1.088 | 0 | 2.662 | 21.978 | 4.034 | 4.317 |
| TCGA-KK-A8IC | 2.904 | 1 | 3.145 | 34.837 | 4.869 | 6.414 |
| TCGA-EJ-7788 | 0.142 | 0 | 2.630 | 30.477 | 4.970 | 2.905 |
| TCGA-KK-A8I5 | 6.995 | 0 | 2.583 | 34.236 | 4.541 | 6.097 |
| TCGA-V1-A9OH | 3.433 | 0 | 2.141 | 24.337 | 3.943 | 3.857 |
| TCGA-V1-A9O9 | 0.559 | 0 | 2.609 | 38.767 | 6.252 | 9.277 |
| TCGA-V1-A9ZG | 3.060 | 0 | 2.474 | 37.920 | 4.807 | 4.878 |
| TCGA-V1-A9OF | 3.244 | 0 | 2.972 | 51.491 | 6.006 | 1.595 |
| TCGA-M7-A721 | 0.559 | 0 | 2.935 | 40.304 | 4.973 | 3.307 |
| TCGA-EJ-5505 | 1.227 | 0 | 3.128 | 39.317 | 5.292 | 1.861 |
| TCGA-HC-7233 | 0.142 | 0 | 3.304 | 24.805 | 4.383 | 10.664 |
| TCGA-HC-7737 | 0.175 | 0 | 3.314 | 35.680 | 4.529 | 7.790 |
| TCGA-EJ-A46I | 1.830 | 0 | 2.521 | 39.249 | 5.903 | 5.075 |
| TCGA-G9-6342 | 3.666 | 0 | 2.704 | 28.367 | 4.370 | 4.104 |
| TCGA-HC-8261 | 0.170 | 0 | 3.287 | 46.623 | 5.204 | 4.449 |
| TCGA-YL-A8HJ | 4.414 | 0 | 2.487 | 28.572 | 4.063 | 3.974 |
| TCGA-EJ-A7NJ | 0.540 | 0 | 2.569 | 36.453 | 6.787 | 7.187 |
| TCGA-EJ-5532 | 2.537 | 0 | 3.259 | 41.537 | 5.081 | 3.138 |
| TCGA-CH-5748 | 0.085 | 0 | 2.767 | 35.014 | 5.443 | 3.923 |
| TCGA-HC-A76X | 0.411 | 0 | 1.471 | 32.634 | 5.694 | 1.065 |
| TCGA-J4-A67R | 1.244 | 0 | 2.763 | 43.712 | 3.869 | 7.072 |
| TCGA-EJ-5495 | 1.660 | 0 | 2.771 | 15.731 | 4.071 | 7.061 |
| TCGA-KK-A6DY | 9.655 | 0 | 2.867 | 28.476 | 6.640 | 17.639 |
| TCGA-CH-5794 | 2.416 | 0 | 2.899 | 38.831 | 6.518 | 4.008 |
| TCGA-XJ-A9DX | 2.164 | 1 | 0.918 | 21.951 | 3.108 | 2.331 |
| TCGA-CH-5741 | 1.082 | 0 | 2.728 | 31.360 | 5.453 | 1.927 |
| TCGA-EJ-5524 | 2.285 | 0 | 2.615 | 17.838 | 3.882 | 4.598 |
| TCGA-KK-A8IH | 4.479 | 0 | 2.797 | 44.335 | 7.513 | 7.069 |
| TCGA-V1-A8WW | 0.288 | 1 | 3.164 | 50.183 | 7.438 | 8.485 |

| TCGA-XK-AAJ3 | 2.378 | 0 | 3.190 | 50.298 | 6.147 | 2.837 |
| --- | --- | --- | --- | --- | --- | --- |
| TCGA-XQ-A8TB | 2.110 | 0 | 2.494 | 27.082 | 4.907 | 8.841 |
| TCGA-EJ-A65E | 1.142 | 0 | 3.249 | 39.738 | 5.244 | 4.026 |
| TCGA-VP-A87J | 3.551 | 0 | 2.639 | 15.438 | 3.875 | 9.529 |
| TCGA-KK-A7B1 | 2.912 | 0 | 2.931 | 37.777 | 5.818 | 8.137 |
| TCGA-EJ-5531 | 1.984 | 0 | 3.766 | 42.512 | 5.374 | 5.554 |
| TCGA-HC-8259 | 0.337 | 0 | 2.836 | 41.478 | 5.975 | 2.585 |
| TCGA-EJ-A65M | 0.630 | 0 | 3.381 | 45.096 | 3.386 | 4.503 |
| TCGA-EJ-7115 | 2.252 | 0 | 3.194 | 45.401 | 5.098 | 2.619 |
| TCGA-CH-5754 | 0.170 | 0 | 3.210 | 24.261 | 5.326 | 14.370 |
| TCGA-VP-A876 | 7.058 | 0 | 1.544 | 20.069 | 6.083 | 2.599 |
| TCGA-VP-A879 | 1.995 | 0 | 2.083 | 29.871 | 5.883 | 3.451 |
| TCGA-G9-7521 | 1.334 | 0 | 3.269 | 22.142 | 4.604 | 13.417 |
| TCGA-HC-7738 | 0.222 | 1 | 2.473 | 36.428 | 4.955 | 2.149 |
| TCGA-YL-A8S9 | 5.047 | 0 | 1.912 | 19.461 | 4.549 | 1.096 |
| TCGA-KK-A6E1 | 4.545 | 0 | 3.464 | 38.892 | 5.198 | 4.198 |
| TCGA-VP-A87B | 6.326 | 0 | 2.721 | 51.810 | 4.268 | 2.265 |
| TCGA-VP-A87H | 1.816 | 0 | 4.215 | 41.790 | 3.661 | 8.151 |
| TCGA-EJ-7797 | 0.512 | 0 | 2.895 | 35.235 | 5.470 | 4.384 |
| TCGA-KK-A6E7 | 5.589 | 1 | 2.695 | 55.276 | 4.439 | 2.473 |
| TCGA-EJ-5496 | 1.630 | 0 | 2.694 | 17.977 | 4.306 | 2.067 |
| TCGA-HC-7819 | 0.003 | 0 | 2.755 | 28.909 | 5.933 | 4.875 |
| TCGA-HC-8262 | 0.208 | 0 | 2.433 | 32.845 | 5.306 | 3.741 |
| TCGA-EJ-A7NF | 0.592 | 0 | 1.880 | 21.374 | 5.293 | 2.813 |
| TCGA-J4-A67S | 1.433 | 1 | 3.287 | 55.856 | 5.098 | 6.794 |
| TCGA-FC-A8O0 | 0.148 | 0 | 2.482 | 47.663 | 7.061 | 3.978 |
| TCGA-J4-A83K | 0.564 | 0 | 2.214 | 38.103 | 6.228 | 4.125 |
| TCGA-EJ-A46B | 0.444 | 0 | 2.751 | 43.630 | 6.334 | 1.586 |
| TCGA-HC-7075 | 0.173 | 0 | 2.964 | 45.860 | 6.131 | 2.471 |
| TCGA-KK-A8IK | 4.282 | 0 | 1.905 | 22.078 | 3.446 | 1.076 |
| TCGA-CH-5746 | 2.003 | 0 | 2.552 | 34.042 | 6.079 | 3.339 |
| TCGA-YJ-A8SW | 0.405 | 0 | 3.099 | 33.072 | 2.417 | 3.147 |
| TCGA-KC-A7F6 | 0.721 | 0 | 1.827 | 33.996 | 5.549 | 6.621 |
| TCGA-EJ-7781 | 0.463 | 0 | 2.986 | 32.473 | 6.085 | 4.341 |
| TCGA-HC-A6AO | 0.458 | 0 | 3.534 | 30.037 | 3.847 | 10.315 |
| TCGA-J9-A8CK | 0.879 | 0 | 2.269 | 31.143 | 4.184 | 3.678 |
| TCGA-HC-A9TH | 0.808 | 1 | 2.577 | 41.422 | 7.650 | 7.597 |
| TCGA-V1-A9OY | 0.466 | 0 | 2.747 | 42.493 | 6.249 | 3.663 |
| TCGA-EJ-7785 | 0.710 | 0 | 2.775 | 15.827 | 5.047 | 5.509 |
| TCGA-HC-A631 | 0.148 | 0 | 3.490 | 42.617 | 5.048 | 4.739 |
| TCGA-HC-7209 | 0.112 | 0 | 2.590 | 21.049 | 5.574 | 7.078 |
| TCGA-V1-A9OA | 1.742 | 0 | 2.475 | 34.191 | 4.242 | 3.598 |
| TCGA-G9-A9S4 | 2.074 | 0 | 1.557 | 19.795 | 4.858 | 3.311 |
| TCGA-HC-7742 | 0.310 | 1 | 2.798 | 30.513 | 5.194 | 3.423 |
| TCGA-YL-A8HM | 3.079 | 1 | 3.341 | 38.304 | 4.882 | 8.059 |
| TCGA-J4-8198 | 0.268 | 0 | 2.560 | 33.011 | 5.781 | 2.969 |
| TCGA-EJ-5506 | 0.975 | 0 | 2.848 | 30.027 | 5.539 | 5.063 |
| TCGA-YL-A9WI | 3.449 | 0 | 3.490 | 35.012 | 3.553 | 2.678 |
| TCGA-EJ-5526 | 1.553 | 0 | 3.128 | 23.971 | 4.151 | 3.469 |
| TCGA-V1-A8MG | 2.348 | 0 | 2.247 | 34.449 | 5.441 | 8.251 |
| TCGA-CH-5768 | 2.003 | 0 | 3.035 | 31.409 | 4.482 | 3.518 |

| TCGA-J9-A52D | 0.581 | 0 | 3.708 | 49.612 | 4.840 | 1.458 |
| --- | --- | --- | --- | --- | --- | --- |
| TCGA-VN-A88K | 2.126 | 0 | 2.798 | 15.052 | 3.851 | 5.285 |
| TCGA-J4-A6G1 | 1.110 | 0 | 3.036 | 31.658 | 5.121 | 10.854 |
| TCGA-EJ-A8FO | 0.427 | 0 | 3.255 | 40.888 | 6.161 | 2.131 |
| TCGA-M7-A722 | 1.532 | 1 | 2.957 | 51.265 | 2.546 | 1.747 |
| TCGA-HC-7821 | 0.153 | 1 | 2.501 | 21.237 | 6.573 | 5.449 |
| TCGA-EJ-7794 | 0.781 | 0 | 3.145 | 30.610 | 5.243 | 3.813 |
| TCGA-HI-7168 | 0.844 | 1 | 2.223 | 26.203 | 4.040 | 2.654 |
| TCGA-V1-A8X3 | 0.553 | 0 | 2.564 | 23.512 | 5.289 | 4.679 |
| TCGA-EJ-8472 | 0.537 | 1 | 2.393 | 23.230 | 5.792 | 4.423 |
| TCGA-CH-5739 | 1.838 | 0 | 2.934 | 15.631 | 4.342 | 6.948 |
| TCGA-EJ-5525 | 0.926 | 0 | 2.757 | 16.497 | 3.057 | 1.711 |
| TCGA-XK-AAJU | 3.304 | 0 | 3.043 | 44.824 | 6.502 | 7.009 |
| TCGA-EJ-5530 | 3.485 | 0 | 3.015 | 32.753 | 5.506 | 2.542 |
| TCGA-M7-A720 | 0.827 | 0 | 2.710 | 39.343 | 6.344 | 3.774 |
| TCGA-EJ-5522 | 3.844 | 0 | 3.008 | 28.318 | 5.448 | 5.273 |
| TCGA-KC-A4BL | 0.529 | 1 | 2.888 | 41.414 | 6.560 | 11.344 |
| TCGA-YL-A9WX | 4.126 | 1 | 2.849 | 34.476 | 6.471 | 10.311 |
| TCGA-YL-A8SQ | 0.901 | 1 | 2.009 | 26.217 | 5.219 | 3.839 |
| TCGA-EJ-A46H | 0.627 | 0 | 2.811 | 35.798 | 6.525 | 6.723 |
| TCGA-HC-7212 | 0.112 | 1 | 2.827 | 17.080 | 5.083 | 2.331 |
| TCGA-ZG-A9LZ | 0.849 | 0 | 2.666 | 20.762 | 4.700 | 8.306 |
| TCGA-HC-A632 | 0.167 | 0 | 2.434 | 20.399 | 4.424 | 4.148 |
| TCGA-KC-A4BV | 3.638 | 1 | 2.586 | 22.936 | 6.094 | 9.249 |
| TCGA-ZG-A8QY | 0.093 | 0 | 2.749 | 39.335 | 4.955 | 2.072 |
| TCGA-EJ-5507 | 0.499 | 0 | 3.275 | 31.392 | 6.373 | 8.172 |
| TCGA-ZG-A9KY | 0.093 | 0 | 2.447 | 18.333 | 6.211 | 15.490 |
| TCGA-HC-A76W | 0.584 | 0 | 2.519 | 33.716 | 5.295 | 7.171 |
| TCGA-EJ-7123 | 4.085 | 0 | 3.019 | 29.617 | 4.831 | 2.539 |
| TCGA-MG-AAMC | 0.474 | 0 | 1.375 | 21.640 | 4.809 | 2.588 |
| TCGA-EJ-8470 | 2.249 | 0 | 3.141 | 20.168 | 6.576 | 18.234 |
| TCGA-XK-AAJA | 2.548 | 0 | 1.938 | 52.547 | 6.112 | 3.674 |
| TCGA-V1-A9ZI | 0.236 | 1 | 2.250 | 26.769 | 3.775 | 2.746 |
| TCGA-EJ-7318 | 0.518 | 0 | 2.737 | 28.273 | 4.240 | 7.476 |
| TCGA-HC-7080 | 0.285 | 0 | 3.200 | 45.521 | 6.840 | 3.771 |
| TCGA-V1-A9Z9 | 0.808 | 0 | 3.243 | 46.326 | 5.532 | 2.961 |
| TCGA-V1-A8WV | 1.879 | 0 | 2.118 | 27.776 | 5.999 | 8.944 |
| TCGA-HC-8216 | 0.115 | 0 | 2.648 | 22.158 | 5.151 | 7.171 |
| TCGA-YL-A8SC | 3.455 | 1 | 2.613 | 18.187 | 5.742 | 8.236 |
| TCGA-HI-7171 | 0.597 | 1 | 2.204 | 20.300 | 3.975 | 1.591 |
| TCGA-KK-A6E0 | 2.578 | 1 | 3.038 | 39.845 | 4.428 | 2.946 |
| TCGA-V1-A8WS | 1.299 | 1 | 1.999 | 31.694 | 7.483 | 2.873 |
| TCGA-HC-7077 | 0.211 | 0 | 3.081 | 39.887 | 5.772 | 2.091 |
| TCGA-KK-A8I6 | 1.830 | 0 | 2.803 | 36.377 | 5.816 | 2.761 |
| TCGA-J4-A83I | 0.764 | 0 | 2.681 | 31.235 | 6.799 | 6.714 |
| TCGA-CH-5791 | 2.751 | 0 | 3.076 | 15.743 | 4.287 | 5.301 |
| TCGA-EJ-A7NM | 0.351 | 0 | 2.089 | 28.813 | 6.018 | 9.819 |
| TCGA-YL-A8SJ | 2.060 | 1 | 2.333 | 27.792 | 6.348 | 3.148 |
| TCGA-EJ-5508 | 2.485 | 0 | 2.993 | 20.647 | 4.740 | 4.152 |
| TCGA-HC-A8D0 | 0.301 | 0 | 2.836 | 33.703 | 5.198 | 4.974 |
| TCGA-J4-A6M7 | 0.764 | 0 | 2.917 | 35.298 | 5.902 | 1.918 |

| TCGA-EJ-5501 | 1.997 | 0 | 3.133 | 25.955 | 5.491 | 4.146 |
| --- | --- | --- | --- | --- | --- | --- |
| TCGA-EJ-5511 | 2.159 | 0 | 2.679 | 22.471 | 4.972 | 3.500 |
| TCGA-HC-7744 | 0.140 | 0 | 2.938 | 36.306 | 6.420 | 2.994 |
| TCGA-G9-A9S7 | 1.762 | 0 | 2.896 | 27.609 | 5.396 | 3.867 |
| TCGA-EJ-5542 | 1.422 | 0 | 3.155 | 21.430 | 4.522 | 2.634 |
| TCGA-KK-A8IM | 3.940 | 0 | 2.645 | 23.144 | 5.576 | 6.086 |
| TCGA-YL-A8SH | 2.397 | 0 | 2.634 | 45.491 | 5.953 | 2.730 |
| TCGA-EJ-8469 | 5.274 | 1 | 2.718 | 17.551 | 4.771 | 3.291 |
| TCGA-2A-A8W3 | 0.542 | 1 | 2.599 | 31.760 | 5.125 | 2.558 |
| TCGA-V1-A9ZR | 0.485 | 1 | 3.060 | 23.436 | 4.848 | 6.527 |
| TCGA-VP-A87D | 3.271 | 1 | 2.459 | 27.172 | 5.798 | 2.426 |
| TCGA-EJ-A7NN | 0.540 | 1 | 3.211 | 52.269 | 8.023 | 6.564 |
| TCGA-EJ-7327 | 0.479 | 0 | 3.125 | 31.746 | 6.720 | 3.433 |
| TCGA-HC-7736 | 0.060 | 0 | 2.958 | 18.861 | 6.126 | 10.183 |
| TCGA-CH-5788 | 2.153 | 0 | 2.636 | 14.231 | 3.309 | 1.751 |
| TCGA-XK-AAJP | 2.436 | 0 | 3.006 | 22.935 | 4.731 | 3.106 |
| TCGA-XJ-A9DK | 0.753 | 0 | 2.528 | 40.984 | 6.501 | 3.039 |
| TCGA-EJ-A7NH | 0.471 | 0 | 2.658 | 40.571 | 7.044 | 2.404 |
| TCGA-VN-A88N | 0.482 | 0 | 2.272 | 23.915 | 5.507 | 3.570 |
| TCGA-CH-5790 | 2.668 | 0 | 2.752 | 16.136 | 4.819 | 3.499 |
| TCGA-KK-A6E2 | 12.614 | 0 | 2.404 | 20.783 | 5.862 | 3.921 |
| TCGA-M7-A71Z | 0.573 | 0 | 2.256 | 42.715 | 5.431 | 1.782 |
| TCGA-G9-A9S0 | 1.175 | 0 | 3.516 | 50.232 | 6.345 | 8.586 |
| TCGA-CH-5765 | 1.918 | 0 | 2.801 | 31.537 | 6.129 | 2.530 |
| TCGA-HC-8260 | 0.290 | 0 | 2.684 | 38.891 | 7.001 | 3.363 |
| TCGA-KK-A8I8 | 2.121 | 0 | 2.325 | 17.199 | 5.104 | 3.216 |
| TCGA-HC-A6HX | 0.104 | 0 | 3.198 | 55.087 | 3.461 | 5.884 |
| TCGA-V1-A9Z7 | 0.337 | 1 | 3.246 | 25.189 | 3.323 | 2.947 |
| TCGA-HC-7211 | 0.066 | 0 | 3.132 | 15.539 | 5.149 | 8.673 |
| TCGA-XK-AAIV | 2.625 | 0 | 4.483 | 44.182 | 5.027 | 8.961 |
| TCGA-SU-A7E7 | 0.307 | 0 | 2.813 | 30.207 | 6.833 | 10.594 |
| TCGA-EJ-5518 | 3.274 | 0 | 4.399 | 38.064 | 4.647 | 4.163 |
| TCGA-XK-AAIR | 2.663 | 0 | 3.144 | 25.404 | 5.236 | 4.670 |
| TCGA-M7-A724 | 1.323 | 0 | 2.864 | 27.553 | 4.800 | 1.849 |
| TCGA-V1-A9O5 | 0.340 | 1 | 1.944 | 29.630 | 7.472 | 9.187 |
| TCGA-KK-A7AP | 0.537 | 0 | 2.594 | 22.766 | 5.350 | 2.145 |
| TCGA-HC-A6AN | 0.134 | 0 | 3.877 | 35.304 | 3.991 | 2.901 |
| TCGA-M7-A71Y | 0.433 | 0 | 3.247 | 39.766 | 5.477 | 4.910 |
| TCGA-V1-A9ZK | 2.874 | 0 | 3.690 | 30.055 | 5.161 | 3.887 |
| TCGA-KK-A8IA | 3.942 | 0 | 2.768 | 26.167 | 5.930 | 2.610 |
| TCGA-EJ-7783 | 0.222 | 0 | 3.115 | 23.526 | 5.882 | 3.354 |
| TCGA-ZG-A9L0 | 0.186 | 0 | 3.047 | 26.735 | 6.047 | 8.893 |
| TCGA-EJ-A46E | 1.436 | 0 | 2.515 | 36.774 | 6.840 | 10.339 |
| TCGA-KK-A8II | 1.715 | 1 | 2.821 | 29.494 | 6.671 | 4.412 |
| TCGA-YL-A8SA | 1.403 | 0 | 2.575 | 23.799 | 7.185 | 4.951 |
| TCGA-EJ-7789 | 0.192 | 0 | 2.489 | 18.788 | 5.919 | 1.739 |
| TCGA-J9-A8CM | 0.942 | 1 | 2.226 | 25.358 | 7.464 | 2.515 |
| TCGA-EJ-7325 | 1.460 | 0 | 2.515 | 22.422 | 2.913 | 1.348 |
| TCGA-HC-8258 | 0.181 | 0 | 4.753 | 3.915 | 3.210 | 13.048 |
| TCGA-HC-A6AP | 0.195 | 0 | 4.325 | 50.964 | 3.582 | 4.143 |
| TCGA-YL-A9WY | 2.096 | 1 | 3.146 | 32.018 | 5.581 | 9.113 |

| TCGA-XK-AAJR | 0.359 | 1 | 2.693 | 24.708 | 6.222 | 2.011 |
| --- | --- | --- | --- | --- | --- | --- |
| TCGA-YL-A8SO | 8.896 | 0 | 2.401 | 25.165 | 4.211 | 3.970 |
| TCGA-XJ-A9DI | 4.003 | 0 | 3.029 | 32.361 | 7.122 | 11.977 |
| TCGA-XK-AAIW | 2.663 | 0 | 2.252 | 41.094 | 6.891 | 3.269 |
| TCGA-EJ-8468 | 5.373 | 0 | 3.412 | 26.659 | 5.786 | 3.416 |
| TCGA-EJ-A46F | 0.589 | 1 | 2.316 | 22.574 | 4.757 | 2.181 |
| TCGA-YL-A8SR | 0.584 | 0 | 3.270 | 24.830 | 5.360 | 2.430 |
| TCGA-TP-A8TV | 0.682 | 0 | 3.281 | 50.392 | 5.587 | 2.316 |
| TCGA-QU-A6IO | 3.934 | 0 | 3.534 | 53.940 | 3.554 | 5.349 |
| TCGA-EJ-5519 | 0.230 | 0 | 3.190 | 34.392 | 7.125 | 2.530 |
| TCGA-V1-A9OT | 1.304 | 1 | 1.402 | 27.881 | 6.125 | 2.776 |
| TCGA-V1-A9OL | 0.359 | 1 | 2.904 | 33.149 | 6.772 | 5.399 |
| TCGA-HC-7230 | 0.140 | 0 | 2.877 | 41.232 | 8.272 | 3.401 |
| TCGA-EJ-5514 | 1.984 | 0 | 3.779 | 21.048 | 4.961 | 4.535 |
| TCGA-YL-A8S8 | 1.860 | 1 | 3.372 | 31.085 | 4.640 | 4.027 |
| TCGA-VP-A87K | 1.296 | 1 | 2.687 | 21.387 | 6.470 | 4.691 |
| TCGA-FC-A6HD | 0.148 | 0 | 3.437 | 31.045 | 3.116 | 1.894 |
| TCGA-HC-A4ZV | 0.063 | 0 | 2.136 | 21.822 | 6.704 | 3.992 |
| TCGA-EJ-A65F | 0.205 | 1 | 2.909 | 20.294 | 5.428 | 3.298 |
| TCGA-YL-A8HK | 3.770 | 1 | 3.109 | 24.822 | 6.272 | 4.256 |
| TCGA-ZG-A9L5 | 2.340 | 0 | 2.481 | 17.744 | 6.103 | 2.302 |
| TCGA-YL-A8SL | 2.186 | 0 | 3.441 | 34.080 | 6.490 | 3.247 |
| TCGA-FC-7961 | 0.192 | 0 | 3.905 | 31.075 | 4.209 | 3.226 |
| TCGA-CH-5752 | 2.584 | 0 | 2.953 | 21.361 | 7.292 | 2.162 |
| TCGA-YL-A9WL | 2.027 | 1 | 2.621 | 19.017 | 7.610 | 2.349 |
| TCGA-YL-A8SP | 5.945 | 1 | 2.236 | 12.646 | 6.241 | 0.970 |
| TCGA-KK-A7B4 | 1.745 | 1 | 3.040 | 43.145 | 8.434 | 3.716 |
| TCGA-EJ-A65D | 1.077 | 0 | 3.042 | 23.809 | 4.652 | 0.862 |
| TCGA-HC-7213 | 0.466 | 1 | 3.393 | 16.580 | 6.688 | 6.347 |
| TCGA-2A-A8VT | 2.726 | 0 | 2.865 | 23.068 | 8.319 | 5.239 |
| TCGA-YL-A8HL | 3.214 | 0 | 2.640 | 17.090 | 7.755 | 1.771 |
| TCGA-Y6-A9XI | 1.441 | 0 | 2.575 | 26.101 | 7.814 | 2.641 |
| TCGA-CH-5761 | 0.077 | 0 | 4.139 | 23.817 | 4.796 | 2.222 |
| TCGA-HC-A9TE | 0.367 | 1 | 2.526 | 30.439 | 8.196 | 9.282 |
| TCGA-KK-A7AU | 0.567 | 1 | 2.620 | 21.251 | 8.956 | 3.885 |
| TCGA-HC-A48F | 0.126 | 0 | 2.604 | 21.687 | 8.713 | 0.892 |
| TCGA-J4-A67N | 1.211 | 1 | 3.771 | 30.637 | 4.713 | 6.452 |
| TCGA-HC-7740 | 0.145 | 0 | 5.859 | 7.796 | 6.447 | 14.452 |
| TCGA-XQ-A8TA | 0.400 | 0 | 2.539 | 38.979 | 8.570 | 1.036 |

| IRF3 | ZDHHC1 | RiskScore | Risk |
| --- | --- | --- | --- |
| 17.626 | 7.001 | -1.90E+00 | LOW |
| 14.609 | 3.386 | -1.34E+00 | LOW |
| 12.597 | 5.316 | -1.25E+00 | LOW |
| 17.672 | 5.614 | -1.17E+00 | LOW |
| 14.491 | 3.524 | -1.14E+00 | LOW |
| 8.551 | 11.491 | -1.12E+00 | LOW |
| 20.005 | 7.845 | -9.57E-01 | LOW |
| 8.887 | 5.450 | -9.18E-01 | LOW |
| 9.468 | 4.897 | -5.81E-01 | LOW |
| 14.908 | 7.504 | -3.85E-01 | LOW |
| 8.244 | 4.441 | -3.30E-01 | LOW |
| 15.765 | 6.086 | -3.30E-01 | LOW |
| 11.731 | 5.061 | -2.49E-01 | LOW |
| 10.107 | 6.028 | -1.37E-01 | LOW |
| 8.493 | 3.820 | -1.23E-01 | LOW |
| 13.682 | 5.870 | -1.15E-01 | LOW |
| 16.744 | 4.106 | -5.60E-02 | LOW |
| 14.545 | 6.461 | -2.11E-02 | LOW |
| 13.503 | 5.250 | 1.88E-02 | LOW |
| 11.105 | 2.761 | 4.83E-02 | LOW |
| 26.542 | 6.580 | 7.76E-02 | LOW |
| 7.868 | 4.549 | 1.17E-01 | LOW |
| 19.784 | 5.064 | 1.27E-01 | LOW |
| 12.160 | 3.463 | 1.41E-01 | LOW |
| 9.562 | 2.478 | 1.88E-01 | LOW |
| 9.985 | 5.887 | 2.49E-01 | LOW |
| 4.797 | 4.090 | 2.98E-01 | LOW |
| 15.366 | 7.168 | 3.11E-01 | LOW |
| 11.075 | 8.884 | 3.32E-01 | LOW |
| 15.947 | 4.626 | 3.53E-01 | LOW |
| 14.046 | 6.143 | 3.87E-01 | LOW |
| 13.058 | 4.480 | 4.07E-01 | LOW |
| 15.666 | 2.960 | 4.10E-01 | LOW |
| 7.781 | 4.679 | 4.17E-01 | LOW |
| 12.496 | 7.640 | 4.21E-01 | LOW |
| 10.012 | 5.729 | 4.37E-01 | LOW |
| 15.154 | 2.137 | 4.39E-01 | LOW |
| 25.529 | 7.291 | 4.64E-01 | LOW |
| 9.928 | 5.982 | 4.94E-01 | LOW |
| 13.441 | 7.213 | 5.14E-01 | LOW |
| 19.241 | 4.652 | 5.32E-01 | LOW |
| 11.378 | 5.988 | 5.82E-01 | LOW |
| 12.119 | 5.473 | 5.92E-01 | LOW |
| 10.105 | 2.989 | 5.98E-01 | LOW |
| 13.551 | 1.205 | 6.01E-01 | LOW |
| 10.468 | 2.586 | 6.22E-01 | LOW |
| 10.653 | 4.701 | 6.24E-01 | LOW |
| 13.296 | 6.089 | 6.42E-01 | LOW |

| 11.095 | 4.576 | 6.86E-01 | LOW |
| --- | --- | --- | --- |
| 10.763 | 6.398 | 7.07E-01 | LOW |
| 8.894 | 4.211 | 7.38E-01 | LOW |
| 9.287 | 4.331 | 7.46E-01 | LOW |
| 10.498 | 5.725 | 7.47E-01 | LOW |
| 18.642 | 10.484 | 7.55E-01 | LOW |
| 18.595 | 3.674 | 7.60E-01 | LOW |
| 16.461 | 7.168 | 7.64E-01 | LOW |
| 9.555 | 5.651 | 7.89E-01 | LOW |
| 23.024 | 8.581 | 8.07E-01 | LOW |
| 8.056 | 7.334 | 8.18E-01 | LOW |
| 7.010 | 2.273 | 8.21E-01 | LOW |
| 11.702 | 2.286 | 8.36E-01 | LOW |
| 19.953 | 10.714 | 8.43E-01 | LOW |
| 10.792 | 5.877 | 8.73E-01 | LOW |
| 6.640 | 2.506 | 9.14E-01 | LOW |
| 9.250 | 1.787 | 9.17E-01 | LOW |
| 9.971 | 2.907 | 9.24E-01 | LOW |
| 8.986 | 6.646 | 9.29E-01 | LOW |
| 5.416 | 6.606 | 9.65E-01 | LOW |
| 29.968 | 9.674 | 1.00E+00 | LOW |
| 8.245 | 3.460 | 1.00E+00 | LOW |
| 20.421 | 4.014 | 1.02E+00 | LOW |
| 17.166 | 7.909 | 1.02E+00 | LOW |
| 9.219 | 4.790 | 1.03E+00 | LOW |
| 16.281 | 6.508 | 1.03E+00 | LOW |
| 15.472 | 7.908 | 1.04E+00 | LOW |
| 10.570 | 5.943 | 1.04E+00 | LOW |
| 21.837 | 6.327 | 1.05E+00 | LOW |
| 16.267 | 5.879 | 1.05E+00 | LOW |
| 8.151 | 4.913 | 1.05E+00 | LOW |
| 11.631 | 4.084 | 1.07E+00 | LOW |
| 12.142 | 6.874 | 1.07E+00 | LOW |
| 9.731 | 6.108 | 1.07E+00 | LOW |
| 12.342 | 0.509 | 1.08E+00 | LOW |
| 8.269 | 2.379 | 1.09E+00 | LOW |
| 7.056 | 1.298 | 1.09E+00 | LOW |
| 9.403 | 3.841 | 1.10E+00 | LOW |
| 8.998 | 4.311 | 1.10E+00 | LOW |
| 10.346 | 6.756 | 1.11E+00 | LOW |
| 20.209 | 4.027 | 1.12E+00 | LOW |
| 7.503 | 3.569 | 1.13E+00 | LOW |
| 19.106 | 11.810 | 1.15E+00 | LOW |
| 20.693 | 7.696 | 1.15E+00 | LOW |
| 10.459 | 4.682 | 1.17E+00 | LOW |
| 18.763 | 7.022 | 1.17E+00 | LOW |
| 15.736 | 4.974 | 1.17E+00 | LOW |
| 14.153 | 4.994 | 1.18E+00 | LOW |
| 8.963 | 6.038 | 1.19E+00 | LOW |
| 14.014 | 7.328 | 1.19E+00 | LOW |
| 11.502 | 7.556 | 1.20E+00 | LOW |

| 17.634 | 6.803 | 1.21E+00 | LOW |
| --- | --- | --- | --- |
| 10.173 | 7.971 | 1.21E+00 | LOW |
| 9.868 | 2.129 | 1.21E+00 | LOW |
| 17.661 | 9.427 | 1.21E+00 | LOW |
| 18.267 | 5.545 | 1.23E+00 | LOW |
| 9.821 | 5.304 | 1.24E+00 | LOW |
| 6.831 | 2.843 | 1.26E+00 | LOW |
| 9.031 | 5.669 | 1.26E+00 | LOW |
| 8.806 | 5.887 | 1.27E+00 | LOW |
| 17.252 | 7.874 | 1.28E+00 | LOW |
| 25.233 | 2.238 | 1.29E+00 | LOW |
| 10.308 | 3.368 | 1.29E+00 | LOW |
| 10.541 | 7.141 | 1.30E+00 | LOW |
| 10.346 | 2.664 | 1.31E+00 | LOW |
| 24.224 | 7.633 | 1.31E+00 | LOW |
| 18.918 | 6.927 | 1.32E+00 | LOW |
| 17.663 | 4.916 | 1.32E+00 | LOW |
| 10.805 | 5.383 | 1.33E+00 | LOW |
| 10.431 | 5.750 | 1.34E+00 | LOW |
| 10.535 | 7.397 | 1.34E+00 | LOW |
| 17.891 | 5.888 | 1.35E+00 | LOW |
| 11.577 | 4.040 | 1.36E+00 | LOW |
| 10.318 | 4.635 | 1.37E+00 | LOW |
| 22.058 | 7.325 | 1.37E+00 | LOW |
| 14.406 | 6.837 | 1.37E+00 | LOW |
| 15.440 | 4.327 | 1.37E+00 | LOW |
| 14.422 | 7.882 | 1.37E+00 | LOW |
| 10.062 | 5.010 | 1.38E+00 | LOW |
| 12.827 | 4.307 | 1.38E+00 | LOW |
| 10.332 | 4.170 | 1.39E+00 | LOW |
| 13.002 | 3.361 | 1.39E+00 | LOW |
| 14.343 | 7.447 | 1.40E+00 | LOW |
| 22.051 | 5.567 | 1.40E+00 | LOW |
| 6.222 | 2.262 | 1.40E+00 | LOW |
| 6.639 | 5.844 | 1.40E+00 | LOW |
| 16.559 | 5.639 | 1.40E+00 | LOW |
| 9.152 | 4.045 | 1.41E+00 | LOW |
| 7.677 | 5.410 | 1.41E+00 | LOW |
| 16.021 | 5.666 | 1.42E+00 | LOW |
| 9.926 | 4.725 | 1.42E+00 | LOW |
| 9.338 | 5.696 | 1.42E+00 | LOW |
| 13.018 | 6.808 | 1.43E+00 | LOW |
| 12.583 | 0.950 | 1.43E+00 | LOW |
| 20.905 | 6.573 | 1.43E+00 | LOW |
| 10.384 | 4.717 | 1.43E+00 | LOW |
| 22.847 | 7.370 | 1.43E+00 | LOW |
| 38.907 | 7.336 | 1.44E+00 | LOW |
| 7.311 | 5.202 | 1.44E+00 | LOW |
| 9.670 | 6.518 | 1.45E+00 | LOW |
| 19.191 | 6.535 | 1.46E+00 | LOW |
| 6.961 | 4.125 | 1.47E+00 | LOW |

| 7.742 | 3.753 | 1.49E+00 | LOW |
| --- | --- | --- | --- |
| 7.057 | 4.105 | 1.49E+00 | LOW |
| 14.898 | 5.818 | 1.49E+00 | LOW |
| 11.515 | 5.068 | 1.50E+00 | LOW |
| 6.418 | 3.957 | 1.51E+00 | LOW |
| 12.929 | 5.238 | 1.51E+00 | LOW |
| 21.876 | 4.372 | 1.53E+00 | LOW |
| 6.624 | 4.005 | 1.54E+00 | LOW |
| 13.289 | 4.597 | 1.54E+00 | LOW |
| 15.345 | 5.635 | 1.54E+00 | LOW |
| 11.620 | 1.013 | 1.54E+00 | LOW |
| 14.257 | 5.751 | 1.55E+00 | LOW |
| 9.213 | 4.013 | 1.56E+00 | LOW |
| 23.592 | 7.695 | 1.56E+00 | LOW |
| 8.343 | 5.690 | 1.57E+00 | LOW |
| 15.283 | 8.049 | 1.57E+00 | LOW |
| 12.051 | 4.299 | 1.57E+00 | LOW |
| 14.932 | 3.998 | 1.57E+00 | LOW |
| 12.705 | 6.140 | 1.57E+00 | LOW |
| 7.843 | 6.583 | 1.58E+00 | LOW |
| 24.908 | 3.104 | 1.58E+00 | LOW |
| 25.688 | 8.184 | 1.59E+00 | LOW |
| 8.850 | 6.797 | 1.59E+00 | LOW |
| 6.867 | 3.294 | 1.60E+00 | LOW |
| 15.136 | 7.618 | 1.60E+00 | LOW |
| 8.479 | 5.243 | 1.61E+00 | LOW |
| 9.399 | 5.033 | 1.61E+00 | LOW |
| 16.926 | 4.993 | 1.61E+00 | LOW |
| 11.791 | 7.314 | 1.61E+00 | LOW |
| 20.898 | 6.451 | 1.62E+00 | LOW |
| 18.138 | 6.544 | 1.62E+00 | LOW |
| 10.351 | 4.185 | 1.62E+00 | LOW |
| 28.612 | 9.795 | 1.64E+00 | LOW |
| 8.466 | 3.010 | 1.64E+00 | LOW |
| 7.122 | 4.670 | 1.65E+00 | LOW |
| 9.631 | 4.799 | 1.66E+00 | LOW |
| 8.505 | 4.913 | 1.67E+00 | LOW |
| 9.347 | 5.981 | 1.68E+00 | LOW |
| 14.067 | 4.105 | 1.68E+00 | LOW |
| 7.097 | 4.595 | 1.68E+00 | LOW |
| 24.093 | 7.077 | 1.69E+00 | LOW |
| 26.962 | 4.804 | 1.70E+00 | LOW |
| 11.679 | 6.142 | 1.71E+00 | LOW |
| 12.929 | 6.626 | 1.71E+00 | LOW |
| 11.139 | 4.196 | 1.71E+00 | LOW |
| 23.215 | 7.707 | 1.72E+00 | LOW |
| 5.797 | 2.371 | 1.72E+00 | LOW |
| 19.449 | 7.336 | 1.73E+00 | LOW |
| 14.760 | 7.263 | 1.75E+00 | LOW |
| 23.341 | 6.085 | 1.75E+00 | LOW |
| 14.200 | 4.588 | 1.76E+00 | LOW |

| 16.236 | 3.921 | 1.76E+00 | LOW |
| --- | --- | --- | --- |
| 8.709 | 5.287 | 1.76E+00 | LOW |
| 8.022 | 6.557 | 1.77E+00 | LOW |
| 19.613 | 7.803 | 1.78E+00 | LOW |
| 17.319 | 1.306 | 1.78E+00 | LOW |
| 19.524 | 6.315 | 1.79E+00 | LOW |
| 15.093 | 7.364 | 1.79E+00 | LOW |
| 13.699 | 5.744 | 1.79E+00 | LOW |
| 11.666 | 4.278 | 1.79E+00 | LOW |
| 9.290 | 2.938 | 1.79E+00 | LOW |
| 14.891 | 7.077 | 1.79E+00 | LOW |
| 8.243 | 3.979 | 1.79E+00 | LOW |
| 14.326 | 4.352 | 1.80E+00 | LOW |
| 11.507 | 6.440 | 1.80E+00 | LOW |
| 18.475 | 7.476 | 1.82E+00 | LOW |
| 10.844 | 5.646 | 1.83E+00 | LOW |
| 6.529 | 4.789 | 1.83E+00 | LOW |
| 10.284 | 6.171 | 1.83E+00 | LOW |
| 15.859 | 5.867 | 1.83E+00 | LOW |
| 12.301 | 5.707 | 1.84E+00 | LOW |
| 29.201 | 9.788 | 1.84E+00 | LOW |
| 7.529 | 6.602 | 1.84E+00 | LOW |
| 9.134 | 2.565 | 1.84E+00 | LOW |
| 6.342 | 5.153 | 1.85E+00 | LOW |
| 10.143 | 5.500 | 1.86E+00 | LOW |
| 25.325 | 5.139 | 1.86E+00 | LOW |
| 18.650 | 6.996 | 1.87E+00 | LOW |
| 13.371 | 3.352 | 1.87E+00 | LOW |
| 12.528 | 5.556 | 1.87E+00 | LOW |
| 18.950 | 5.161 | 1.87E+00 | LOW |
| 10.801 | 4.544 | 1.87E+00 | LOW |
| 9.441 | 5.642 | 1.88E+00 | LOW |
| 16.622 | 5.827 | 1.88E+00 | LOW |
| 8.143 | 4.090 | 1.88E+00 | LOW |
| 16.490 | 7.843 | 1.89E+00 | LOW |
| 26.330 | 7.611 | 1.90E+00 | LOW |
| 9.737 | 6.798 | 1.90E+00 | LOW |
| 17.477 | 7.783 | 1.91E+00 | LOW |
| 16.045 | 7.134 | 1.91E+00 | LOW |
| 8.983 | 3.759 | 1.91E+00 | LOW |
| 4.770 | 3.039 | 1.92E+00 | LOW |
| 13.999 | 6.642 | 1.92E+00 | LOW |
| 18.069 | 5.420 | 1.92E+00 | LOW |
| 8.901 | 3.055 | 1.92E+00 | LOW |
| 20.593 | 4.025 | 1.93E+00 | LOW |
| 15.421 | 5.677 | 1.93E+00 | LOW |
| 13.193 | 6.537 | 1.93E+00 | HIGH |
| 7.855 | 1.563 | 1.95E+00 | HIGH |
| 7.771 | 3.058 | 1.95E+00 | HIGH |
| 21.143 | 4.995 | 1.95E+00 | HIGH |
| 8.421 | 3.107 | 1.96E+00 | HIGH |

| 15.062 | 6.747 | 1.97E+00 | HIGH |
| --- | --- | --- | --- |
| 9.557 | 2.646 | 1.97E+00 | HIGH |
| 10.131 | 4.335 | 1.98E+00 | HIGH |
| 11.861 | 5.778 | 1.98E+00 | HIGH |
| 11.502 | 5.138 | 1.98E+00 | HIGH |
| 11.450 | 5.217 | 1.98E+00 | HIGH |
| 14.030 | 4.398 | 1.99E+00 | HIGH |
| 7.655 | 4.221 | 1.99E+00 | HIGH |
| 25.028 | 7.284 | 1.99E+00 | HIGH |
| 7.172 | 3.328 | 2.00E+00 | HIGH |
| 15.472 | 6.818 | 2.00E+00 | HIGH |
| 15.276 | 4.086 | 2.00E+00 | HIGH |
| 15.349 | 6.917 | 2.01E+00 | HIGH |
| 9.895 | 6.040 | 2.02E+00 | HIGH |
| 13.948 | 0.831 | 2.02E+00 | HIGH |
| 19.408 | 3.331 | 2.02E+00 | HIGH |
| 39.960 | 8.143 | 2.03E+00 | HIGH |
| 21.788 | 7.531 | 2.03E+00 | HIGH |
| 8.271 | 4.308 | 2.04E+00 | HIGH |
| 15.526 | 9.297 | 2.05E+00 | HIGH |
| 10.123 | 4.294 | 2.06E+00 | HIGH |
| 10.919 | 3.790 | 2.07E+00 | HIGH |
| 5.648 | 2.323 | 2.09E+00 | HIGH |
| 14.836 | 7.102 | 2.09E+00 | HIGH |
| 7.936 | 4.091 | 2.10E+00 | HIGH |
| 15.415 | 4.340 | 2.10E+00 | HIGH |
| 10.381 | 2.260 | 2.10E+00 | HIGH |
| 16.339 | 5.114 | 2.11E+00 | HIGH |
| 13.356 | 2.652 | 2.11E+00 | HIGH |
| 11.567 | 4.644 | 2.11E+00 | HIGH |
| 10.766 | 3.892 | 2.12E+00 | HIGH |
| 7.422 | 4.656 | 2.12E+00 | HIGH |
| 8.959 | 2.596 | 2.13E+00 | HIGH |
| 12.901 | 4.206 | 2.14E+00 | HIGH |
| 17.477 | 7.440 | 2.14E+00 | HIGH |
| 11.272 | 4.874 | 2.14E+00 | HIGH |
| 14.617 | 5.685 | 2.14E+00 | HIGH |
| 13.399 | 4.553 | 2.14E+00 | HIGH |
| 14.603 | 7.009 | 2.15E+00 | HIGH |
| 7.849 | 3.192 | 2.16E+00 | HIGH |
| 8.174 | 3.345 | 2.16E+00 | HIGH |
| 12.890 | 3.665 | 2.16E+00 | HIGH |
| 27.676 | 7.765 | 2.17E+00 | HIGH |
| 7.557 | 3.685 | 2.17E+00 | HIGH |
| 16.606 | 4.129 | 2.17E+00 | HIGH |
| 8.097 | 4.997 | 2.18E+00 | HIGH |
| 18.216 | 1.065 | 2.18E+00 | HIGH |
| 7.517 | 5.259 | 2.19E+00 | HIGH |
| 6.762 | 2.989 | 2.19E+00 | HIGH |
| 18.507 | 9.706 | 2.19E+00 | HIGH |
| 15.778 | 6.491 | 2.19E+00 | HIGH |

| 14.391 | 6.897 | 2.19E+00 | HIGH |
| --- | --- | --- | --- |
| 12.201 | 2.220 | 2.19E+00 | HIGH |
| 15.352 | 8.328 | 2.20E+00 | HIGH |
| 11.839 | 3.540 | 2.21E+00 | HIGH |
| 15.603 | 5.668 | 2.22E+00 | HIGH |
| 7.346 | 3.500 | 2.22E+00 | HIGH |
| 10.313 | 4.851 | 2.22E+00 | HIGH |
| 17.789 | 3.977 | 2.24E+00 | HIGH |
| 9.763 | 2.776 | 2.24E+00 | HIGH |
| 13.012 | 3.566 | 2.27E+00 | HIGH |
| 9.715 | 5.008 | 2.27E+00 | HIGH |
| 17.648 | 8.760 | 2.27E+00 | HIGH |
| 11.650 | 2.587 | 2.28E+00 | HIGH |
| 9.012 | 1.351 | 2.29E+00 | HIGH |
| 8.420 | 3.553 | 2.30E+00 | HIGH |
| 10.093 | 5.152 | 2.30E+00 | HIGH |
| 16.027 | 0.598 | 2.30E+00 | HIGH |
| 16.170 | 5.476 | 2.31E+00 | HIGH |
| 10.670 | 4.333 | 2.31E+00 | HIGH |
| 18.371 | 1.065 | 2.32E+00 | HIGH |
| 6.108 | 4.916 | 2.32E+00 | HIGH |
| 9.352 | 5.474 | 2.32E+00 | HIGH |
| 13.206 | 4.577 | 2.33E+00 | HIGH |
| 13.420 | 6.401 | 2.33E+00 | HIGH |
| 27.487 | 8.199 | 2.34E+00 | HIGH |
| 20.722 | 8.911 | 2.34E+00 | HIGH |
| 16.188 | 5.523 | 2.35E+00 | HIGH |
| 15.390 | 7.959 | 2.35E+00 | HIGH |
| 10.017 | 3.463 | 2.35E+00 | HIGH |
| 14.214 | 3.677 | 2.37E+00 | HIGH |
| 9.947 | 4.392 | 2.37E+00 | HIGH |
| 16.919 | 3.894 | 2.37E+00 | HIGH |
| 21.823 | 5.213 | 2.37E+00 | HIGH |
| 6.527 | 3.903 | 2.38E+00 | HIGH |
| 19.225 | 6.433 | 2.38E+00 | HIGH |
| 20.201 | 6.212 | 2.38E+00 | HIGH |
| 12.668 | 4.118 | 2.40E+00 | HIGH |
| 17.366 | 7.530 | 2.41E+00 | HIGH |
| 7.493 | 5.412 | 2.42E+00 | HIGH |
| 13.229 | 4.596 | 2.43E+00 | HIGH |
| 8.185 | 3.169 | 2.44E+00 | HIGH |
| 16.839 | 3.684 | 2.45E+00 | HIGH |
| 16.363 | 5.145 | 2.46E+00 | HIGH |
| 8.737 | 3.220 | 2.46E+00 | HIGH |
| 12.024 | 1.340 | 2.47E+00 | HIGH |
| 10.653 | 4.168 | 2.47E+00 | HIGH |
| 9.716 | 3.796 | 2.47E+00 | HIGH |
| 12.571 | 4.489 | 2.47E+00 | HIGH |
| 8.071 | 3.999 | 2.48E+00 | HIGH |
| 21.658 | 4.870 | 2.48E+00 | HIGH |
| 7.155 | 1.143 | 2.49E+00 | HIGH |

| 10.758 | 3.314 | 2.49E+00 | HIGH |
| --- | --- | --- | --- |
| 16.011 | 8.536 | 2.50E+00 | HIGH |
| 18.985 | 5.035 | 2.50E+00 | HIGH |
| 11.589 | 6.967 | 2.50E+00 | HIGH |
| 23.576 | 2.239 | 2.50E+00 | HIGH |
| 8.989 | 6.354 | 2.50E+00 | HIGH |
| 8.719 | 4.429 | 2.50E+00 | HIGH |
| 16.058 | 4.430 | 2.51E+00 | HIGH |
| 11.574 | 5.266 | 2.51E+00 | HIGH |
| 10.344 | 5.002 | 2.51E+00 | HIGH |
| 8.249 | 3.442 | 2.52E+00 | HIGH |
| 6.603 | 2.114 | 2.52E+00 | HIGH |
| 20.157 | 7.438 | 2.53E+00 | HIGH |
| 7.830 | 3.876 | 2.53E+00 | HIGH |
| 14.440 | 5.701 | 2.53E+00 | HIGH |
| 6.688 | 2.302 | 2.53E+00 | HIGH |
| 22.278 | 6.054 | 2.54E+00 | HIGH |
| 17.853 | 5.602 | 2.55E+00 | HIGH |
| 15.138 | 4.265 | 2.55E+00 | HIGH |
| 18.358 | 8.110 | 2.55E+00 | HIGH |
| 5.047 | 5.206 | 2.55E+00 | HIGH |
| 12.448 | 2.889 | 2.56E+00 | HIGH |
| 12.107 | 4.117 | 2.56E+00 | HIGH |
| 13.503 | 4.967 | 2.56E+00 | HIGH |
| 11.695 | 1.903 | 2.56E+00 | HIGH |
| 8.761 | 3.756 | 2.57E+00 | HIGH |
| 12.431 | 0.576 | 2.57E+00 | HIGH |
| 17.942 | 3.683 | 2.59E+00 | HIGH |
| 7.443 | 2.628 | 2.60E+00 | HIGH |
| 15.479 | 2.467 | 2.61E+00 | HIGH |
| 13.849 | 2.683 | 2.64E+00 | HIGH |
| 26.095 | 4.325 | 2.65E+00 | HIGH |
| 16.826 | 3.306 | 2.65E+00 | HIGH |
| 15.969 | 2.147 | 2.65E+00 | HIGH |
| 11.287 | 4.209 | 2.67E+00 | HIGH |
| 12.741 | 2.744 | 2.67E+00 | HIGH |
| 23.363 | 7.112 | 2.67E+00 | HIGH |
| 13.822 | 4.425 | 2.67E+00 | HIGH |
| 12.174 | 4.989 | 2.67E+00 | HIGH |
| 8.805 | 0.834 | 2.68E+00 | HIGH |
| 13.135 | 1.475 | 2.69E+00 | HIGH |
| 10.127 | 4.007 | 2.69E+00 | HIGH |
| 11.631 | 4.240 | 2.71E+00 | HIGH |
| 13.210 | 4.683 | 2.71E+00 | HIGH |
| 14.077 | 5.652 | 2.71E+00 | HIGH |
| 6.815 | 3.002 | 2.71E+00 | HIGH |
| 24.671 | 6.623 | 2.71E+00 | HIGH |
| 13.785 | 6.405 | 2.73E+00 | HIGH |
| 7.567 | 3.236 | 2.74E+00 | HIGH |
| 16.760 | 4.825 | 2.75E+00 | HIGH |
| 11.109 | 4.745 | 2.75E+00 | HIGH |

| 9.920 | 5.404 | 2.76E+00 | HIGH |
| --- | --- | --- | --- |
| 10.391 | 3.797 | 2.77E+00 | HIGH |
| 9.488 | 3.601 | 2.78E+00 | HIGH |
| 13.815 | 6.097 | 2.79E+00 | HIGH |
| 5.790 | 2.813 | 2.80E+00 | HIGH |
| 13.019 | 4.502 | 2.80E+00 | HIGH |
| 19.468 | 4.846 | 2.81E+00 | HIGH |
| 7.420 | 2.945 | 2.83E+00 | HIGH |
| 12.325 | 2.374 | 2.83E+00 | HIGH |
| 13.446 | 4.444 | 2.84E+00 | HIGH |
| 10.997 | 3.669 | 2.87E+00 | HIGH |
| 16.022 | 4.503 | 2.89E+00 | HIGH |
| 7.546 | 4.322 | 2.89E+00 | HIGH |
| 8.672 | 1.963 | 2.91E+00 | HIGH |
| 8.628 | 1.651 | 2.92E+00 | HIGH |
| 10.684 | 4.168 | 2.93E+00 | HIGH |
| 22.330 | 7.917 | 2.94E+00 | HIGH |
| 19.671 | 8.582 | 2.95E+00 | HIGH |
| 12.984 | 3.108 | 2.95E+00 | HIGH |
| 8.967 | 3.734 | 2.96E+00 | HIGH |
| 9.624 | 2.993 | 2.96E+00 | HIGH |
| 17.071 | 0.741 | 2.97E+00 | HIGH |
| 16.546 | 1.038 | 2.98E+00 | HIGH |
| 7.901 | 1.669 | 3.00E+00 | HIGH |
| 17.179 | 6.356 | 3.00E+00 | HIGH |
| 11.678 | 3.641 | 3.01E+00 | HIGH |
| 37.618 | 7.446 | 3.01E+00 | HIGH |
| 12.029 | 1.896 | 3.01E+00 | HIGH |
| 8.184 | 1.744 | 3.02E+00 | HIGH |
| 14.646 | 2.792 | 3.04E+00 | HIGH |
| 18.546 | 4.693 | 3.05E+00 | HIGH |
| 9.273 | 3.364 | 3.05E+00 | HIGH |
| 14.082 | 5.427 | 3.08E+00 | HIGH |
| 13.804 | 4.185 | 3.09E+00 | HIGH |
| 20.464 | 3.999 | 3.11E+00 | HIGH |
| 14.845 | 5.974 | 3.12E+00 | HIGH |
| 17.095 | 6.036 | 3.12E+00 | HIGH |
| 20.997 | 5.990 | 3.13E+00 | HIGH |
| 12.746 | 5.606 | 3.15E+00 | HIGH |
| 14.857 | 6.213 | 3.20E+00 | HIGH |
| 9.287 | 4.266 | 3.21E+00 | HIGH |
| 15.732 | 3.361 | 3.23E+00 | HIGH |
| 30.670 | 8.326 | 3.24E+00 | HIGH |
| 14.698 | 5.239 | 3.26E+00 | HIGH |
| 12.658 | 5.271 | 3.26E+00 | HIGH |
| 9.428 | 3.681 | 3.27E+00 | HIGH |
| 14.264 | 6.418 | 3.29E+00 | HIGH |
| 23.591 | 5.470 | 3.30E+00 | HIGH |
| 5.054 | 1.792 | 3.31E+00 | HIGH |
| 25.073 | 5.376 | 3.32E+00 | HIGH |
| 19.030 | 2.478 | 3.33E+00 | HIGH |

| 11.932 | 4.562 | 3.33E+00 | HIGH |
| --- | --- | --- | --- |
| 20.095 | 2.261 | 3.36E+00 | HIGH |
| 25.061 | 7.033 | 3.37E+00 | HIGH |
| 18.053 | 1.394 | 3.39E+00 | HIGH |
| 11.542 | 4.721 | 3.40E+00 | HIGH |
| 18.582 | 3.828 | 3.43E+00 | HIGH |
| 10.099 | 3.161 | 3.44E+00 | HIGH |
| 18.063 | 0.818 | 3.44E+00 | HIGH |
| 34.073 | 4.827 | 3.45E+00 | HIGH |
| 9.177 | 2.706 | 3.48E+00 | HIGH |
| 25.444 | 4.574 | 3.49E+00 | HIGH |
| 19.383 | 5.644 | 3.49E+00 | HIGH |
| 10.299 | 1.589 | 3.49E+00 | HIGH |
| 8.634 | 2.679 | 3.55E+00 | HIGH |
| 15.494 | 1.487 | 3.58E+00 | HIGH |
| 12.856 | 3.174 | 3.59E+00 | HIGH |
| 20.545 | 3.319 | 3.59E+00 | HIGH |
| 16.456 | 3.797 | 3.59E+00 | HIGH |
| 10.554 | 1.634 | 3.60E+00 | HIGH |
| 14.799 | 5.190 | 3.61E+00 | HIGH |
| 7.786 | 0.650 | 3.61E+00 | HIGH |
| 11.441 | 2.692 | 3.62E+00 | HIGH |
| 13.354 | 1.758 | 3.66E+00 | HIGH |
| 5.937 | 2.668 | 3.72E+00 | HIGH |
| 13.407 | 7.047 | 3.79E+00 | HIGH |
| 11.537 | 3.522 | 3.84E+00 | HIGH |
| 18.010 | 4.571 | 3.89E+00 | HIGH |
| 22.985 | 6.655 | 4.06E+00 | HIGH |
| 10.624 | 2.635 | 4.10E+00 | HIGH |
| 15.003 | 5.220 | 4.14E+00 | HIGH |
| 11.133 | 4.664 | 4.15E+00 | HIGH |
| 16.908 | 4.740 | 4.16E+00 | HIGH |
| 10.010 | 1.578 | 4.21E+00 | HIGH |
| 27.094 | 5.053 | 4.30E+00 | HIGH |
| 16.431 | 5.088 | 4.60E+00 | HIGH |
| 12.046 | 3.246 | 4.65E+00 | HIGH |
| 29.660 | 4.554 | 4.68E+00 | HIGH |
| 8.171 | 3.619 | 4.99E+00 | HIGH |

36.690 0.248 6.72E+00 HIGH
