# Supplementary material for: Identification of ZDHHC1 as a Pyroptosis Inducer and Potential Target in the Establishment of Pyroptosis-Related Signature in Localized Prostate Cancer
Source: Oxid Med Cell Longev. 2022 Dec 22;2022:5925817. doi: 10.1155/2022/5925817 (PMC9800907; doi:10.1155/2022/5925817)
Supplement: Supplementary 6 — Supplementary Table 6: associations between prognostic risk signature and clinical features. [file 5925817.f6.docx]

ID bRFS time (y) Age (0:＜60;1:≥60) T N M

Cancer status (0:Discrepancy 1:Tumor free 2:Unknown

3:With tumor)

| TCGA-2A-A8VL | 1.701 | 0 | 2 | 0 | 0 | 1 |
| --- | --- | --- | --- | --- | --- | --- |
| TCGA-2A-A8VO | 3.649 | 0 | 1 | 2 | 0 | 1 |
| TCGA-2A-A8VT | 2.726 | 0 | 2 | 1 | 0 | 0 |
| TCGA-2A-A8VV | 1.838 | 0 | 2 | 0 | 0 | 1 |
| TCGA-2A-A8VX | 2.830 | 1 | 2 | 0 | 0 | 1 |
| TCGA-2A-A8W1 | 0.307 | 0 | 1 | 0 | 0 | 2 |
| TCGA-2A-A8W3 | 0.542 | 1 | 2 | 0 | 0 | 0 |
| TCGA-2A-AAYF | 2.663 | 0 | 1 | 2 | 0 | 1 |
| TCGA-2A-AAYO | 2.526 | 0 | 1 | 2 | 0 | 1 |
| TCGA-2A-AAYU | 1.685 | 0 | 1 | 0 | 0 | 1 |
| TCGA-4L-AA1F | 0.959 | 1 | 2 | 0 | 1 | 1 |
| TCGA-CH-5737 | 0.249 | 1 | 2 | 0 | 0 | 1 |
| TCGA-CH-5738 | 0.581 | 1 | 4 | 2 | 0 | 1 |
| TCGA-CH-5739 | 1.838 | 1 | 3 | 0 | 0 | 1 |
| TCGA-CH-5740 | 0.085 | 0 | 2 | 0 | 0 | 1 |
| TCGA-CH-5741 | 1.082 | 0 | 3 | 1 | 0 | 1 |
| TCGA-CH-5743 | 1.164 | 1 | 2 | 0 | 0 | 1 |
| TCGA-CH-5744 | 0.164 | 1 | 2 | 0 | 0 | 1 |
| TCGA-CH-5745 | 0.249 | 1 | 3 | 0 | 0 | 1 |
| TCGA-CH-5746 | 2.003 | 0 | 2 | 0 | 0 | 1 |
| TCGA-CH-5748 | 0.085 | 1 | 3 | 0 | 2 | 3 |
| TCGA-CH-5750 | 1.085 | 1 | 2 | 0 | 0 | 1 |
| TCGA-CH-5751 | 2.918 | 1 | 4 | 1 | 0 | 1 |
| TCGA-CH-5752 | 2.584 | 1 | 3 | 0 | 0 | 1 |
| TCGA-CH-5753 | 0.085 | 1 | 3 | 1 | 0 | 1 |
| TCGA-CH-5754 | 0.170 | 1 | 3 | 1 | 0 | 3 |
| TCGA-CH-5761 | 0.077 | 1 | 3 | 0 | 0 | 1 |
| TCGA-CH-5762 | 3.668 | 1 | 3 | 0 | 0 | 0 |
| TCGA-CH-5763 | 1.000 | 1 | 3 | 0 | 0 | 1 |
| TCGA-CH-5764 | 0.085 | 1 | 3 | 0 | 0 | 1 |
| TCGA-CH-5765 | 1.918 | 0 | 3 | 0 | 0 | 1 |
| TCGA-CH-5766 | 0.085 | 0 | 3 | 1 | 0 | 1 |
| TCGA-CH-5767 | 1.255 | 1 | 2 | 0 | 0 | 1 |
| TCGA-CH-5768 | 2.003 | 1 | 3 | 0 | 0 | 1 |
| TCGA-CH-5769 | 0.170 | 0 | 3 | 0 | 0 | 3 |
| TCGA-CH-5771 | 1.085 | 1 | 2 | 0 | 0 | 1 |
| TCGA-CH-5772 | 1.332 | 1 | 3 | 0 | 0 | 1 |
| TCGA-CH-5788 | 2.153 | 1 | 3 | 1 | 0 | 0 |
| TCGA-CH-5789 | 0.833 | 1 | 3 | 0 | 0 | 1 |
| TCGA-CH-5790 | 2.668 | 1 | 2 | 0 | 0 | 1 |
| TCGA-CH-5791 | 2.751 | 1 | 3 | 0 | 0 | 0 |
| TCGA-CH-5792 | 0.249 | 0 | 3 | 0 | 0 | 3 |
| TCGA-CH-5794 | 2.416 | 1 | 2 | 0 | 0 | 1 |
| TCGA-EJ-5494 | 1.036 | 0 | 2 | 0 | 0 | 1 |
| TCGA-EJ-5495 | 1.660 | 1 | 1 | 1 | 0 | 1 |
| TCGA-EJ-5496 | 1.630 | 0 | 1 | 0 | 0 | 1 |

| TCGA-EJ-5497 | 1.110 | 0 | 2 | 0 | 0 | 1 |
| --- | --- | --- | --- | --- | --- | --- |
| TCGA-EJ-5498 | 1.471 | 0 | 2 | 0 | 2 | 1 |
| TCGA-EJ-5499 | 1.460 | 1 | 2 | 0 | 0 | 1 |
| TCGA-EJ-5501 | 1.997 | 0 | 2 | 0 | 0 | 1 |
| TCGA-EJ-5502 | 0.671 | 0 | 2 | 0 | 0 | 1 |
| TCGA-EJ-5503 | 1.452 | 0 | 2 | 0 | 0 | 1 |
| TCGA-EJ-5504 | 0.211 | 1 | 2 | 1 | 0 | 3 |
| TCGA-EJ-5505 | 1.227 | 0 | 1 | 0 | 0 | 1 |
| TCGA-EJ-5506 | 0.975 | 1 | 3 | 1 | 0 | 1 |
| TCGA-EJ-5507 | 0.499 | 0 | 2 | 1 | 0 | 1 |
| TCGA-EJ-5508 | 2.485 | 1 | 1 | 0 | 0 | 1 |
| TCGA-EJ-5509 | 3.019 | 1 | 2 | 0 | 0 | 1 |
| TCGA-EJ-5510 | 2.022 | 0 | 1 | 0 | 0 | 1 |
| TCGA-EJ-5511 | 2.159 | 0 | 2 | 0 | 0 | 1 |
| TCGA-EJ-5512 | 2.142 | 0 | 1 | 0 | 0 | 1 |
| TCGA-EJ-5514 | 1.984 | 1 | 1 | 0 | 0 | 1 |
| TCGA-EJ-5515 | 2.907 | 1 | 1 | 0 | 0 | 1 |
| TCGA-EJ-5516 | 2.145 | 0 | 2 | 0 | 0 | 1 |
| TCGA-EJ-5517 | 2.329 | 0 | 1 | 0 | 0 | 1 |
| TCGA-EJ-5518 | 3.274 | 1 | 3 | 0 | 0 | 3 |
| TCGA-EJ-5519 | 0.230 | 1 | 2 | 1 | 0 | 1 |
| TCGA-EJ-5521 | 2.307 | 1 | 1 | 0 | 0 | 1 |
| TCGA-EJ-5522 | 3.844 | 0 | 2 | 0 | 0 | 1 |
| TCGA-EJ-5524 | 2.285 | 0 | 2 | 0 | 0 | 3 |
| TCGA-EJ-5525 | 0.926 | 1 | 3 | 0 | 0 | 3 |
| TCGA-EJ-5526 | 1.553 | 0 | 1 | 1 | 0 | 3 |
| TCGA-EJ-5527 | 1.088 | 1 | 2 | 0 | 0 | 1 |
| TCGA-EJ-5530 | 3.485 | 1 | 2 | 0 | 0 | 1 |
| TCGA-EJ-5531 | 1.984 | 1 | 2 | 0 | 0 | 1 |
| TCGA-EJ-5532 | 2.537 | 0 | 1 | 0 | 0 | 1 |
| TCGA-EJ-5542 | 1.422 | 1 | 2 | 0 | 0 | 1 |
| TCGA-EJ-7115 | 2.252 | 1 | 1 | 0 | 0 | 1 |
| TCGA-EJ-7123 | 4.085 | 0 | 1 | 0 | 0 | 1 |
| TCGA-EJ-7125 | 5.096 | 0 | 1 | 0 | 0 | 1 |
| TCGA-EJ-7218 | 6.964 | 1 | 2 | 0 | 0 | 1 |
| TCGA-EJ-7312 | 1.816 | 0 | 3 | 0 | 0 | 1 |
| TCGA-EJ-7314 | 0.808 | 1 | 2 | 0 | 0 | 1 |
| TCGA-EJ-7315 | 0.792 | 1 | 2 | 0 | 0 | 1 |
| TCGA-EJ-7317 | 0.781 | 1 | 1 | 0 | 0 | 1 |
| TCGA-EJ-7318 | 0.518 | 0 | 3 | 2 | 0 | 3 |
| TCGA-EJ-7321 | 0.638 | 0 | 1 | 0 | 0 | 1 |
| TCGA-EJ-7325 | 1.460 | 1 | 3 | 0 | 0 | 1 |
| TCGA-EJ-7327 | 0.479 | 1 | 2 | 0 | 0 | 1 |
| TCGA-EJ-7328 | 0.466 | 1 | 1 | 0 | 0 | 1 |
| TCGA-EJ-7330 | 0.523 | 1 | 1 | 0 | 0 | 1 |
| TCGA-EJ-7331 | 0.518 | 1 | 1 | 0 | 0 | 1 |
| TCGA-EJ-7781 | 0.463 | 1 | 1 | 0 | 0 | 1 |
| TCGA-EJ-7782 | 0.959 | 1 | 2 | 0 | 0 | 1 |
| TCGA-EJ-7783 | 0.222 | 1 | 2 | 0 | 0 | 1 |
| TCGA-EJ-7784 | 0.458 | 1 | 2 | 0 | 0 | 1 |
| TCGA-EJ-7785 | 0.710 | 0 | 2 | 0 | 0 | 1 |

| TCGA-EJ-7786 | 0.216 | 1 | 1 | 0 | 0 | 1 |
| --- | --- | --- | --- | --- | --- | --- |
| TCGA-EJ-7788 | 0.142 | 0 | 3 | 0 | 0 | 1 |
| TCGA-EJ-7789 | 0.192 | 1 | 1 | 0 | 0 | 1 |
| TCGA-EJ-7791 | 0.595 | 1 | 1 | 0 | 0 | 1 |
| TCGA-EJ-7792 | 1.441 | 0 | 1 | 0 | 0 | 1 |
| TCGA-EJ-7793 | 0.312 | 0 | 2 | 2 | 0 | 1 |
| TCGA-EJ-7794 | 0.781 | 1 | 2 | 0 | 0 | 1 |
| TCGA-EJ-7797 | 0.512 | 0 | 1 | 0 | 0 | 1 |
| TCGA-EJ-8468 | 5.373 | 1 | 3 | 0 | 2 | 1 |
| TCGA-EJ-8469 | 5.274 | 0 | 3 | 0 | 0 | 3 |
| TCGA-EJ-8470 | 2.249 | 0 | 2 | 0 | 0 | 1 |
| TCGA-EJ-8472 | 0.537 | 1 | 3 | 0 | 0 | 1 |
| TCGA-EJ-8474 | 0.860 | 1 | 3 | 0 | 0 | 1 |
| TCGA-EJ-A46B | 0.444 | 1 | 1 | 0 | 0 | 1 |
| TCGA-EJ-A46D | 0.353 | 0 | 2 | 0 | 0 | 1 |
| TCGA-EJ-A46E | 1.436 | 0 | 1 | 0 | 0 | 1 |
| TCGA-EJ-A46F | 0.589 | 0 | 2 | 1 | 0 | 1 |
| TCGA-EJ-A46G | 0.255 | 1 | 3 | 0 | 0 | 1 |
| TCGA-EJ-A46H | 0.627 | 1 | 1 | 0 | 0 | 1 |
| TCGA-EJ-A46I | 1.830 | 0 | 1 | 0 | 0 | 1 |
| TCGA-EJ-A65B | 1.468 | 0 | 3 | 0 | 0 | 1 |
| TCGA-EJ-A65D | 1.077 | 1 | 3 | 0 | 0 | 1 |
| TCGA-EJ-A65E | 1.142 | 1 | 3 | 0 | 0 | 1 |
| TCGA-EJ-A65F | 0.205 | 0 | 3 | 0 | 0 | 0 |
| TCGA-EJ-A65G | 0.762 | 0 | 2 | 0 | 0 | 1 |
| TCGA-EJ-A65J | 0.249 | 1 | 3 | 0 | 0 | 1 |
| TCGA-EJ-A65M | 0.630 | 1 | 2 | 0 | 0 | 1 |
| TCGA-EJ-A6RA | 0.967 | 1 | 3 | 1 | 0 | 3 |
| TCGA-EJ-A6RC | 1.899 | 1 | 1 | 0 | 0 | 1 |
| TCGA-EJ-A7NF | 0.592 | 0 | 2 | 0 | 0 | 1 |
| TCGA-EJ-A7NG | 0.723 | 1 | 3 | 0 | 0 | 1 |
| TCGA-EJ-A7NH | 0.471 | 0 | 2 | 0 | 0 | 1 |
| TCGA-EJ-A7NJ | 0.540 | 0 | 2 | 0 | 0 | 1 |
| TCGA-EJ-A7NK | 0.808 | 0 | 3 | 0 | 0 | 1 |
| TCGA-EJ-A7NM | 0.351 | 1 | 3 | 1 | 0 | 1 |
| TCGA-EJ-A7NN | 0.540 | 1 | 3 | 1 | 0 | 1 |
| TCGA-EJ-A8FN | 0.556 | 0 | 2 | 0 | 0 | 1 |
| TCGA-EJ-A8FO | 0.427 | 0 | 3 | 0 | 0 | 1 |
| TCGA-EJ-A8FP | 0.321 | 0 | 2 | 0 | 0 | 3 |
| TCGA-EJ-A8FS | 0.592 | 1 | 3 | 0 | 0 | 3 |
| TCGA-EJ-A8FU | 0.225 | 1 | 3 | 0 | 0 | 1 |
| TCGA-EJ-AB20 | 0.359 | 1 | 2 | 0 | 0 | 1 |
| TCGA-EJ-AB27 | 0.397 | 0 | 1 | 0 | 0 | 1 |
| TCGA-FC-7708 | 0.088 | 0 | 1 | 0 | 0 | 1 |
| TCGA-FC-7961 | 0.192 | 1 | 1 | 0 | 0 | 1 |
| TCGA-FC-A4JI | 0.079 | 1 | 4 | 2 | 0 | 1 |
| TCGA-FC-A5OB | 0.748 | 0 | 3 | 0 | 0 | 3 |
| TCGA-FC-A66V | 0.334 | 1 | 3 | 0 | 0 | 1 |
| TCGA-FC-A6HD | 0.148 | 1 | 3 | 0 | 0 | 2 |
| TCGA-FC-A8O0 | 0.148 | 1 | 2 | 0 | 0 | 1 |
| TCGA-G9-6329 | 2.529 | 1 | 1 | 2 | 0 | 1 |

| TCGA-G9-6332 | 6.378 | 0 | 2 | 0 | 0 | 1 |
| --- | --- | --- | --- | --- | --- | --- |
| TCGA-G9-6333 | 5.274 | 1 | 1 | 0 | 0 | 1 |
| TCGA-G9-6336 | 4.573 | 0 | 2 | 0 | 0 | 1 |
| TCGA-G9-6338 | 4.518 | 1 | 1 | 0 | 0 | 3 |
| TCGA-G9-6339 | 4.477 | 0 | 3 | 0 | 0 | 1 |
| TCGA-G9-6342 | 3.666 | 1 | 2 | 2 | 0 | 1 |
| TCGA-G9-6343 | 1.518 | 1 | 2 | 2 | 0 | 1 |
| TCGA-G9-6347 | 4.707 | 0 | 1 | 2 | 0 | 1 |
| TCGA-G9-6348 | 3.134 | 1 | 3 | 0 | 0 | 1 |
| TCGA-G9-6351 | 3.329 | 0 | 1 | 2 | 0 | 1 |
| TCGA-G9-6353 | 2.323 | 0 | 1 | 2 | 0 | 1 |
| TCGA-G9-6354 | 4.112 | 0 | 1 | 2 | 0 | 1 |
| TCGA-G9-6356 | 2.740 | 1 | 1 | 0 | 0 | 1 |
| TCGA-G9-6361 | 2.762 | 1 | 2 | 0 | 0 | 1 |
| TCGA-G9-6362 | 2.252 | 0 | 1 | 0 | 0 | 1 |
| TCGA-G9-6363 | 2.721 | 1 | 2 | 0 | 0 | 1 |
| TCGA-G9-6364 | 2.304 | 1 | 3 | 0 | 0 | 1 |
| TCGA-G9-6365 | 2.203 | 1 | 2 | 0 | 2 | 1 |
| TCGA-G9-6366 | 0.277 | 1 | 1 | 2 | 2 | 0 |
| TCGA-G9-6367 | 2.397 | 1 | 2 | 0 | 0 | 3 |
| TCGA-G9-6369 | 2.332 | 0 | 1 | 0 | 0 | 1 |
| TCGA-G9-6370 | 2.208 | 0 | 1 | 0 | 0 | 1 |
| TCGA-G9-6371 | 2.318 | 0 | 1 | 2 | 0 | 1 |
| TCGA-G9-6373 | 1.384 | 1 | 2 | 2 | 0 | 1 |
| TCGA-G9-6377 | 1.419 | 1 | 2 | 0 | 0 | 1 |
| TCGA-G9-6378 | 1.819 | 0 | 1 | 2 | 0 | 1 |
| TCGA-G9-6379 | 3.816 | 1 | 1 | 0 | 0 | 1 |
| TCGA-G9-6384 | 1.068 | 0 | 1 | 0 | 0 | 1 |
| TCGA-G9-6385 | 0.975 | 1 | 1 | 2 | 0 | 1 |
| TCGA-G9-6494 | 3.951 | 1 | 1 | 0 | 0 | 3 |
| TCGA-G9-6496 | 2.778 | 1 | 1 | 0 | 0 | 1 |
| TCGA-G9-6498 | 3.677 | 0 | 1 | 2 | 0 | 1 |
| TCGA-G9-6499 | 3.038 | 1 | 3 | 0 | 0 | 1 |
| TCGA-G9-7509 | 3.189 | 0 | 1 | 2 | 0 | 1 |
| TCGA-G9-7510 | 1.847 | 1 | 1 | 0 | 0 | 1 |
| TCGA-G9-7519 | 1.252 | 0 | 1 | 2 | 0 | 1 |
| TCGA-G9-7521 | 1.334 | 0 | 3 | 1 | 0 | 1 |
| TCGA-G9-7522 | 1.238 | 0 | 1 | 0 | 0 | 1 |
| TCGA-G9-7523 | 0.888 | 0 | 1 | 0 | 0 | 1 |
| TCGA-G9-7525 | 1.403 | 1 | 1 | 0 | 0 | 1 |
| TCGA-G9-A9S0 | 1.175 | 0 | 2 | 1 | 0 | 3 |
| TCGA-G9-A9S4 | 2.074 | 1 | 2 | 1 | 0 | 1 |
| TCGA-G9-A9S7 | 1.762 | 0 | 2 | 0 | 0 | 3 |
| TCGA-H9-7775 | 0.121 | 1 | 2 | 2 | 2 | 0 |
| TCGA-H9-A6BX | 1.562 | 1 | 2 | 2 | 2 | 1 |
| TCGA-H9-A6BY | 0.307 | 1 | 1 | 0 | 2 | 2 |
| TCGA-HC-7075 | 0.173 | 1 | 1 | 2 | 0 | 1 |
| TCGA-HC-7077 | 0.211 | 1 | 1 | 2 | 0 | 1 |
| TCGA-HC-7078 | 0.444 | 1 | 1 | 0 | 0 | 1 |
| TCGA-HC-7079 | 0.345 | 0 | 3 | 0 | 0 | 3 |
| TCGA-HC-7080 | 0.285 | 1 | 2 | 0 | 0 | 1 |

| TCGA-HC-7081 | 0.197 | 1 | 3 | 2 | 2 | 3 |
| --- | --- | --- | --- | --- | --- | --- |
| TCGA-HC-7209 | 0.112 | 1 | 2 | 0 | 2 | 1 |
| TCGA-HC-7210 | 0.408 | 1 | 2 | 0 | 2 | 1 |
| TCGA-HC-7211 | 0.066 | 1 | 2 | 0 | 0 | 1 |
| TCGA-HC-7212 | 0.112 | 0 | 2 | 0 | 0 | 0 |
| TCGA-HC-7213 | 0.466 | 0 | 3 | 0 | 0 | 1 |
| TCGA-HC-7230 | 0.140 | 0 | 2 | 0 | 2 | 1 |
| TCGA-HC-7231 | 0.197 | 1 | 1 | 2 | 0 | 1 |
| TCGA-HC-7232 | 0.244 | 1 | 3 | 2 | 2 | 1 |
| TCGA-HC-7233 | 0.142 | 1 | 2 | 0 | 2 | 3 |
| TCGA-HC-7736 | 0.060 | 0 | 2 | 0 | 0 | 1 |
| TCGA-HC-7737 | 0.175 | 0 | 1 | 1 | 0 | 1 |
| TCGA-HC-7738 | 0.222 | 0 | 2 | 2 | 0 | 1 |
| TCGA-HC-7740 | 0.145 | 0 | 2 | 0 | 0 | 1 |
| TCGA-HC-7742 | 0.310 | 0 | 3 | 0 | 0 | 3 |
| TCGA-HC-7744 | 0.140 | 0 | 3 | 0 | 0 | 3 |
| TCGA-HC-7745 | 0.173 | 1 | 3 | 0 | 2 | 1 |
| TCGA-HC-7747 | 0.156 | 0 | 2 | 0 | 0 | 1 |
| TCGA-HC-7748 | 0.170 | 1 | 2 | 0 | 2 | 1 |
| TCGA-HC-7749 | 0.164 | 1 | 3 | 0 | 0 | 1 |
| TCGA-HC-7750 | 0.093 | 0 | 2 | 2 | 0 | 1 |
| TCGA-HC-7752 | 0.268 | 1 | 2 | 0 | 0 | 1 |
| TCGA-HC-7817 | 0.088 | 0 | 3 | 2 | 0 | 1 |
| TCGA-HC-7818 | 0.123 | 1 | 2 | 0 | 0 | 1 |
| TCGA-HC-7819 | 0.003 | 0 | 2 | 0 | 0 | 3 |
| TCGA-HC-7820 | 0.279 | 1 | 2 | 2 | 2 | 1 |
| TCGA-HC-7821 | 0.153 | 0 | 3 | 1 | 0 | 3 |
| TCGA-HC-8213 | 0.003 | 0 | 2 | 2 | 2 | 1 |
| TCGA-HC-8216 | 0.115 | 0 | 2 | 0 | 0 | 1 |
| TCGA-HC-8256 | 0.260 | 0 | 2 | 2 | 0 | 1 |
| TCGA-HC-8257 | 0.079 | 1 | 3 | 1 | 2 | 3 |
| TCGA-HC-8258 | 0.181 | 0 | 2 | 0 | 2 | 1 |
| TCGA-HC-8259 | 0.337 | 0 | 1 | 2 | 0 | 1 |
| TCGA-HC-8260 | 0.290 | 0 | 2 | 0 | 0 | 3 |
| TCGA-HC-8261 | 0.170 | 0 | 2 | 2 | 2 | 1 |
| TCGA-HC-8262 | 0.208 | 0 | 2 | 0 | 2 | 1 |
| TCGA-HC-8264 | 0.132 | 1 | 3 | 1 | 2 | 3 |
| TCGA-HC-8265 | 0.192 | 1 | 3 | 0 | 2 | 3 |
| TCGA-HC-8266 | 0.096 | 0 | 3 | 0 | 0 | 3 |
| TCGA-HC-A48F | 0.126 | 0 | 1 | 1 | 0 | 3 |
| TCGA-HC-A4ZV | 0.063 | 0 | 2 | 0 | 2 | 3 |
| TCGA-HC-A631 | 0.148 | 1 | 2 | 0 | 0 | 3 |
| TCGA-HC-A632 | 0.167 | 1 | 1 | 0 | 0 | 1 |
| TCGA-HC-A6AL | 0.184 | 1 | 1 | 0 | 0 | 1 |
| TCGA-HC-A6AN | 0.134 | 1 | 1 | 0 | 0 | 3 |
| TCGA-HC-A6AO | 0.458 | 0 | 1 | 2 | 0 | 1 |
| TCGA-HC-A6AP | 0.195 | 1 | 1 | 2 | 0 | 2 |
| TCGA-HC-A6AQ | 0.290 | 1 | 1 | 2 | 0 | 1 |
| TCGA-HC-A6AS | 0.121 | 1 | 1 | 0 | 0 | 1 |
| TCGA-HC-A6HX | 0.104 | 0 | 1 | 0 | 0 | 1 |
| TCGA-HC-A6HY | 0.367 | 0 | 1 | 2 | 0 | 1 |

| TCGA-HC-A76W | 0.584 | 1 | 2 | 0 | 0 | 3 |
| --- | --- | --- | --- | --- | --- | --- |
| TCGA-HC-A76X | 0.411 | 1 | 1 | 0 | 0 | 1 |
| TCGA-HC-A8CY | 0.795 | 1 | 1 | 1 | 0 | 3 |
| TCGA-HC-A8D0 | 0.301 | 1 | 1 | 0 | 0 | 1 |
| TCGA-HC-A8D1 | 0.460 | 1 | 2 | 0 | 0 | 1 |
| TCGA-HC-A9TE | 0.367 | 1 | 2 | 0 | 0 | 3 |
| TCGA-HC-A9TH | 0.808 | 0 | 1 | 0 | 0 | 3 |
| TCGA-HI-7168 | 0.844 | 1 | 1 | 0 | 0 | 0 |
| TCGA-HI-7169 | 5.340 | 0 | 2 | 0 | 0 | 1 |
| TCGA-HI-7170 | 4.877 | 0 | 1 | 0 | 0 | 1 |
| TCGA-HI-7171 | 0.597 | 0 | 3 | 0 | 0 | 3 |
| TCGA-J4-8198 | 0.268 | 0 | 1 | 2 | 0 | 2 |
| TCGA-J4-8200 | 0.537 | 0 | 2 | 0 | 0 | 1 |
| TCGA-J4-A67K | 1.814 | 1 | 2 | 2 | 0 | 1 |
| TCGA-J4-A67L | 1.419 | 0 | 2 | 0 | 0 | 1 |
| TCGA-J4-A67M | 1.337 | 0 | 2 | 0 | 0 | 1 |
| TCGA-J4-A67N | 1.211 | 1 | 2 | 0 | 0 | 3 |
| TCGA-J4-A67O | 1.345 | 0 | 2 | 0 | 0 | 1 |
| TCGA-J4-A67Q | 1.710 | 1 | 2 | 2 | 0 | 1 |
| TCGA-J4-A67R | 1.244 | 1 | 1 | 0 | 0 | 1 |
| TCGA-J4-A67S | 1.433 | 1 | 2 | 0 | 0 | 3 |
| TCGA-J4-A67T | 0.501 | 1 | 1 | 2 | 0 | 1 |
| TCGA-J4-A6G1 | 1.110 | 1 | 2 | 0 | 0 | 2 |
| TCGA-J4-A6G3 | 1.447 | 0 | 2 | 0 | 0 | 1 |
| TCGA-J4-A6M7 | 0.764 | 0 | 2 | 2 | 0 | 1 |
| TCGA-J4-A83I | 0.764 | 1 | 1 | 0 | 0 | 1 |
| TCGA-J4-A83J | 0.849 | 1 | 1 | 0 | 0 | 2 |
| TCGA-J4-A83K | 0.564 | 0 | 1 | 2 | 0 | 1 |
| TCGA-J4-A83L | 0.866 | 1 | 1 | 0 | 0 | 1 |
| TCGA-J4-A83M | 1.197 | 1 | 2 | 0 | 0 | 2 |
| TCGA-J4-A83N | 1.690 | 0 | 1 | 2 | 0 | 1 |
| TCGA-J4-AATV | 0.981 | 1 | 2 | 0 | 0 | 2 |
| TCGA-J4-AATZ | 0.216 | 1 | 3 | 0 | 0 | 3 |
| TCGA-J4-AAU2 | 0.833 | 0 | 2 | 0 | 0 | 1 |
| TCGA-J9-A52B | 0.233 | 1 | 3 | 0 | 2 | 2 |
| TCGA-J9-A52C | 0.488 | 0 | 1 | 2 | 2 | 2 |
| TCGA-J9-A52D | 0.581 | 1 | 2 | 2 | 0 | 1 |
| TCGA-J9-A52E | 0.244 | 1 | 2 | 0 | 2 | 1 |
| TCGA-J9-A8CK | 0.879 | 1 | 3 | 2 | 0 | 1 |
| TCGA-J9-A8CL | 0.362 | 1 | 3 | 2 | 0 | 3 |
| TCGA-J9-A8CM | 0.942 | 1 | 2 | 1 | 0 | 3 |
| TCGA-J9-A8CN | 3.389 | 0 | 1 | 2 | 0 | 1 |
| TCGA-J9-A8CP | 1.058 | 1 | 2 | 2 | 0 | 1 |
| TCGA-KC-A4BL | 0.529 | 1 | 1 | 0 | 0 | 3 |
| TCGA-KC-A4BN | 4.973 | 0 | 1 | 0 | 0 | 1 |
| TCGA-KC-A4BR | 2.800 | 1 | 2 | 1 | 0 | 3 |
| TCGA-KC-A4BV | 3.638 | 1 | 2 | 0 | 0 | 3 |
| TCGA-KC-A7F3 | 1.822 | 1 | 1 | 0 | 0 | 1 |
| TCGA-KC-A7F5 | 0.249 | 0 | 2 | 0 | 0 | 1 |
| TCGA-KC-A7F6 | 0.721 | 1 | 1 | 0 | 0 | 1 |
| TCGA-KC-A7FA | 1.638 | 1 | 2 | 0 | 0 | 1 |

| TCGA-KC-A7FD | 0.740 | 1 | 1 | 0 | 0 | 1 |
| --- | --- | --- | --- | --- | --- | --- |
| TCGA-KC-A7FE | 0.964 | 1 | 1 | 0 | 0 | 1 |
| TCGA-KK-A59V | 7.833 | 1 | 3 | 0 | 0 | 1 |
| TCGA-KK-A59X | 4.690 | 0 | 3 | 1 | 0 | 1 |
| TCGA-KK-A59Y | 3.723 | 0 | 3 | 1 | 0 | 1 |
| TCGA-KK-A59Z | 5.148 | 1 | 2 | 0 | 0 | 1 |
| TCGA-KK-A5A1 | 6.477 | 1 | 3 | 0 | 0 | 2 |
| TCGA-KK-A6DY | 9.655 | 0 | 1 | 0 | 0 | 3 |
| TCGA-KK-A6E0 | 2.578 | 0 | 2 | 0 | 0 | 3 |
| TCGA-KK-A6E1 | 4.545 | 0 | 2 | 1 | 0 | 1 |
| TCGA-KK-A6E2 | 12.614 | 0 | 2 | 0 | 0 | 1 |
| TCGA-KK-A6E3 | 5.633 | 0 | 1 | 0 | 0 | 1 |
| TCGA-KK-A6E4 | 7.542 | 1 | 1 | 0 | 0 | 1 |
| TCGA-KK-A6E5 | 4.104 | 1 | 1 | 0 | 0 | 1 |
| TCGA-KK-A6E6 | 9.444 | 1 | 2 | 0 | 0 | 1 |
| TCGA-KK-A6E7 | 5.589 | 0 | 1 | 1 | 0 | 3 |
| TCGA-KK-A6E8 | 4.849 | 1 | 1 | 0 | 0 | 1 |
| TCGA-KK-A7AP | 0.537 | 0 | 2 | 1 | 0 | 2 |
| TCGA-KK-A7AQ | 3.334 | 1 | 2 | 0 | 0 | 1 |
| TCGA-KK-A7AU | 0.567 | 1 | 2 | 1 | 0 | 1 |
| TCGA-KK-A7AV | 2.252 | 0 | 1 | 0 | 0 | 1 |
| TCGA-KK-A7AW | 0.559 | 0 | 2 | 1 | 0 | 1 |
| TCGA-KK-A7AY | 3.079 | 1 | 2 | 0 | 0 | 1 |
| TCGA-KK-A7AZ | 2.301 | 0 | 2 | 0 | 2 | 2 |
| TCGA-KK-A7B0 | 1.660 | 1 | 2 | 0 | 0 | 2 |
| TCGA-KK-A7B1 | 2.912 | 1 | 1 | 0 | 0 | 2 |
| TCGA-KK-A7B2 | 1.896 | 1 | 1 | 1 | 0 | 3 |
| TCGA-KK-A7B3 | 1.745 | 1 | 3 | 0 | 0 | 1 |
| TCGA-KK-A7B4 | 1.745 | 1 | 3 | 1 | 0 | 3 |
| TCGA-KK-A8I4 | 3.164 | 1 | 2 | 1 | 0 | 3 |
| TCGA-KK-A8I5 | 6.995 | 0 | 2 | 0 | 0 | 1 |
| TCGA-KK-A8I6 | 1.830 | 0 | 2 | 0 | 0 | 1 |
| TCGA-KK-A8I7 | 2.981 | 0 | 2 | 0 | 0 | 1 |
| TCGA-KK-A8I8 | 2.121 | 1 | 2 | 0 | 0 | 1 |
| TCGA-KK-A8I9 | 1.975 | 1 | 2 | 0 | 0 | 1 |
| TCGA-KK-A8IA | 3.942 | 1 | 3 | 1 | 0 | 2 |
| TCGA-KK-A8IB | 0.227 | 1 | 2 | 0 | 0 | 2 |
| TCGA-KK-A8IC | 2.904 | 0 | 1 | 0 | 0 | 3 |
| TCGA-KK-A8ID | 5.405 | 1 | 2 | 1 | 0 | 1 |
| TCGA-KK-A8IF | 1.775 | 0 | 2 | 0 | 0 | 3 |
| TCGA-KK-A8IG | 6.058 | 0 | 2 | 0 | 0 | 1 |
| TCGA-KK-A8IH | 4.479 | 0 | 2 | 0 | 0 | 1 |
| TCGA-KK-A8II | 1.715 | 1 | 2 | 0 | 0 | 3 |
| TCGA-KK-A8IJ | 4.271 | 0 | 3 | 1 | 0 | 2 |
| TCGA-KK-A8IK | 4.282 | 0 | 1 | 0 | 0 | 1 |
| TCGA-KK-A8IL | 1.721 | 1 | 1 | 1 | 0 | 2 |
| TCGA-KK-A8IM | 3.940 | 0 | 2 | 0 | 0 | 1 |
| TCGA-M7-A71Y | 0.433 | 0 | 1 | 2 | 2 | 1 |
| TCGA-M7-A71Z | 0.573 | 1 | 2 | 0 | 2 | 1 |
| TCGA-M7-A720 | 0.827 | 0 | 1 | 2 | 2 | 1 |
| TCGA-M7-A721 | 0.559 | 1 | 1 | 2 | 0 | 1 |

| TCGA-M7-A722 | 1.532 | 1 | 2 | 0 | 0 | 3 |
| --- | --- | --- | --- | --- | --- | --- |
| TCGA-M7-A723 | 0.170 | 0 | 1 | 1 | 0 | 1 |
| TCGA-M7-A724 | 1.323 | 1 | 1 | 0 | 0 | 3 |
| TCGA-M7-A725 | 0.263 | 0 | 3 | 0 | 0 | 1 |
| TCGA-MG-AAMC | 0.474 | 0 | 1 | 0 | 0 | 1 |
| TCGA-QU-A6IL | 0.266 | 1 | 2 | 0 | 0 | 1 |
| TCGA-QU-A6IM | 3.416 | 0 | 2 | 0 | 0 | 0 |
| TCGA-QU-A6IN | 9.553 | 1 | 1 | 0 | 0 | 1 |
| TCGA-QU-A6IO | 3.934 | 0 | 1 | 0 | 0 | 1 |
| TCGA-QU-A6IP | 7.178 | 1 | 2 | 0 | 0 | 0 |
| TCGA-SU-A7E7 | 0.307 | 1 | 2 | 0 | 0 | 1 |
| TCGA-TK-A8OK | 0.074 | 1 | 4 | 2 | 0 | 0 |
| TCGA-TP-A8TT | 0.992 | 1 | 1 | 0 | 0 | 2 |
| TCGA-TP-A8TV | 0.682 | 1 | 1 | 0 | 0 | 1 |
| TCGA-V1-A8MF | 3.436 | 1 | 2 | 0 | 0 | 2 |
| TCGA-V1-A8MG | 2.348 | 0 | 2 | 2 | 0 | 2 |
| TCGA-V1-A8MK | 1.392 | 0 | 2 | 0 | 0 | 2 |
| TCGA-V1-A8ML | 1.227 | 1 | 2 | 0 | 0 | 2 |
| TCGA-V1-A8MM | 2.712 | 1 | 2 | 0 | 0 | 2 |
| TCGA-V1-A8MU | 3.833 | 0 | 2 | 1 | 0 | 2 |
| TCGA-V1-A8WL | 3.767 | 1 | 1 | 0 | 0 | 2 |
| TCGA-V1-A8WN | 1.304 | 0 | 2 | 2 | 0 | 2 |
| TCGA-V1-A8WS | 1.299 | 0 | 1 | 2 | 0 | 2 |
| TCGA-V1-A8WV | 1.879 | 0 | 2 | 1 | 0 | 2 |
| TCGA-V1-A8WW | 0.288 | 0 | 2 | 1 | 0 | 2 |
| TCGA-V1-A8X3 | 0.553 | 0 | 1 | 0 | 0 | 2 |
| TCGA-V1-A9O5 | 0.340 | 1 | 2 | 1 | 0 | 2 |
| TCGA-V1-A9O7 | 2.523 | 1 | 2 | 0 | 0 | 2 |
| TCGA-V1-A9O9 | 0.559 | 0 | 3 | 0 | 0 | 2 |
| TCGA-V1-A9OA | 1.742 | 1 | 2 | 1 | 0 | 2 |
| TCGA-V1-A9OF | 3.244 | 0 | 1 | 2 | 0 | 2 |
| TCGA-V1-A9OH | 3.433 | 1 | 1 | 2 | 0 | 2 |
| TCGA-V1-A9OL | 0.359 | 1 | 2 | 0 | 0 | 2 |
| TCGA-V1-A9OQ | 1.016 | 1 | 1 | 2 | 0 | 2 |
| TCGA-V1-A9OT | 1.304 | 1 | 1 | 2 | 0 | 2 |
| TCGA-V1-A9OX | 0.701 | 0 | 2 | 0 | 0 | 2 |
| TCGA-V1-A9OY | 0.466 | 0 | 2 | 1 | 0 | 2 |
| TCGA-V1-A9Z7 | 0.337 | 0 | 2 | 0 | 0 | 2 |
| TCGA-V1-A9Z8 | 0.512 | 0 | 3 | 0 | 0 | 2 |
| TCGA-V1-A9Z9 | 0.808 | 1 | 1 | 0 | 0 | 2 |
| TCGA-V1-A9ZG | 3.060 | 1 | 1 | 0 | 0 | 2 |
| TCGA-V1-A9ZI | 0.236 | 1 | 3 | 0 | 0 | 2 |
| TCGA-V1-A9ZK | 2.874 | 1 | 2 | 0 | 0 | 2 |
| TCGA-V1-A9ZR | 0.485 | 1 | 2 | 0 | 0 | 2 |
| TCGA-VN-A88I | 0.737 | 0 | 1 | 0 | 0 | 3 |
| TCGA-VN-A88K | 2.126 | 0 | 1 | 1 | 0 | 2 |
| TCGA-VN-A88L | 1.079 | 0 | 1 | 0 | 0 | 1 |
| TCGA-VN-A88M | 0.501 | 0 | 1 | 0 | 0 | 1 |
| TCGA-VN-A88N | 0.482 | 1 | 1 | 0 | 0 | 1 |
| TCGA-VN-A88O | 1.318 | 0 | 1 | 0 | 0 | 1 |
| TCGA-VN-A88P | 2.512 | 1 | 1 | 0 | 0 | 1 |

| TCGA-VN-A88Q | 2.742 | 1 | 2 | 0 | 0 | 1 |
| --- | --- | --- | --- | --- | --- | --- |
| TCGA-VN-A88R | 1.258 | 0 | 1 | 0 | 0 | 2 |
| TCGA-VN-A943 | 0.362 | 1 | 1 | 0 | 0 | 1 |
| TCGA-VP-A872 | 8.912 | 1 | 2 | 0 | 0 | 1 |
| TCGA-VP-A875 | 2.205 | 1 | 2 | 0 | 0 | 2 |
| TCGA-VP-A876 | 7.058 | 0 | 1 | 0 | 2 | 1 |
| TCGA-VP-A878 | 0.268 | 0 | 1 | 1 | 2 | 0 |
| TCGA-VP-A879 | 1.995 | 1 | 1 | 0 | 0 | 2 |
| TCGA-VP-A87B | 6.326 | 1 | 1 | 0 | 0 | 1 |
| TCGA-VP-A87C | 4.630 | 1 | 1 | 0 | 0 | 1 |
| TCGA-VP-A87D | 3.271 | 0 | 1 | 1 | 0 | 1 |
| TCGA-VP-A87E | 5.581 | 0 | 1 | 0 | 0 | 1 |
| TCGA-VP-A87H | 1.816 | 1 | 2 | 0 | 0 | 3 |
| TCGA-VP-A87J | 3.551 | 0 | 2 | 1 | 0 | 3 |
| TCGA-VP-A87K | 1.296 | 1 | 1 | 0 | 0 | 3 |
| TCGA-VP-AA1N | 1.485 | 1 | 1 | 0 | 0 | 1 |
| TCGA-WW-A8ZI | 0.482 | 1 | 2 | 0 | 0 | 1 |
| TCGA-X4-A8KQ | 3.099 | 1 | 1 | 0 | 0 | 1 |
| TCGA-X4-A8KS | 2.318 | 1 | 2 | 2 | 0 | 1 |
| TCGA-XA-A8JR | 0.326 | 1 | 2 | 0 | 0 | 1 |
| TCGA-XJ-A83F | 2.088 | 1 | 3 | 0 | 2 | 1 |
| TCGA-XJ-A83G | 3.170 | 0 | 1 | 0 | 2 | 1 |
| TCGA-XJ-A83H | 3.507 | 0 | 2 | 2 | 0 | 2 |
| TCGA-XJ-A9DI | 4.003 | 1 | 2 | 0 | 0 | 1 |
| TCGA-XJ-A9DK | 0.753 | 1 | 1 | 2 | 0 | 2 |
| TCGA-XJ-A9DQ | 0.249 | 0 | 1 | 2 | 0 | 2 |
| TCGA-XJ-A9DX | 2.164 | 0 | 1 | 1 | 1 | 1 |
| TCGA-XK-AAIR | 2.663 | 1 | 1 | 0 | 0 | 1 |
| TCGA-XK-AAIV | 2.625 | 1 | 1 | 1 | 0 | 3 |
| TCGA-XK-AAIW | 2.663 | 1 | 2 | 0 | 0 | 3 |
| TCGA-XK-AAJ3 | 2.378 | 0 | 1 | 0 | 0 | 1 |
| TCGA-XK-AAJA | 2.548 | 1 | 2 | 0 | 0 | 0 |
| TCGA-XK-AAJP | 2.436 | 1 | 1 | 0 | 0 | 1 |
| TCGA-XK-AAJR | 0.359 | 1 | 1 | 0 | 0 | 1 |
| TCGA-XK-AAJT | 3.085 | 1 | 1 | 0 | 0 | 3 |
| TCGA-XK-AAJU | 3.304 | 1 | 1 | 0 | 0 | 1 |
| TCGA-XK-AAK1 | 1.682 | 1 | 1 | 0 | 0 | 1 |
| TCGA-XQ-A8TA | 0.400 | 0 | 3 | 2 | 1 | 3 |
| TCGA-XQ-A8TB | 2.110 | 1 | 2 | 0 | 0 | 1 |
| TCGA-Y6-A8TL | 2.123 | 1 | 2 | 0 | 2 | 1 |
| TCGA-Y6-A9XI | 1.441 | 1 | 4 | 0 | 2 | 1 |
| TCGA-YJ-A8SW | 0.405 | 1 | 1 | 0 | 2 | 0 |
| TCGA-YL-A8HJ | 4.414 | 0 | 3 | 2 | 0 | 1 |
| TCGA-YL-A8HK | 3.770 | 0 | 3 | 0 | 0 | 3 |
| TCGA-YL-A8HL | 3.214 | 0 | 3 | 1 | 0 | 2 |
| TCGA-YL-A8HM | 3.079 | 1 | 3 | 0 | 0 | 1 |
| TCGA-YL-A8HO | 2.926 | 1 | 3 | 0 | 0 | 2 |
| TCGA-YL-A8S8 | 1.860 | 1 | 3 | 0 | 0 | 3 |
| TCGA-YL-A8S9 | 5.047 | 1 | 3 | 0 | 0 | 2 |
| TCGA-YL-A8SA | 1.403 | 1 | 2 | 0 | 0 | 2 |
| TCGA-YL-A8SB | 3.792 | 1 | 3 | 0 | 0 | 2 |

| TCGA-YL-A8SC | 3.455 | 1 | 3 | 0 | 0 | 3 |
| --- | --- | --- | --- | --- | --- | --- |
| TCGA-YL-A8SH | 2.397 | 1 | 3 | 0 | 0 | 1 |
| TCGA-YL-A8SI | 3.899 | 1 | 3 | 0 | 0 | 3 |
| TCGA-YL-A8SJ | 2.060 | 1 | 3 | 0 | 0 | 3 |
| TCGA-YL-A8SK | 3.164 | 1 | 3 | 1 | 0 | 2 |
| TCGA-YL-A8SL | 2.186 | 1 | 3 | 1 | 0 | 1 |
| TCGA-YL-A8SO | 8.896 | 1 | 1 | 2 | 0 | 2 |
| TCGA-YL-A8SP | 5.945 | 0 | 2 | 2 | 0 | 3 |
| TCGA-YL-A8SQ | 0.901 | 1 | 3 | 0 | 0 | 3 |
| TCGA-YL-A8SR | 0.584 | 1 | 1 | 0 | 0 | 2 |
| TCGA-YL-A9WH | 1.170 | 1 | 3 | 1 | 0 | 2 |
| TCGA-YL-A9WI | 3.449 | 1 | 3 | 1 | 0 | 2 |
| TCGA-YL-A9WJ | 0.208 | 0 | 4 | 1 | 0 | 3 |
| TCGA-YL-A9WK | 2.764 | 1 | 2 | 0 | 0 | 3 |
| TCGA-YL-A9WL | 2.027 | 0 | 2 | 0 | 0 | 3 |
| TCGA-YL-A9WX | 4.126 | 1 | 3 | 1 | 0 | 3 |
| TCGA-YL-A9WY | 2.096 | 0 | 3 | 1 | 0 | 3 |
| TCGA-ZG-A8QW | 0.137 | 1 | 3 | 1 | 0 | 2 |
| TCGA-ZG-A8QX | 0.411 | 0 | 1 | 0 | 0 | 2 |
| TCGA-ZG-A8QY | 0.093 | 1 | 2 | 1 | 0 | 2 |
| TCGA-ZG-A8QZ | 0.844 | 1 | 2 | 1 | 0 | 1 |
| TCGA-ZG-A9KY | 0.093 | 1 | 1 | 1 | 0 | 2 |
| TCGA-ZG-A9L0 | 0.186 | 1 | 3 | 1 | 0 | 1 |
| TCGA-ZG-A9L1 | 3.142 | 1 | 1 | 1 | 0 | 3 |
| TCGA-ZG-A9L2 | 0.493 | 1 | 3 | 0 | 0 | 3 |
| TCGA-ZG-A9L4 | 3.036 | 1 | 1 | 0 | 0 | 2 |
| TCGA-ZG-A9L5 | 2.340 | 0 | 2 | 1 | 0 | 3 |
| TCGA-ZG-A9L6 | 1.819 | 1 | 1 | 0 | 0 | 3 |
| TCGA-ZG-A9L9 | 0.140 | 1 | 2 | 1 | 0 | 3 |
| TCGA-ZG-A9LB | 1.329 | 1 | 2 | 0 | 0 | 3 |
| TCGA-ZG-A9LM | 0.332 | 1 | 2 | 1 | 0 | 1 |
| TCGA-ZG-A9LN | 0.315 | 0 | 1 | 1 | 0 | 3 |
| TCGA-ZG-A9LS | 0.578 | 1 | 2 | 1 | 0 | 3 |
| TCGA-ZG-A9LU | 1.504 | 1 | 2 | 0 | 0 | 3 |
| TCGA-ZG-A9LY | 0.786 | 1 | 2 | 1 | 0 | 1 |
| TCGA-ZG-A9LZ | 0.849 | 1 | 3 | 0 | 0 | 3 |
| TCGA-ZG-A9M4 | 0.611 | 1 | 2 | 1 | 0 | 3 |
| TCGA-ZG-A9MC | 1.173 | 1 | 2 | 1 | 0 | 3 |
| TCGA-ZG-A9N3 | 0.668 | 1 | 1 | 1 | 0 | 3 |
| TCGA-ZG-A9ND | 0.715 | 0 | 1 | 0 | 0 | 1 |
| TCGA-ZG-A9NI | 0.362 | 1 | 2 | 0 | 0 | 1 |

Gleason (1:＜7;2:=7;3:＞7) Risk

1 LOW

1 LOW

3 HIGH

3 LOW

1. LOW
2. LOW

2 HIGH

2 LOW

1. LOW
2. LOW

2 LOW

2 LOW

1 LOW

1 HIGH

1 HIGH

3 HIGH

1. LOW
2. LOW

1 LOW

1. HIGH
2. HIGH
3. LOW

3 LOW

3 HIGH

3 LOW

3 HIGH

3 HIGH

3 LOW

2 LOW

2 HIGH

1. HIGH
2. LOW

3 LOW

1. HIGH
2. HIGH

3 LOW

1. LOW
2. HIGH

1 LOW

3 HIGH

2 HIGH

2 LOW

1. HIGH
2. LOW

3 HIGH

3 HIGH

3 LOW

3 LOW

1. LOW
2. HIGH
3. LOW
4. LOW

3 HIGH

3 HIGH

3 HIGH

3 HIGH

1. HIGH
2. HIGH
3. LOW

2 HIGH

1. LOW
2. HIGH

1 LOW

1. LOW
2. LOW
3. HIGH
4. HIGH

2 LOW

1. HIGH
2. HIGH
3. HIGH

2 HIGH

2 HIGH

1. HIGH
2. HIGH

1 HIGH

1 HIGH

3 HIGH

2 HIGH

2 LOW

1. LOW
2. LOW
3. LOW

3 LOW

2 LOW

2 HIGH

1. HIGH
2. HIGH
3. HIGH
4. LOW

3 LOW

2 HIGH

1. HIGH
2. LOW

3 HIGH

2 LOW

2 HIGH

2 LOW

1. HIGH
2. HIGH

2 LOW

2 LOW

2 LOW

2 HIGH

1. HIGH
2. HIGH
3. HIGH
4. HIGH

3 HIGH

3 HIGH

3 HIGH

1. HIGH
2. HIGH
3. HIGH
4. LOW

2 HIGH

1 HIGH

3 LOW

3 HIGH

3 HIGH

1. HIGH
2. LOW

3 LOW

1 HIGH

3 LOW

3 LOW

3 HIGH

1. LOW
2. HIGH
3. HIGH

1 HIGH

3 HIGH

3 HIGH

1. LOW
2. HIGH

2 LOW

1. HIGH
2. HIGH

2 LOW

1 LOW

3 LOW

1. HIGH
2. LOW
3. HIGH
4. LOW

2 HIGH

1. HIGH
2. LOW
3. LOW
4. LOW

2 LOW

2 LOW

1. LOW
2. HIGH

2 LOW

2 LOW

1 LOW

3 LOW

1 LOW

3 LOW

3 LOW

1. LOW
2. LOW

2 LOW

2 LOW

1. LOW
2. LOW
3. LOW

3 LOW

1 LOW

3 LOW

3 LOW

1 LOW

1. LOW
2. HIGH

2 LOW

1. LOW
2. LOW

3 LOW

3 LOW

3 LOW

1. LOW
2. LOW
3. LOW
4. HIGH

1 LOW

3 LOW

1. LOW
2. HIGH

3 HIGH

2 HIGH

2 LOW

1. LOW
2. LOW

2 HIGH

2 HIGH

1 LOW

3 LOW

1 HIGH

3 LOW

1. HIGH
2. LOW

1 HIGH

1 HIGH

3 HIGH

2 HIGH

1. LOW
2. LOW

2 HIGH

2 HIGH

2 HIGH

1 HIGH

1. HIGH
2. HIGH
3. HIGH

2 HIGH

2 LOW

1. LOW
2. LOW

2 LOW

1. LOW
2. LOW

1 LOW

3 HIGH

1. LOW
2. HIGH

3 LOW

3 HIGH

2 LOW

2 HIGH

1. HIGH
2. HIGH
3. HIGH
4. HIGH
5. HIGH
6. LOW
7. LOW
8. LOW
9. HIGH

3 HIGH

3 HIGH

3 HIGH

1. LOW
2. HIGH
3. HIGH

2 HIGH

1. LOW
2. LOW

2 HIGH

2 LOW

2 HIGH

1. HIGH
2. LOW

3 HIGH

1. LOW
2. HIGH

3 HIGH

2 HIGH

1. LOW
2. LOW

3 HIGH

3 HIGH

1 LOW

3 LOW

1. LOW
2. LOW

2 HIGH

2 LOW

2 LOW

2 HIGH

1. HIGH
2. LOW
3. HIGH
4. LOW
5. HIGH
6. HIGH

2 LOW

1. HIGH
2. LOW

2 LOW

2 LOW

1. LOW
2. LOW

1 LOW

3 LOW

3 LOW

3 HIGH

3 HIGH

3 HIGH

3 LOW

3 HIGH

1. LOW
2. LOW

2 HIGH

1. LOW
2. LOW

3 HIGH

1. LOW
2. LOW

2 HIGH

2 LOW

1 LOW

1. LOW
2. LOW
3. LOW

3 LOW

3 LOW

3 LOW

1. HIGH
2. HIGH

3 HIGH

1. HIGH
2. LOW

3 LOW

3 LOW

1. LOW
2. HIGH

3 LOW

3 HIGH

3 LOW

3 HIGH

1. LOW
2. LOW

3 LOW

3 LOW

3 LOW

2 HIGH

1. LOW
2. LOW

2 HIGH

2 LOW

1. HIGH
2. HIGH

3 LOW

3 HIGH

3 LOW

3 HIGH

3 LOW

3 HIGH

3 LOW

3 LOW

1. LOW
2. HIGH

3 HIGH

3 LOW

1. HIGH
2. LOW

3 HIGH

1. HIGH
2. HIGH
3. HIGH
4. HIGH
5. HIGH

3 LOW

1. HIGH
2. LOW
3. HIGH
4. LOW

2 LOW

1 HIGH

1. HIGH
2. LOW
3. HIGH

2 LOW

1. LOW
2. HIGH

2 LOW

1 HIGH

1. LOW
2. LOW

2 LOW

2 LOW

1. LOW
2. LOW

1 HIGH

3 HIGH

3 HIGH

1 HIGH

3 HIGH

3 LOW

1 HIGH

3 HIGH

1. HIGH
2. HIGH

3 HIGH

2 LOW

1. HIGH
2. LOW
3. HIGH
4. HIGH

3 LOW

3 HIGH

2 HIGH

1. HIGH
2. HIGH

3 HIGH

3 LOW

3 HIGH

2 LOW

1. LOW
2. HIGH

2 LOW

1. LOW
2. LOW

2 LOW

1. LOW
2. LOW

1 HIGH

3 HIGH

2 LOW

2 HIGH

1. HIGH
2. LOW
3. HIGH
4. LOW

2 HIGH

1 HIGH

3 HIGH

3 LOW

3 LOW

3 LOW

2 HIGH

2 LOW

2 LOW

1. LOW
2. LOW

3 HIGH

2 HIGH

1. LOW
2. HIGH

3 HIGH

3 HIGH

3 HIGH

2 HIGH

1. HIGH
2. HIGH

3 HIGH

3 LOW

3 HIGH

3 LOW

3 HIGH

3 HIGH

1. HIGH
2. HIGH
3. HIGH

3 HIGH

3 HIGH

1. HIGH
2. HIGH

3 LOW

3 HIGH

3 HIGH

3 HIGH

3 HIGH

3 HIGH

3 HIGH

1. LOW
2. HIGH

3 LOW

3 HIGH

3 HIGH

3 HIGH

3 HIGH

3 HIGH

3 HIGH

3 HIGH

1. LOW
2. HIGH

3 HIGH

3 HIGH

3 HIGH

3 LOW

1 LOW

3 HIGH

3 LOW

3 HIGH

3 HIGH

3 LOW

3 LOW

3 HIGH

3 HIGH

3 LOW

3 LOW

3 LOW

3 LOW

3 LOW

3 LOW

3 LOW

3 LOW

3 HIGH

3 HIGH

3 HIGH

3 LOW

3 LOW

3 LOW
