# Supplementary material for: Identification of ZDHHC1 as a Pyroptosis Inducer and Potential Target in the Establishment of Pyroptosis-Related Signature in Localized Prostate Cancer
Source: Oxid Med Cell Longev. 2022 Dec 22;2022:5925817. doi: 10.1155/2022/5925817 (PMC9800907; doi:10.1155/2022/5925817)
Supplement: Supplementary 7 — Supplementary Table 7: associations between risk groups with 16 immune cell types and activity in 13 immune-related pathways. [file 5925817.f7.docx]

| ID | aDCs | APC_co_inhibition | APC_co_stimulation | B_cells |
| --- | --- | --- | --- | --- |
| CM.4-0014 | 0.251 | 0.198 | 0.235 | 0.181 |
| CM.4-0028 | 0.266 | 0.239 | 0.207 | 0.233 |
| CM.4-0054 | 0.258 | 0.260 | 0.218 | 0.236 |
| CM.4-0055 | 0.342 | 0.287 | 0.236 | 0.220 |
| CM.4-0062 | 0.249 | 0.273 | 0.195 | 0.275 |
| CM.4-0074 | 0.328 | 0.268 | 0.249 | 0.286 |
| CM.4-0078 | 0.310 | 0.261 | 0.218 | 0.231 |
| CM.4-0080 | 0.389 | 0.284 | 0.244 | 0.544 |
| CM.4-0082 | 0.272 | 0.170 | 0.226 | 0.183 |
| CM.4-0093 | 0.280 | 0.296 | 0.235 | 0.215 |
| CM.4-0097 | 0.321 | 0.235 | 0.240 | 0.276 |
| CM.1-0028.1 | 0.224 | 0.327 | 0.258 | 0.237 |
| CM.4-0019 | 0.325 | 0.229 | 0.298 | 0.199 |
| CM.4-0064 | 0.269 | 0.233 | 0.243 | 0.206 |
| CM.4-0077 | 0.348 | 0.225 | 0.207 | 0.211 |
| CM.4-0079 | 0.350 | 0.256 | 0.252 | 0.217 |
| CM.4-0047 | 0.242 | 0.355 | 0.251 | 0.224 |
| CM.4-0048 | 0.319 | 0.305 | 0.312 | 0.249 |
| CM.4-0049 | 0.346 | 0.272 | 0.286 | 0.249 |
| CM.4-0050 | 0.274 | 0.245 | 0.269 | 0.252 |
| CM.4-0051 | 0.373 | 0.195 | 0.240 | 0.181 |
| CM.4-0052 | 0.492 | 0.340 | 0.317 | 0.305 |
| CM.4-0061 | 0.353 | 0.247 | 0.225 | 0.272 |
| CM.4-0076 | 0.270 | 0.243 | 0.190 | 0.206 |
| CM.4-0083 | 0.363 | 0.373 | 0.267 | 0.289 |
| CM.4-0087 | 0.359 | 0.235 | 0.218 | 0.237 |
| CM.4-0092 | 0.398 | 0.319 | 0.259 | 0.257 |
| CM.4-0094 | 0.361 | 0.285 | 0.271 | 0.263 |
| CM.4-0013 | 0.250 | 0.268 | 0.231 | 0.196 |
| CM.4-0084 | 0.483 | 0.244 | 0.287 | 0.324 |
| CM.4-0067 | 0.412 | 0.240 | 0.267 | 0.251 |
| CM.4-0068 | 0.342 | 0.249 | 0.289 | 0.237 |
| CM.4-0070 | 0.433 | 0.317 | 0.283 | 0.320 |
| CM.4-0090 | 0.281 | 0.286 | 0.262 | 0.260 |
| CM.4-0098 | 0.356 | 0.249 | 0.220 | 0.216 |
| CM.4-0045 | 0.317 | 0.211 | 0.266 | 0.292 |
| CM.4-0075 | 0.319 | 0.277 | 0.271 | 0.311 |
| CM.4-0081 | 0.319 | 0.333 | 0.222 | 0.228 |
| CM.4-0091 | 0.267 | 0.300 | 0.205 | 0.203 |
| PT184 | 0.305 | 0.255 | 0.285 | 0.269 |
| PT127 | 0.201 | 0.243 | 0.241 | 0.221 |
| PT168 | 0.295 | 0.245 | 0.251 | 0.274 |
| PT199 | 0.263 | 0.256 | 0.239 | 0.232 |
| PT236 | 0.268 | 0.351 | 0.222 | 0.210 |
| PT243 | 0.413 | 0.320 | 0.273 | 0.273 |
| CM.4-0066 | 0.487 | 0.292 | 0.282 | 0.234 |
| CM.4-0095 | 0.363 | 0.220 | 0.229 | 0.242 |
| PT081 | 0.282 | 0.294 | 0.255 | 0.354 |
| CMO10311-0038 | 0.430 | 0.415 | 0.356 | 0.376 |

| CMO10311-0042 | 0.257 | 0.288 | 0.244 | 0.214 |
| --- | --- | --- | --- | --- |
| CM.1-0003.1 | 0.299 | 0.285 | 0.220 | 0.266 |
| CM.1-0004.1 | 0.256 | 0.311 | 0.235 | 0.275 |
| CM.1-0017.1 | 0.300 | 0.265 | 0.275 | 0.218 |
| CM.1-0025.1 | 0.435 | 0.375 | 0.343 | 0.237 |
| CM.4-0004 | 0.277 | 0.290 | 0.210 | 0.307 |
| CM.4-0042 | 0.230 | 0.193 | 0.216 | 0.236 |
| CM.4-0043 | 0.308 | 0.252 | 0.250 | 0.247 |
| CM.4-0044 | 0.270 | 0.191 | 0.211 | 0.220 |
| CM.4-0046 | 0.266 | 0.263 | 0.207 | 0.307 |
| CM.4-0065 | 0.417 | 0.385 | 0.272 | 0.399 |
| CM.4-0096 | 0.407 | 0.262 | 0.294 | 0.215 |
| CM.1-0001.1 | 0.292 | 0.301 | 0.234 | 0.237 |
| CM.1-0005.1 | 0.299 | 0.379 | 0.279 | 0.255 |
| CM.1-0016.1 | 0.227 | 0.343 | 0.263 | 0.306 |
| CM.1-0019.1 | 0.224 | 0.285 | 0.213 | 0.244 |
| CM.1-0021.1 | 0.401 | 0.312 | 0.268 | 0.307 |
| CM.1-0030.1 | 0.285 | 0.277 | 0.222 | 0.214 |
| CM.4-0085 | 0.295 | 0.231 | 0.234 | 0.224 |
| CM.4-0088 | 0.296 | 0.321 | 0.271 | 0.252 |
| CM.1-0012.1 | 0.245 | 0.333 | 0.279 | 0.276 |
| CM.1-0002.1 | 0.361 | 0.358 | 0.244 | 0.194 |
| CM.1-0013.1 | 0.251 | 0.380 | 0.230 | 0.223 |
| CM.1-0015.1 | 0.464 | 0.322 | 0.248 | 0.334 |
| CM.1-0018.1 | 0.349 | 0.230 | 0.254 | 0.247 |
| CM.1-0020.1 | 0.372 | 0.190 | 0.268 | 0.232 |
| CM.1-0022.1 | 0.300 | 0.269 | 0.214 | 0.312 |
| CM.1-0027.1 | 0.336 | 0.247 | 0.220 | 0.206 |
| CM.4-0086 | 0.244 | 0.370 | 0.266 | 0.325 |
| CM.4-0034 | 0.207 | 0.399 | 0.248 | 0.262 |
| CM.4-0053 | 0.250 | 0.242 | 0.197 | 0.245 |
| CM.4-0009 | 0.397 | 0.201 | 0.221 | 0.272 |
| CM.4-0024 | 0.217 | 0.238 | 0.186 | 0.288 |
| CM.4-0037 | 0.449 | 0.404 | 0.298 | 0.307 |
| CM.4-0041 | 0.653 | 0.261 | 0.314 | 0.324 |
| CM.4-0071 | 0.496 | 0.185 | 0.259 | 0.211 |
| CM.4-0099 | 0.358 | 0.235 | 0.242 | 0.243 |
| CM.1-0006.1 | 0.370 | 0.324 | 0.277 | 0.286 |
| CM.1-0008.1 | 0.247 | 0.365 | 0.223 | 0.251 |
| CM.1-0023.1 | 0.283 | 0.278 | 0.259 | 0.236 |
| CM.1-0026.1 | 0.295 | 0.333 | 0.290 | 0.240 |
| CM.4-0021 | 0.367 | 0.316 | 0.249 | 0.285 |
| CM.4-0020 | 0.307 | 0.201 | 0.227 | 0.230 |
| CM.4-0011 | 0.314 | 0.344 | 0.234 | 0.229 |
| PT197 | 0.378 | 0.276 | 0.254 | 0.219 |
| PT220 | 0.373 | 0.271 | 0.251 | 0.297 |
| PT264 | 0.336 | 0.271 | 0.246 | 0.180 |
| CMO10311-0043 | 0.365 | 0.416 | 0.276 | 0.339 |
| CMO10311-0009 | 0.277 | 0.267 | 0.202 | 0.218 |
| CMO10311-0010 | 0.270 | 0.276 | 0.218 | 0.324 |

| CMO10311-0008 | 0.303 | 0.324 | 0.254 | 0.200 |
| --- | --- | --- | --- | --- |
| CMO10311-0011 | 0.342 | 0.368 | 0.288 | 0.248 |
| CMO10311-0007 | 0.291 | 0.303 | 0.237 | 0.271 |
| CMO10311-0013 | 0.385 | 0.321 | 0.261 | 0.237 |
| CMO10311-0012 | 0.416 | 0.318 | 0.304 | 0.316 |

| CCR | CD8+_T_cells | Check-point | Cytolytic_activity | DCs | HLA |
| --- | --- | --- | --- | --- | --- |
| 0.309 | 0.294 | 0.268 | 0.385 | 0.209 | 0.635 |
| 0.290 | 0.322 | 0.245 | 0.296 | 0.176 | 0.680 |
| 0.317 | 0.316 | 0.234 | 0.324 | 0.154 | 0.666 |
| 0.329 | 0.499 | 0.286 | 0.348 | 0.170 | 0.750 |
| 0.297 | 0.235 | 0.235 | 0.303 | 0.177 | 0.701 |
| 0.321 | 0.331 | 0.253 | 0.334 | 0.167 | 0.734 |
| 0.303 | 0.429 | 0.256 | 0.411 | 0.156 | 0.712 |
| 0.349 | 0.536 | 0.292 | 0.609 | 0.175 | 0.778 |
| 0.302 | 0.227 | 0.256 | 0.259 | 0.136 | 0.626 |
| 0.337 | 0.497 | 0.258 | 0.465 | 0.191 | 0.697 |
| 0.301 | 0.320 | 0.244 | 0.318 | 0.187 | 0.684 |
| 0.346 | 0.414 | 0.274 | 0.274 | 0.188 | 0.759 |
| 0.322 | 0.368 | 0.278 | 0.263 | 0.181 | 0.686 |
| 0.297 | 0.162 | 0.239 | 0.304 | 0.184 | 0.664 |
| 0.317 | 0.553 | 0.238 | 0.426 | 0.153 | 0.668 |
| 0.330 | 0.327 | 0.259 | 0.315 | 0.190 | 0.715 |
| 0.321 | 0.314 | 0.304 | 0.341 | 0.293 | 0.602 |
| 0.383 | 0.351 | 0.325 | 0.536 | 0.402 | 0.757 |
| 0.332 | 0.325 | 0.311 | 0.264 | 0.198 | 0.648 |
| 0.353 | 0.230 | 0.291 | 0.486 | 0.207 | 0.731 |
| 0.304 | 0.558 | 0.279 | 0.379 | 0.184 | 0.718 |
| 0.365 | 0.174 | 0.340 | 0.279 | 0.278 | 0.722 |
| 0.324 | 0.234 | 0.279 | 0.270 | 0.149 | 0.695 |
| 0.288 | 0.293 | 0.234 | 0.367 | 0.195 | 0.663 |
| 0.359 | 0.585 | 0.320 | 0.521 | 0.199 | 0.761 |
| 0.329 | 0.329 | 0.249 | 0.378 | 0.143 | 0.663 |
| 0.356 | 0.495 | 0.290 | 0.533 | 0.173 | 0.726 |
| 0.324 | 0.338 | 0.276 | 0.320 | 0.178 | 0.666 |
| 0.331 | 0.247 | 0.273 | 0.546 | 0.231 | 0.699 |
| 0.374 | 0.500 | 0.317 | 0.449 | 0.160 | 0.751 |
| 0.363 | 0.328 | 0.312 | 0.353 | 0.202 | 0.664 |
| 0.345 | 0.325 | 0.280 | 0.401 | 0.196 | 0.693 |
| 0.359 | 0.539 | 0.309 | 0.469 | 0.191 | 0.762 |
| 0.337 | 0.315 | 0.286 | 0.292 | 0.206 | 0.679 |
| 0.311 | 0.339 | 0.253 | 0.373 | 0.172 | 0.670 |
| 0.315 | 0.351 | 0.258 | 0.285 | 0.231 | 0.720 |
| 0.356 | 0.155 | 0.281 | 0.348 | 0.137 | 0.683 |
| 0.327 | 0.332 | 0.256 | 0.290 | 0.171 | 0.671 |
| 0.315 | 0.261 | 0.263 | 0.325 | 0.190 | 0.663 |
| 0.323 | 0.290 | 0.263 | 0.330 | 0.206 | 0.697 |
| 0.304 | 0.305 | 0.261 | 0.377 | 0.232 | 0.678 |
| 0.308 | 0.291 | 0.255 | 0.303 | 0.248 | 0.693 |
| 0.323 | 0.396 | 0.249 | 0.409 | 0.171 | 0.710 |
| 0.326 | 0.528 | 0.270 | 0.419 | 0.219 | 0.731 |
| 0.341 | 0.463 | 0.290 | 0.567 | 0.165 | 0.741 |
| 0.357 | 0.532 | 0.330 | 0.254 | 0.189 | 0.716 |
| 0.310 | 0.259 | 0.255 | 0.319 | 0.211 | 0.661 |
| 0.309 | 0.473 | 0.284 | 0.422 | 0.247 | 0.657 |
| 0.403 | 0.541 | 0.385 | 0.568 | 0.335 | 0.814 |

| 0.329 | 0.369 | 0.257 | 0.380 | 0.211 | 0.694 |
| --- | --- | --- | --- | --- | --- |
| 0.295 | 0.267 | 0.253 | 0.357 | 0.175 | 0.707 |
| 0.344 | 0.411 | 0.269 | 0.359 | 0.194 | 0.744 |
| 0.327 | 0.243 | 0.258 | 0.412 | 0.152 | 0.694 |
| 0.352 | 0.301 | 0.322 | 0.481 | 0.217 | 0.797 |
| 0.302 | 0.288 | 0.256 | 0.367 | 0.240 | 0.643 |
| 0.298 | 0.167 | 0.261 | 0.357 | 0.152 | 0.696 |
| 0.311 | 0.312 | 0.258 | 0.314 | 0.219 | 0.657 |
| 0.286 | 0.289 | 0.217 | 0.338 | 0.092 | 0.640 |
| 0.292 | 0.290 | 0.234 | 0.294 | 0.087 | 0.687 |
| 0.363 | 0.558 | 0.333 | 0.480 | 0.366 | 0.833 |
| 0.345 | 0.473 | 0.271 | 0.339 | 0.202 | 0.719 |
| 0.324 | 0.473 | 0.273 | 0.483 | 0.261 | 0.798 |
| 0.383 | 0.455 | 0.308 | 0.394 | 0.190 | 0.819 |
| 0.343 | 0.352 | 0.298 | 0.481 | 0.222 | 0.790 |
| 0.327 | 0.222 | 0.261 | 0.325 | 0.234 | 0.728 |
| 0.367 | 0.369 | 0.290 | 0.310 | 0.263 | 0.757 |
| 0.310 | 0.291 | 0.237 | 0.345 | 0.184 | 0.705 |
| 0.317 | 0.351 | 0.260 | 0.378 | 0.169 | 0.692 |
| 0.368 | 0.604 | 0.288 | 0.473 | 0.173 | 0.759 |
| 0.329 | 0.370 | 0.282 | 0.342 | 0.137 | 0.758 |
| 0.330 | 0.252 | 0.283 | 0.315 | 0.224 | 0.761 |
| 0.325 | 0.174 | 0.259 | 0.273 | 0.165 | 0.656 |
| 0.345 | 0.314 | 0.302 | 0.287 | 0.304 | 0.743 |
| 0.331 | 0.338 | 0.266 | 0.302 | 0.204 | 0.717 |
| 0.335 | 0.413 | 0.288 | 0.282 | 0.282 | 0.696 |
| 0.316 | 0.401 | 0.256 | 0.470 | 0.204 | 0.718 |
| 0.337 | 0.319 | 0.279 | 0.267 | 0.182 | 0.707 |
| 0.318 | 0.685 | 0.277 | 0.254 | 0.271 | 0.749 |
| 0.335 | 0.307 | 0.321 | 0.328 | 0.204 | 0.731 |
| 0.306 | 0.225 | 0.220 | 0.297 | 0.260 | 0.702 |
| 0.296 | 0.524 | 0.264 | 0.539 | 0.142 | 0.689 |
| 0.313 | 0.355 | 0.259 | 0.304 | 0.173 | 0.679 |
| 0.376 | 0.711 | 0.324 | 0.676 | 0.310 | 0.814 |
| 0.337 | 0.294 | 0.353 | 0.327 | 0.327 | 0.586 |
| 0.301 | 0.196 | 0.251 | 0.320 | 0.167 | 0.691 |
| 0.333 | 0.375 | 0.283 | 0.362 | 0.208 | 0.684 |
| 0.369 | 0.294 | 0.297 | 0.332 | 0.175 | 0.782 |
| 0.335 | 0.414 | 0.266 | 0.384 | 0.203 | 0.721 |
| 0.318 | 0.278 | 0.264 | 0.339 | 0.132 | 0.640 |
| 0.327 | 0.338 | 0.277 | 0.339 | 0.126 | 0.701 |
| 0.342 | 0.354 | 0.288 | 0.320 | 0.178 | 0.668 |
| 0.289 | 0.312 | 0.247 | 0.300 | 0.150 | 0.661 |
| 0.351 | 0.474 | 0.306 | 0.539 | 0.240 | 0.789 |
| 0.333 | 0.341 | 0.272 | 0.303 | 0.251 | 0.717 |
| 0.333 | 0.359 | 0.286 | 0.350 | 0.282 | 0.693 |
| 0.326 | 0.242 | 0.312 | 0.310 | 0.320 | 0.685 |
| 0.380 | 0.579 | 0.341 | 0.638 | 0.370 | 0.802 |
| 0.288 | 0.173 | 0.243 | 0.317 | 0.199 | 0.691 |
| 0.343 | 0.411 | 0.283 | 0.312 | 0.178 | 0.704 |

| 0.335 | 0.302 | 0.276 | 0.353 | 0.174 | 0.738 |
| --- | --- | --- | --- | --- | --- |
| 0.382 | 0.410 | 0.299 | 0.423 | 0.191 | 0.810 |
| 0.324 | 0.437 | 0.277 | 0.494 | 0.226 | 0.782 |
| 0.361 | 0.331 | 0.289 | 0.406 | 0.217 | 0.778 |
| 0.349 | 0.338 | 0.306 | 0.447 | 0.217 | 0.725 |

| iDCs | Inflammation-promoting | Macrophages | Mast_cells | MHC_class_I | Neutrophils |
| --- | --- | --- | --- | --- | --- |
| 0.084 | 0.388 | 0.394 | 0.204 | 0.945 | 0.424 |
| 0.202 | 0.334 | 0.466 | 0.229 | 0.911 | 0.387 |
| 0.105 | 0.345 | 0.457 | 0.313 | 0.913 | 0.410 |
| 0.093 | 0.426 | 0.462 | 0.415 | 0.971 | 0.430 |
| 0.095 | 0.305 | 0.522 | 0.223 | 0.905 | 0.399 |
| 0.150 | 0.397 | 0.482 | 0.283 | 0.970 | 0.440 |
| 0.090 | 0.428 | 0.453 | 0.263 | 0.947 | 0.412 |
| 0.108 | 0.484 | 0.475 | 0.381 | 0.976 | 0.455 |
| 0.081 | 0.317 | 0.456 | 0.333 | 0.901 | 0.443 |
| 0.113 | 0.376 | 0.476 | 0.235 | 0.969 | 0.435 |
| 0.132 | 0.346 | 0.457 | 0.351 | 0.939 | 0.432 |
| 0.092 | 0.349 | 0.485 | 0.344 | 0.960 | 0.439 |
| 0.086 | 0.352 | 0.393 | 0.227 | 0.897 | 0.436 |
| 0.088 | 0.279 | 0.456 | 0.238 | 0.906 | 0.393 |
| 0.230 | 0.401 | 0.513 | 0.274 | 0.968 | 0.423 |
| 0.099 | 0.376 | 0.512 | 0.394 | 0.956 | 0.436 |
| 0.067 | 0.396 | 0.410 | 0.314 | 0.920 | 0.415 |
| 0.208 | 0.506 | 0.478 | 0.311 | 0.990 | 0.493 |
| 0.217 | 0.317 | 0.436 | 0.340 | 0.902 | 0.447 |
| 0.256 | 0.418 | 0.519 | 0.413 | 0.927 | 0.542 |
| 0.093 | 0.369 | 0.448 | 0.325 | 0.888 | 0.462 |
| 0.105 | 0.466 | 0.445 | 0.229 | 0.914 | 0.398 |
| 0.264 | 0.353 | 0.488 | 0.227 | 0.947 | 0.471 |
| 0.089 | 0.330 | 0.408 | 0.295 | 0.928 | 0.378 |
| 0.240 | 0.454 | 0.539 | 0.336 | 0.987 | 0.456 |
| 0.105 | 0.350 | 0.438 | 0.272 | 0.945 | 0.403 |
| 0.145 | 0.407 | 0.509 | 0.592 | 0.942 | 0.474 |
| 0.113 | 0.373 | 0.494 | 0.322 | 0.914 | 0.462 |
| 0.091 | 0.359 | 0.508 | 0.487 | 0.941 | 0.431 |
| 0.139 | 0.393 | 0.451 | 0.409 | 0.966 | 0.455 |
| 0.133 | 0.393 | 0.491 | 0.382 | 0.931 | 0.496 |
| 0.085 | 0.383 | 0.466 | 0.292 | 0.917 | 0.469 |
| 0.113 | 0.380 | 0.514 | 0.384 | 0.956 | 0.511 |
| 0.134 | 0.333 | 0.486 | 0.363 | 0.964 | 0.491 |
| 0.089 | 0.337 | 0.474 | 0.354 | 0.892 | 0.420 |
| 0.187 | 0.348 | 0.479 | 0.454 | 0.918 | 0.413 |
| 0.090 | 0.376 | 0.447 | 0.352 | 0.948 | 0.517 |
| 0.084 | 0.400 | 0.505 | 0.252 | 0.978 | 0.451 |
| 0.086 | 0.321 | 0.454 | 0.245 | 0.894 | 0.402 |
| 0.230 | 0.381 | 0.470 | 0.438 | 0.939 | 0.441 |
| 0.092 | 0.368 | 0.430 | 0.436 | 0.895 | 0.428 |
| 0.158 | 0.319 | 0.459 | 0.353 | 0.930 | 0.386 |
| 0.163 | 0.383 | 0.510 | 0.300 | 0.938 | 0.397 |
| 0.193 | 0.460 | 0.488 | 0.372 | 0.975 | 0.456 |
| 0.139 | 0.467 | 0.437 | 0.285 | 0.988 | 0.457 |
| 0.142 | 0.433 | 0.449 | 0.490 | 0.962 | 0.514 |
| 0.102 | 0.342 | 0.416 | 0.206 | 0.954 | 0.439 |
| 0.117 | 0.480 | 0.479 | 0.278 | 0.908 | 0.417 |
| 0.202 | 0.497 | 0.596 | 0.453 | 0.987 | 0.502 |

| 0.182 | 0.361 | 0.478 | 0.433 | 0.946 | 0.402 |
| --- | --- | --- | --- | --- | --- |
| 0.103 | 0.296 | 0.427 | 0.305 | 0.932 | 0.341 |
| 0.095 | 0.378 | 0.438 | 0.319 | 0.939 | 0.415 |
| 0.097 | 0.304 | 0.506 | 0.278 | 0.925 | 0.414 |
| 0.150 | 0.420 | 0.542 | 0.294 | 0.997 | 0.430 |
| 0.105 | 0.307 | 0.459 | 0.241 | 0.907 | 0.394 |
| 0.092 | 0.327 | 0.465 | 0.342 | 0.935 | 0.419 |
| 0.101 | 0.354 | 0.430 | 0.292 | 0.944 | 0.406 |
| 0.107 | 0.348 | 0.411 | 0.207 | 0.934 | 0.366 |
| 0.167 | 0.287 | 0.422 | 0.227 | 0.917 | 0.377 |
| 0.090 | 0.459 | 0.551 | 0.155 | 0.980 | 0.507 |
| 0.165 | 0.369 | 0.500 | 0.349 | 0.962 | 0.433 |
| 0.110 | 0.422 | 0.504 | 0.345 | 0.982 | 0.419 |
| 0.098 | 0.405 | 0.547 | 0.326 | 0.980 | 0.506 |
| 0.184 | 0.424 | 0.531 | 0.337 | 0.976 | 0.413 |
| 0.131 | 0.304 | 0.459 | 0.352 | 0.902 | 0.435 |
| 0.156 | 0.426 | 0.514 | 0.249 | 0.973 | 0.461 |
| 0.124 | 0.317 | 0.474 | 0.241 | 0.922 | 0.388 |
| 0.151 | 0.360 | 0.472 | 0.312 | 0.961 | 0.420 |
| 0.130 | 0.412 | 0.504 | 0.206 | 0.979 | 0.472 |
| 0.090 | 0.375 | 0.514 | 0.287 | 0.947 | 0.477 |
| 0.099 | 0.350 | 0.526 | 0.395 | 0.959 | 0.414 |
| 0.204 | 0.316 | 0.433 | 0.395 | 0.962 | 0.379 |
| 0.332 | 0.392 | 0.543 | 0.367 | 0.940 | 0.423 |
| 0.106 | 0.328 | 0.498 | 0.385 | 0.925 | 0.441 |
| 0.081 | 0.425 | 0.476 | 0.500 | 0.949 | 0.515 |
| 0.189 | 0.339 | 0.483 | 0.214 | 0.896 | 0.409 |
| 0.132 | 0.383 | 0.483 | 0.519 | 0.964 | 0.418 |
| 0.091 | 0.409 | 0.547 | 0.318 | 0.969 | 0.455 |
| 0.213 | 0.368 | 0.448 | 0.230 | 0.930 | 0.393 |
| 0.093 | 0.314 | 0.412 | 0.305 | 0.926 | 0.368 |
| 0.098 | 0.378 | 0.469 | 0.244 | 0.915 | 0.432 |
| 0.083 | 0.330 | 0.496 | 0.229 | 0.913 | 0.454 |
| 0.081 | 0.482 | 0.541 | 0.414 | 1.000 | 0.474 |
| 0.520 | 0.469 | 0.458 | 0.323 | 0.866 | 0.443 |
| 0.181 | 0.354 | 0.497 | 0.326 | 0.921 | 0.431 |
| 0.102 | 0.346 | 0.481 | 0.264 | 0.937 | 0.421 |
| 0.189 | 0.374 | 0.615 | 0.338 | 0.957 | 0.450 |
| 0.100 | 0.367 | 0.488 | 0.299 | 0.948 | 0.404 |
| 0.093 | 0.343 | 0.456 | 0.362 | 0.921 | 0.428 |
| 0.093 | 0.434 | 0.510 | 0.363 | 0.966 | 0.427 |
| 0.094 | 0.411 | 0.480 | 0.198 | 0.979 | 0.421 |
| 0.136 | 0.344 | 0.440 | 0.323 | 0.935 | 0.388 |
| 0.204 | 0.383 | 0.556 | 0.383 | 0.957 | 0.494 |
| 0.153 | 0.379 | 0.478 | 0.300 | 0.949 | 0.427 |
| 0.156 | 0.392 | 0.469 | 0.401 | 0.915 | 0.469 |
| 0.198 | 0.399 | 0.531 | 0.334 | 0.928 | 0.469 |
| 0.097 | 0.432 | 0.516 | 0.506 | 0.981 | 0.374 |
| 0.100 | 0.308 | 0.459 | 0.381 | 0.894 | 0.362 |
| 0.090 | 0.397 | 0.431 | 0.465 | 0.886 | 0.422 |

| 0.115 | 0.312 | 0.509 | 0.403 | 0.932 | 0.411 |
| --- | --- | --- | --- | --- | --- |
| 0.141 | 0.421 | 0.539 | 0.372 | 0.973 | 0.537 |
| 0.145 | 0.429 | 0.492 | 0.376 | 0.980 | 0.439 |
| 0.162 | 0.374 | 0.615 | 0.326 | 0.962 | 0.461 |
| 0.133 | 0.345 | 0.591 | 0.310 | 0.929 | 0.435 |

| NK_cells | Parainflammation | pDCs | T_cell_co-inhibition | T_cell_co-stimulation |
| --- | --- | --- | --- | --- |
| 0.052 | 0.563 | 0.272 | 0.188 | 0.226 |
| 0.070 | 0.565 | 0.304 | 0.169 | 0.139 |
| 0.053 | 0.541 | 0.265 | 0.153 | 0.146 |
| 0.095 | 0.591 | 0.295 | 0.198 | 0.236 |
| 0.065 | 0.574 | 0.299 | 0.176 | 0.149 |
| 0.112 | 0.588 | 0.295 | 0.202 | 0.223 |
| 0.060 | 0.555 | 0.289 | 0.198 | 0.249 |
| 0.133 | 0.586 | 0.300 | 0.232 | 0.258 |
| 0.052 | 0.577 | 0.269 | 0.178 | 0.207 |
| 0.124 | 0.596 | 0.287 | 0.216 | 0.186 |
| 0.122 | 0.581 | 0.282 | 0.174 | 0.193 |
| 0.143 | 0.602 | 0.313 | 0.177 | 0.176 |
| 0.058 | 0.544 | 0.295 | 0.228 | 0.176 |
| 0.059 | 0.498 | 0.271 | 0.172 | 0.182 |
| 0.103 | 0.579 | 0.276 | 0.204 | 0.178 |
| 0.142 | 0.600 | 0.298 | 0.225 | 0.197 |
| 0.036 | 0.564 | 0.291 | 0.173 | 0.287 |
| 0.040 | 0.674 | 0.350 | 0.317 | 0.333 |
| 0.058 | 0.537 | 0.275 | 0.198 | 0.328 |
| 0.027 | 0.570 | 0.349 | 0.241 | 0.238 |
| 0.065 | 0.560 | 0.354 | 0.273 | 0.189 |
| 0.063 | 0.602 | 0.378 | 0.244 | 0.266 |
| 0.054 | 0.585 | 0.325 | 0.201 | 0.217 |
| 0.117 | 0.536 | 0.279 | 0.164 | 0.168 |
| 0.102 | 0.642 | 0.317 | 0.246 | 0.283 |
| 0.120 | 0.574 | 0.269 | 0.157 | 0.172 |
| 0.110 | 0.609 | 0.253 | 0.228 | 0.221 |
| 0.059 | 0.597 | 0.303 | 0.195 | 0.219 |
| 0.063 | 0.614 | 0.253 | 0.219 | 0.212 |
| 0.063 | 0.625 | 0.299 | 0.238 | 0.282 |
| 0.052 | 0.608 | 0.274 | 0.248 | 0.250 |
| 0.058 | 0.580 | 0.245 | 0.204 | 0.213 |
| 0.148 | 0.595 | 0.280 | 0.252 | 0.289 |
| 0.189 | 0.602 | 0.303 | 0.238 | 0.201 |
| 0.060 | 0.564 | 0.263 | 0.178 | 0.176 |
| 0.061 | 0.570 | 0.285 | 0.205 | 0.181 |
| 0.064 | 0.606 | 0.296 | 0.180 | 0.194 |
| 0.055 | 0.634 | 0.282 | 0.196 | 0.175 |
| 0.108 | 0.572 | 0.293 | 0.196 | 0.151 |
| 0.073 | 0.557 | 0.249 | 0.154 | 0.194 |
| 0.060 | 0.541 | 0.266 | 0.188 | 0.144 |
| 0.067 | 0.540 | 0.256 | 0.150 | 0.192 |
| 0.069 | 0.580 | 0.295 | 0.189 | 0.166 |
| 0.067 | 0.610 | 0.264 | 0.191 | 0.249 |
| 0.202 | 0.622 | 0.285 | 0.231 | 0.237 |
| 0.159 | 0.603 | 0.263 | 0.341 | 0.259 |
| 0.052 | 0.610 | 0.274 | 0.229 | 0.171 |
| 0.121 | 0.541 | 0.309 | 0.275 | 0.311 |
| 0.126 | 0.685 | 0.386 | 0.289 | 0.419 |

| 0.098 | 0.597 | 0.255 | 0.205 | 0.186 |
| --- | --- | --- | --- | --- |
| 0.074 | 0.545 | 0.307 | 0.158 | 0.195 |
| 0.123 | 0.570 | 0.279 | 0.137 | 0.235 |
| 0.067 | 0.589 | 0.297 | 0.199 | 0.174 |
| 0.067 | 0.678 | 0.327 | 0.208 | 0.276 |
| 0.074 | 0.559 | 0.305 | 0.183 | 0.210 |
| 0.062 | 0.566 | 0.273 | 0.274 | 0.174 |
| 0.070 | 0.555 | 0.281 | 0.179 | 0.176 |
| 0.076 | 0.601 | 0.282 | 0.124 | 0.127 |
| 0.128 | 0.605 | 0.302 | 0.134 | 0.161 |
| 0.103 | 0.700 | 0.389 | 0.297 | 0.348 |
| 0.155 | 0.628 | 0.286 | 0.185 | 0.212 |
| 0.073 | 0.584 | 0.322 | 0.196 | 0.240 |
| 0.108 | 0.649 | 0.359 | 0.221 | 0.289 |
| 0.071 | 0.597 | 0.296 | 0.210 | 0.257 |
| 0.079 | 0.574 | 0.256 | 0.164 | 0.195 |
| 0.079 | 0.620 | 0.306 | 0.229 | 0.247 |
| 0.165 | 0.583 | 0.280 | 0.172 | 0.157 |
| 0.069 | 0.586 | 0.281 | 0.169 | 0.203 |
| 0.111 | 0.644 | 0.310 | 0.241 | 0.266 |
| 0.235 | 0.576 | 0.292 | 0.197 | 0.249 |
| 0.072 | 0.592 | 0.299 | 0.151 | 0.226 |
| 0.084 | 0.613 | 0.344 | 0.133 | 0.244 |
| 0.118 | 0.600 | 0.309 | 0.258 | 0.238 |
| 0.172 | 0.599 | 0.304 | 0.214 | 0.198 |
| 0.038 | 0.574 | 0.266 | 0.172 | 0.192 |
| 0.137 | 0.565 | 0.280 | 0.195 | 0.184 |
| 0.058 | 0.608 | 0.315 | 0.171 | 0.220 |
| 0.171 | 0.610 | 0.299 | 0.217 | 0.209 |
| 0.217 | 0.557 | 0.362 | 0.261 | 0.251 |
| 0.065 | 0.548 | 0.253 | 0.133 | 0.144 |
| 0.163 | 0.532 | 0.306 | 0.224 | 0.180 |
| 0.053 | 0.550 | 0.296 | 0.170 | 0.188 |
| 0.073 | 0.688 | 0.317 | 0.250 | 0.286 |
| 0.000 | 0.578 | 0.296 | 0.318 | 0.261 |
| 0.063 | 0.530 | 0.293 | 0.149 | 0.221 |
| 0.088 | 0.577 | 0.293 | 0.216 | 0.206 |
| 0.067 | 0.626 | 0.345 | 0.194 | 0.246 |
| 0.073 | 0.623 | 0.308 | 0.214 | 0.183 |
| 0.066 | 0.547 | 0.269 | 0.177 | 0.197 |
| 0.066 | 0.615 | 0.325 | 0.202 | 0.212 |
| 0.224 | 0.686 | 0.305 | 0.220 | 0.233 |
| 0.076 | 0.545 | 0.278 | 0.157 | 0.169 |
| 0.098 | 0.606 | 0.305 | 0.234 | 0.243 |
| 0.136 | 0.633 | 0.326 | 0.203 | 0.209 |
| 0.106 | 0.597 | 0.274 | 0.234 | 0.222 |
| 0.150 | 0.558 | 0.271 | 0.308 | 0.246 |
| 0.090 | 0.635 | 0.415 | 0.272 | 0.388 |
| 0.070 | 0.559 | 0.280 | 0.191 | 0.130 |
| 0.095 | 0.590 | 0.278 | 0.172 | 0.201 |

| 0.084 | 0.597 | 0.315 | 0.175 | 0.216 |
| --- | --- | --- | --- | --- |
| 0.110 | 0.654 | 0.365 | 0.225 | 0.269 |
| 0.072 | 0.588 | 0.303 | 0.205 | 0.268 |
| 0.128 | 0.626 | 0.343 | 0.205 | 0.236 |
| 0.114 | 0.607 | 0.308 | 0.263 | 0.258 |

| T_helper_cells | Tfh | Th1_cells | Th2_cells | TIL | Treg |
| --- | --- | --- | --- | --- | --- |
| 0.418 | 0.145 | 0.211 | 0.248 | 0.289 | 0.532 |
| 0.452 | 0.103 | 0.178 | 0.171 | 0.278 | 0.511 |
| 0.524 | 0.233 | 0.114 | 0.178 | 0.278 | 0.537 |
| 0.540 | 0.064 | 0.265 | 0.246 | 0.358 | 0.544 |
| 0.417 | 0.126 | 0.110 | 0.178 | 0.295 | 0.514 |
| 0.518 | 0.117 | 0.129 | 0.181 | 0.331 | 0.528 |
| 0.453 | 0.132 | 0.198 | 0.276 | 0.325 | 0.527 |
| 0.627 | 0.525 | 0.215 | 0.224 | 0.435 | 0.533 |
| 0.285 | 0.090 | 0.142 | 0.225 | 0.277 | 0.534 |
| 0.491 | 0.143 | 0.148 | 0.219 | 0.319 | 0.555 |
| 0.436 | 0.113 | 0.120 | 0.195 | 0.307 | 0.527 |
| 0.566 | 0.164 | 0.147 | 0.219 | 0.347 | 0.544 |
| 0.372 | 0.153 | 0.167 | 0.201 | 0.321 | 0.552 |
| 0.371 | 0.149 | 0.089 | 0.187 | 0.273 | 0.536 |
| 0.423 | 0.084 | 0.174 | 0.191 | 0.317 | 0.542 |
| 0.480 | 0.162 | 0.181 | 0.197 | 0.328 | 0.537 |
| 0.488 | 0.221 | 0.152 | 0.109 | 0.323 | 0.557 |
| 0.506 | 0.182 | 0.259 | 0.250 | 0.409 | 0.544 |
| 0.402 | 0.048 | 0.200 | 0.246 | 0.333 | 0.539 |
| 0.631 | 0.356 | 0.120 | 0.158 | 0.344 | 0.536 |
| 0.416 | 0.052 | 0.293 | 0.181 | 0.319 | 0.530 |
| 0.775 | 0.409 | 0.073 | 0.202 | 0.355 | 0.555 |
| 0.551 | 0.301 | 0.131 | 0.258 | 0.330 | 0.546 |
| 0.376 | 0.098 | 0.111 | 0.242 | 0.262 | 0.544 |
| 0.646 | 0.150 | 0.280 | 0.260 | 0.405 | 0.575 |
| 0.372 | 0.136 | 0.201 | 0.207 | 0.286 | 0.552 |
| 0.703 | 0.283 | 0.145 | 0.216 | 0.383 | 0.546 |
| 0.399 | 0.117 | 0.145 | 0.164 | 0.341 | 0.531 |
| 0.631 | 0.152 | 0.239 | 0.229 | 0.339 | 0.561 |
| 0.650 | 0.167 | 0.237 | 0.250 | 0.401 | 0.577 |
| 0.391 | 0.268 | 0.109 | 0.222 | 0.331 | 0.564 |
| 0.558 | 0.201 | 0.155 | 0.214 | 0.320 | 0.542 |
| 0.605 | 0.127 | 0.225 | 0.292 | 0.420 | 0.563 |
| 0.605 | 0.147 | 0.179 | 0.167 | 0.341 | 0.557 |
| 0.446 | 0.178 | 0.199 | 0.253 | 0.312 | 0.535 |
| 0.547 | 0.136 | 0.122 | 0.254 | 0.315 | 0.547 |
| 0.476 | 0.123 | 0.195 | 0.237 | 0.362 | 0.555 |
| 0.592 | 0.196 | 0.125 | 0.214 | 0.322 | 0.546 |
| 0.450 | 0.282 | 0.176 | 0.337 | 0.285 | 0.532 |
| 0.384 | 0.149 | 0.156 | 0.237 | 0.327 | 0.541 |
| 0.524 | 0.228 | 0.104 | 0.181 | 0.289 | 0.521 |
| 0.497 | 0.148 | 0.090 | 0.210 | 0.297 | 0.526 |
| 0.654 | 0.146 | 0.183 | 0.151 | 0.325 | 0.537 |
| 0.556 | 0.057 | 0.229 | 0.215 | 0.354 | 0.546 |
| 0.574 | 0.187 | 0.243 | 0.212 | 0.388 | 0.554 |
| 0.305 | 0.399 | 0.184 | 0.235 | 0.347 | 0.555 |
| 0.455 | 0.145 | 0.220 | 0.223 | 0.309 | 0.538 |
| 0.560 | 0.490 | 0.184 | 0.282 | 0.378 | 0.551 |
| 0.754 | 0.331 | 0.155 | 0.300 | 0.514 | 0.569 |

| 0.473 | 0.123 | 0.175 | 0.246 | 0.319 | 0.548 |
| --- | --- | --- | --- | --- | --- |
| 0.391 | 0.137 | 0.204 | 0.188 | 0.297 | 0.486 |
| 0.599 | 0.418 | 0.134 | 0.266 | 0.336 | 0.512 |
| 0.567 | 0.144 | 0.132 | 0.183 | 0.305 | 0.535 |
| 0.804 | 0.120 | 0.232 | 0.191 | 0.406 | 0.541 |
| 0.410 | 0.110 | 0.126 | 0.189 | 0.282 | 0.518 |
| 0.477 | 0.122 | 0.146 | 0.230 | 0.317 | 0.536 |
| 0.354 | 0.182 | 0.163 | 0.158 | 0.277 | 0.513 |
| 0.437 | 0.067 | 0.185 | 0.148 | 0.246 | 0.510 |
| 0.443 | 0.077 | 0.204 | 0.180 | 0.282 | 0.515 |
| 0.866 | 0.334 | 0.274 | 0.262 | 0.497 | 0.580 |
| 0.593 | 0.154 | 0.182 | 0.297 | 0.334 | 0.559 |
| 0.649 | 0.194 | 0.233 | 0.188 | 0.397 | 0.513 |
| 0.809 | 0.137 | 0.205 | 0.235 | 0.456 | 0.555 |
| 0.772 | 0.470 | 0.209 | 0.215 | 0.410 | 0.526 |
| 0.483 | 0.091 | 0.084 | 0.187 | 0.319 | 0.521 |
| 0.509 | 0.271 | 0.274 | 0.178 | 0.383 | 0.559 |
| 0.488 | 0.113 | 0.087 | 0.228 | 0.294 | 0.524 |
| 0.489 | 0.130 | 0.159 | 0.202 | 0.300 | 0.541 |
| 0.594 | 0.144 | 0.188 | 0.273 | 0.397 | 0.565 |
| 0.524 | 0.227 | 0.163 | 0.158 | 0.368 | 0.540 |
| 0.512 | 0.153 | 0.175 | 0.181 | 0.352 | 0.530 |
| 0.488 | 0.111 | 0.231 | 0.166 | 0.312 | 0.536 |
| 0.785 | 0.227 | 0.122 | 0.210 | 0.349 | 0.542 |
| 0.612 | 0.117 | 0.164 | 0.307 | 0.355 | 0.553 |
| 0.358 | 0.256 | 0.047 | 0.177 | 0.318 | 0.543 |
| 0.654 | 0.081 | 0.191 | 0.196 | 0.312 | 0.537 |
| 0.581 | 0.146 | 0.114 | 0.224 | 0.330 | 0.549 |
| 0.699 | 0.182 | 0.137 | 0.168 | 0.416 | 0.538 |
| 0.539 | 0.170 | 0.218 | 0.306 | 0.324 | 0.532 |
| 0.465 | 0.134 | 0.190 | 0.147 | 0.278 | 0.509 |
| 0.333 | 0.228 | 0.227 | 0.131 | 0.335 | 0.532 |
| 0.598 | 0.081 | 0.144 | 0.237 | 0.322 | 0.556 |
| 0.465 | 0.256 | 0.160 | 0.260 | 0.413 | 0.590 |
| 0.230 | 0.099 | 0.097 | 0.231 | 0.314 | 0.525 |
| 0.473 | 0.093 | 0.076 | 0.171 | 0.307 | 0.535 |
| 0.421 | 0.141 | 0.172 | 0.207 | 0.316 | 0.548 |
| 0.821 | 0.235 | 0.157 | 0.209 | 0.400 | 0.553 |
| 0.580 | 0.178 | 0.161 | 0.204 | 0.356 | 0.535 |
| 0.432 | 0.251 | 0.100 | 0.221 | 0.312 | 0.533 |
| 0.728 | 0.224 | 0.230 | 0.305 | 0.379 | 0.533 |
| 0.549 | 0.202 | 0.268 | 0.148 | 0.330 | 0.540 |
| 0.401 | 0.142 | 0.063 | 0.155 | 0.266 | 0.509 |
| 0.741 | 0.120 | 0.113 | 0.201 | 0.405 | 0.562 |
| 0.453 | 0.094 | 0.163 | 0.240 | 0.332 | 0.548 |
| 0.596 | 0.162 | 0.239 | 0.189 | 0.373 | 0.546 |
| 0.660 | 0.046 | 0.120 | 0.260 | 0.330 | 0.547 |
| 0.777 | 0.252 | 0.172 | 0.198 | 0.485 | 0.499 |
| 0.409 | 0.082 | 0.185 | 0.209 | 0.281 | 0.503 |
| 0.381 | 0.486 | 0.083 | 0.252 | 0.337 | 0.529 |

| 0.596 | 0.099 | 0.186 | 0.224 | 0.346 | 0.535 |
| --- | --- | --- | --- | --- | --- |
| 0.820 | 0.171 | 0.176 | 0.209 | 0.447 | 0.562 |
| 0.620 | 0.194 | 0.230 | 0.216 | 0.398 | 0.522 |
| 0.788 | 0.193 | 0.154 | 0.219 | 0.379 | 0.556 |
| 0.704 | 0.103 | 0.139 | 0.232 | 0.366 | 0.551 |

| Type_I_IFN_Reponse | Type_II_IFN_Reponse |
| --- | --- |
| 0.441 | 0.341 |
| 0.467 | 0.384 |
| 0.462 | 0.449 |
| 0.458 | 0.467 |
| 0.426 | 0.393 |
| 0.455 | 0.370 |
| 0.454 | 0.350 |
| 0.497 | 0.389 |
| 0.438 | 0.547 |
| 0.461 | 0.512 |
| 0.501 | 0.362 |
| 0.447 | 0.515 |
| 0.416 | 0.421 |
| 0.415 | 0.370 |
| 0.472 | 0.431 |
| 0.462 | 0.431 |
| 0.487 | 0.435 |
| 0.625 | 0.667 |
| 0.417 | 0.489 |
| 0.546 | 0.423 |
| 0.454 | 0.527 |
| 0.552 | 0.603 |
| 0.514 | 0.326 |
| 0.431 | 0.369 |
| 0.499 | 0.527 |
| 0.416 | 0.421 |
| 0.556 | 0.632 |
| 0.527 | 0.465 |
| 0.547 | 0.501 |
| 0.464 | 0.696 |
| 0.460 | 0.530 |
| 0.443 | 0.488 |
| 0.507 | 0.548 |
| 0.418 | 0.639 |
| 0.442 | 0.437 |
| 0.417 | 0.400 |
| 0.520 | 0.511 |
| 0.569 | 0.422 |
| 0.457 | 0.444 |
| 0.385 | 0.444 |
| 0.380 | 0.258 |
| 0.460 | 0.419 |
| 0.457 | 0.384 |
| 0.471 | 0.345 |
| 0.504 | 0.469 |
| 0.447 | 0.414 |
| 0.624 | 0.556 |
| 0.404 | 0.345 |
| 0.517 | 0.498 |

| 0.454 | 0.425 |
| --- | --- |
| 0.446 | 0.347 |
| 0.444 | 0.447 |
| 0.475 | 0.478 |
| 0.649 | 0.366 |
| 0.509 | 0.345 |
| 0.493 | 0.398 |
| 0.427 | 0.342 |
| 0.582 | 0.380 |
| 0.571 | 0.436 |
| 0.760 | 0.492 |
| 0.480 | 0.530 |
| 0.477 | 0.387 |
| 0.467 | 0.500 |
| 0.483 | 0.383 |
| 0.477 | 0.494 |
| 0.490 | 0.466 |
| 0.471 | 0.398 |
| 0.467 | 0.364 |
| 0.519 | 0.509 |
| 0.478 | 0.361 |
| 0.463 | 0.410 |
| 0.603 | 0.441 |
| 0.550 | 0.486 |
| 0.474 | 0.537 |
| 0.470 | 0.385 |
| 0.470 | 0.358 |
| 0.500 | 0.382 |
| 0.499 | 0.541 |
| 0.437 | 0.533 |
| 0.445 | 0.377 |
| 0.408 | 0.464 |
| 0.410 | 0.378 |
| 0.635 | 0.564 |
| 0.454 | 0.310 |
| 0.417 | 0.369 |
| 0.458 | 0.467 |
| 0.503 | 0.518 |
| 0.552 | 0.407 |
| 0.463 | 0.508 |
| 0.488 | 0.394 |
| 0.732 | 0.526 |
| 0.449 | 0.390 |
| 0.509 | 0.534 |
| 0.556 | 0.408 |
| 0.497 | 0.434 |
| 0.443 | 0.405 |
| 0.493 | 0.391 |
| 0.416 | 0.367 |
| 0.454 | 0.524 |

| 0.497 | 0.426 |
| --- | --- |
| 0.472 | 0.498 |
| 0.467 | 0.386 |
| 0.476 | 0.495 |
| 0.491 | 0.482 |
